# Nitric Oxide Controls Constitutive Freezing Tolerance in Arabidopsis by Attenuating the Levels of Osmoprotectants, Stress-Related Hormones and Anthocyanins

**DOI:** 10.1038/s41598-018-27668-8

**Published:** 2018-06-18

**Authors:** Álvaro Costa-Broseta, Carlos Perea-Resa, Mari-Cruz Castillo, M. Fernanda Ruíz, Julio Salinas, José León

**Affiliations:** 1 Instituto de Biología Molecular y Celular de Plantas, (Consejo Superior de Investigaciones Científicas–Universidad Politécnica de Valencia), 46022 Valencia, Spain; 2 Departamento de Biotecnología Microbiana y de Plantas, Centro de Investigaciones Biológicas, CSIC, 28040 Madrid, Spain; 3 Present Address: Department of Molecular Biology, Massachusetts General Hospital, Boston, MA 02214 USA

## Abstract

Plant tolerance to freezing temperatures is governed by endogenous constitutive components and environmental inducing factors. Nitric oxide (NO) is one of the endogenous components that participate in freezing tolerance regulation. A combined metabolomic and transcriptomic characterization of NO-deficient *nia1,2noa1–2* mutant plants suggests that NO acts attenuating the production and accumulation of osmoprotective and regulatory metabolites, such as sugars and polyamines, stress-related hormones, such as ABA and jasmonates, and antioxidants, such as anthocyanins and flavonoids. Accordingly, NO-deficient plants are constitutively more freezing tolerant than wild type plants.

## Introduction

Plants ability to tolerate below zero temperatures relies on complex and varied processes that involve the accumulation of endogenous as well as inducible components often regulated by environmental factors. The endogenous components that favor constitutive freezing tolerance have been extensively studied and mainly refer to metabolites with osmoprotective activities to limit freeze-induced dehydration and to avoid ice nucleation inside the cells^1^, with hormonal activities^2^ or with antioxidant functions^3^. Moreover, other regulatory molecules such as polyamines, lipids, reactive oxygen species and nitric oxide (NO) have also been described to be involved in freezing tolerance^4–9^.

NO is endogenously produced in diverse living organisms and regulates a wide array of biological processes including many responses of plants to environmental abiotic and biotic stresses^10,11^. In plants, NO is generated through both oxidative and reductive biosynthetic pathways, which are enhanced under stress^12,13^. NO is a free radical that react with metals and reactive oxygen species (ROS)^14^, thus contributing to redox homeostasis and alleviating oxidative stress. It has been also reported that NO improves the antioxidant capacity of plants^15^. However, NO can potentiate or attenuate oxidative stress in plants when acts either in a chronic or acute mode^16^. Besides redox-related functions, NO has also the potential of triggering post-translational modifications (PTMs) of many target proteins, which then display often altered function, activity, stability and/or subcellular localization. NO induces S-nitrosylation of Cysteine and nitration of Tyrosine^17^, but also ubiquitylation of Lysine and phosphorylation of Serine, Threonine and Tyrosine^18^. In addition, many of those PTMs also alter the stability of the target proteins through the regulation of proteolytic degradation^19^.

Genetic approaches using the Arabidopsis *nia1,2* mutant plants, impaired in nitrate reductase-mediated synthesis of NO, as well as pharmacological treatments with NO donors, inhibitors and scavengers allowed proposing a role for NO in constitutive freezing tolerance in Arabidopsis^8,9,20^. However, the contribution of nitrate-independently produced NO to low temperature responses has been barely addressed. In this work, we have studied the constitutive freezing tolerance and the capacity to cold acclimate of triple *nia1,2noa1–2* mutant plants that are impaired in nitrate-dependent and nitrate-independent NOA1-associated pathways and thus contain very low levels of NO^21^. Dissection of the regulatory roles exerted by NO on constitutive freezing tolerance has been aided by an integrative approach combining metabolomic and transcriptomic analyses.

## Results

### The transcriptome of NO-deficient *nia1,2noa1–2* mutant plants is enriched in cold-related transcripts

**Figure 1:**
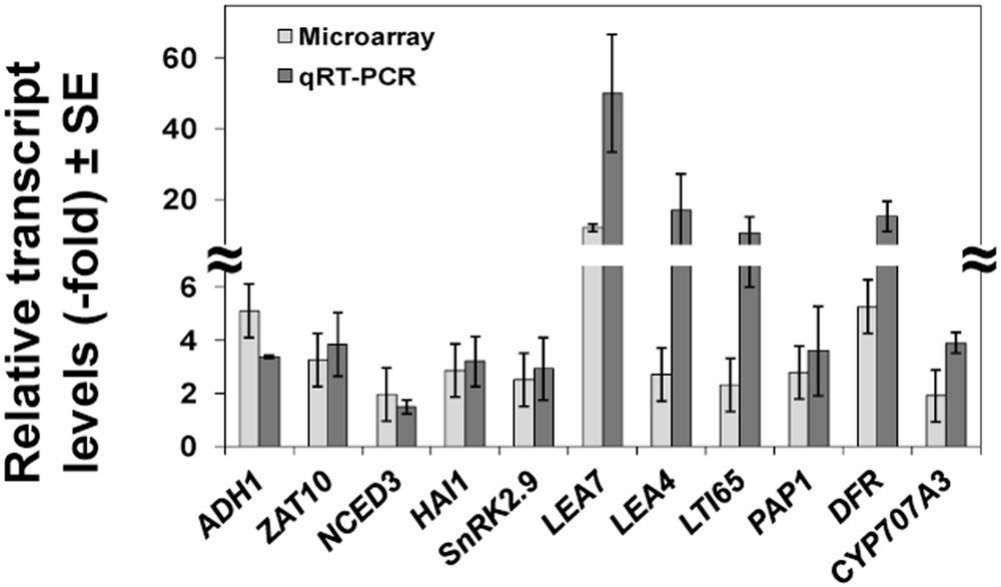
Levels of cold-inducible transcripts in Col-0 and *nia1,2noa1-2* plants. Comparative transcript analysis based on microarray data and RT-qPCR of wild-type Col-0 and NO-deficient *nia1,2noa1-2* plants. Ratio (*nia1,2noa1,2*/Col-0) values of RT-qPCR analysis are the mean of three independent biological replicates ± standard deviation.

We previously reported that *nia1,2noa1–2* mutant plants, carrying mutations in both NIA1 and NIA2 nitrate reductases, as well as in the NO-Associated 1 (NOA1) protein, accumulated very low levels of endogenous NO under control and stressed conditions^21^. The strong NO deficiency of the mutant plants correlated with hypersensitivity to ABA in seed germination, stomata closure and tolerance to dehydration^21^. Intriguingly, our transcriptome analysis of *nia1,2noa1–2* mutants grown at 20 °C (GEO identification number GSE41958)^22^ revealed that around 20% (88/465) of the genes that were up-regulated in the mutant compared to wild-type plants (>2-fold; FDR <0.05) had been related to cold responses^23,24^. Among those genes (Table S1), some coded for Late Embryogenesis Abundant (LEA) proteins and for transcription factors belonging to the ERF/DREB, Zinc finger and WRKY families. Cold-induced *BCH2* and *NCED3* genes, encoding β-carotene hydroxylase 2 and 9-cis-epoxycarotenoid dioxygenase 3 enzymes involved in ABA biosynthesis, as well as *LOX4* and *OPR1* coding for jasmonate biosynthesis enzymes were also up-regulated in NO-deficient plants (Table S1). ABA and jasmonates have been reported to positively regulate freezing tolerance in Arabidopsis^25,26^. Furthermore, a Gene Ontology analysis performed with the *Arabidopsis thaliana* dataset of the Gene Ontology Consortium (http://www.geneontology.org/) showed that 7 out of 19 and 15 out of 67 genes (20- and 12-fold enrichment with p-values of 2.51E-04 and 1.71E-08) involved in the anthocyanin and flavonoid metabolism functional categories, respectively, were up-regulated in the NO-deficient mutant plants. Accordingly, we found the anthocyanin and flavonoid biosynthesis and metabolism genes *CHS*, *F3´H/TT6*, *DFR/TT3*, *PAP1/MYB75* and *UF3GT* among the cold-induced genes that were up-regulated in *nia1,2noa1–2* plants (Table S1). In addition, genes coding for SUS3, SSP2, and ADC2 enzymes involved in the biosynthesis of sugars and polyamines, respectively, were among the cold-inducible genes up-regulated in NO-deficient plants (Table S1). Sugars and polyamines had been reported to enhance plant-freezing tolerance^27–29^. To assess the robustness of the over-representation of cold-inducible genes detected in our transcriptomic analysis, the expression levels of 11 cold-induced genes, including *ADH1*, *ZAT10*, *NCED3*, *HAI1*, *SnRK2.9*, *LEA7*, *LEA4–5*, *LTI65*, *PAP1/MYB75*, *DFR* and *CYP707A3*, were determined by RT-qPCR in independent RNA samples from the triple *nia1,2noa1–2* mutant and Col-0 plants grown at 21 °C. In all cases, the transcript levels were significantly higher in mutant than in wild-type plants (Fig. 1a), thus validating the microarray data. These observations indicated that, under control conditions, NO functions as a negative regulator of cold-induced gene expression in Arabidopsis.

### Enhanced biosynthesis of ABA, JA and osmoprotective metabolites in NO-deficient plants

**Figure 2:**
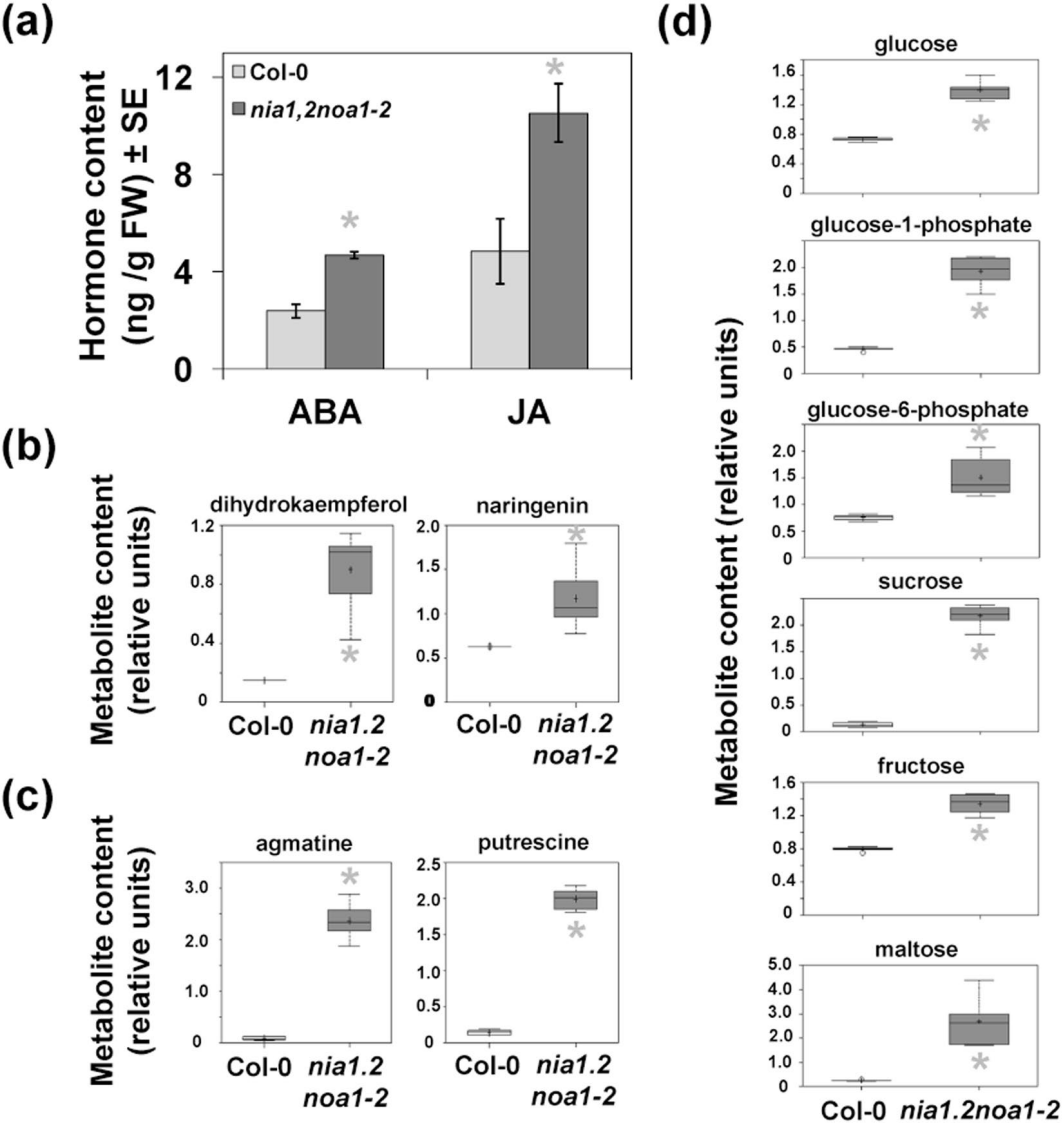
Levels of hormones and osmoprotective metabolites in Col-0 and *nia1,2noa1-2* plants. (**a**) Quantification of ABA and JA, (**b**) flavonoids/anthocyanins, (**c**) polyamines, and (**d**), sugars, was performed by GC- and LC-mass spectrometry. Hormone content values represent the mean values of four independent biological replicate samples for each genotype ± standard error. *Indicates significantly different with p-value < 0.05 in Student’s t-test. For the metabolomic analyses of the other metabolites, Welch’s two-sample t-test was used to identify biochemicals that differed significantly between experimental groups. An estimate of the false discovery rate (q-value) was calculated to take into account the multiple comparisons.

**Figure 3:**
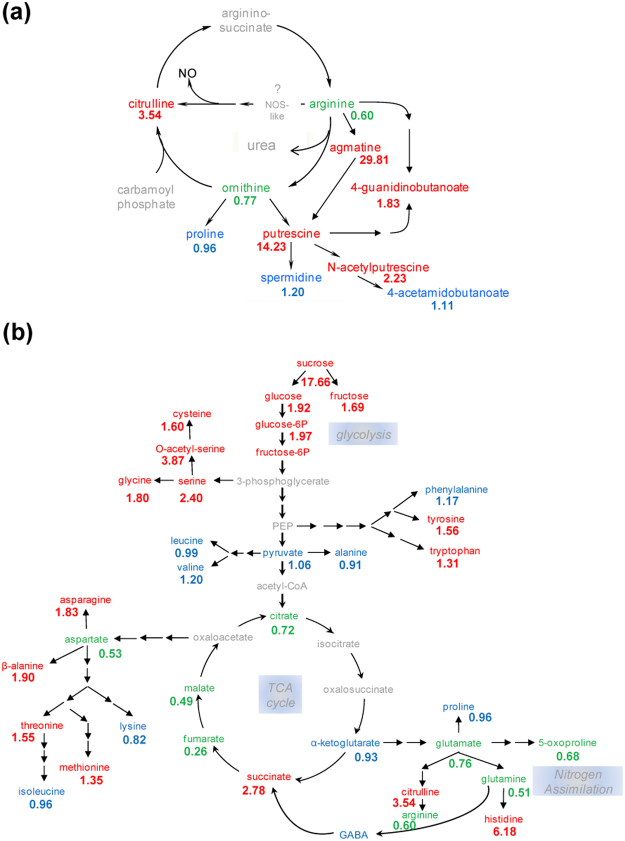
Glycolysis and TCA cycle metabolite ratios between *nia1,2noa1-2* and Col-0 plants. Metabolites in red and green were significantly more or less abundant in *nia1,2noa1-2* than in Col-0 plants, respectively. Metabolites in blue were not significantly changed. Values indicated for each metabolite are the mean of six independent replicates performed in the complete metabolomic analyses described in Table S2.

Data from microarray analyses strongly suggested that NO-deficient mutants should have increased levels of ABA, JA, anthocyanins, flavonoids, sugars and polyamines. Ultra Performance Liquid Chromatography-Mass Spectrometry analysis confirmed that, in fact, the levels of ABA and JA were around 2-fold higher in *nia1,2noa1–2* than in wild-type plants (Fig. 2a). On the other hand, a combination of GC-MS and LC-MS techniques allowed quantifying 180 biochemicals including amino acids, carbohydrates, lipids, cofactors and prosthetic groups, nucleotides and secondary metabolites in wild-type and NO-deficient mutant plants (Table S2). As expected from the microarray data, the endogenous levels of flavonoids, anthocyanins, polyamines and sugars were significantly higher in mutant than in wild-type plants (Fig. 2b–d). The content of the flavonoids/anthocyanins dihydrokaempferol and naringenin in mutant plants were around 6- and 2-fold higher than in wild-type plants, respectively (Fig. 2b; Table S2). Similarly, the polyamines agmatine and putrescine were around 30- and 14-fold higher, respectively, in mutant than in wild-type plants (Fig. 2c; Table S2). Finally, the levels of glucose, glucose-1-phosphate, glucose-6-phosphate, sucrose, fructose and maltose were increased from 2- to 18-fold in *nia1,2noa1–2* when compared to wild-type plants (Fig. 2d; Table S2). As shown in Fig. 3a, the increased levels of polyamines correlated with reduced content of arginine and ornithine and increased levels of citrulline. On the other hand, the increased levels of sugars in *nia1,2noa1–2* plants reflected a general accumulation of glycolisis metabolites and phosphoglycerate-derived amino acids of the serine family (Fig. 3b). In turn, metabolites of the tricarboxylic acids (TCA) cycle were significantly less abundant in *nia1,2noa1–2* than in wild-type plants (Fig. 3b). Accordingly, the levels of α-ketoglutarate-derived amino acids of the glutaminate synthetase-glutamine oxoglutarate aminotransferase (GS-GOGAT) cycle were also lowered in *nia1,2noa1–2* plants (Fig. 3b), likely as a reflection of the impaired nitrate assimilation of the mutant plants. In summary, NO seems to exert a metabolic brake in the production of ABA, JA, anthocyanins, flavonoids, sugars and polyamines under standard conditions.

### Increased levels of antioxidant metabolites in *nia1,2noa1–2* plants

**Figure 4:**
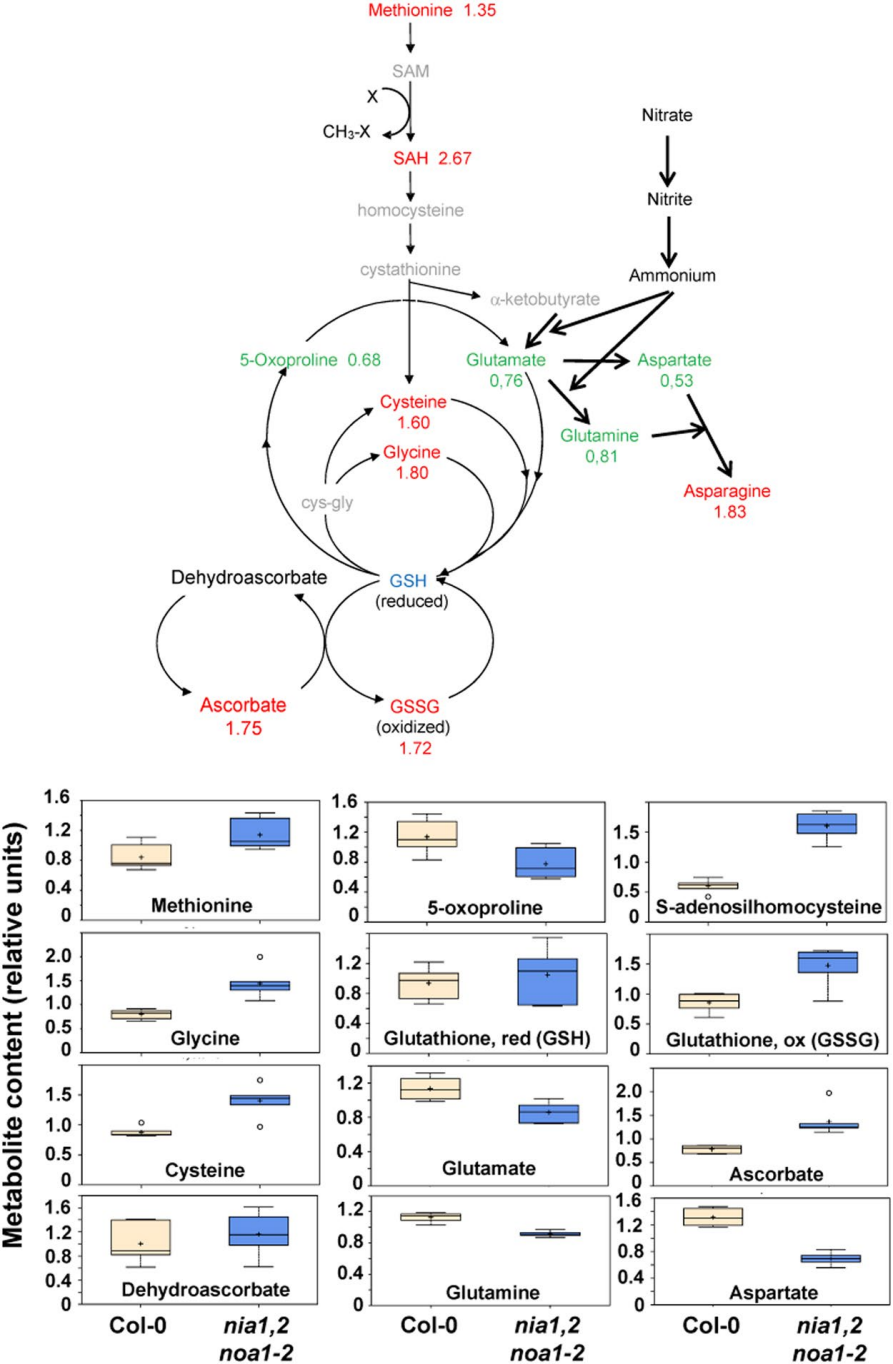
Endogenous content of ascorbate-glutathione cycle metabolites in wild type and NO-deficient plants. A diagram of the ascorbate-glutathione cycle is shown at top of the figure. Metabolites in red and green were significantly more or less abundant in *nia1,2noa1-2* than in Col-0 plants, respectively. The box plots corresponding to the metabolites significantly different in both genotypes are shown in the bottom part of the figure. Values indicated for each metabolite are the mean of six independent replicates performed in the complete metabolomic analyses described in Table S2.

As shown in Table S2, the ascorbate and oxidized glutathione (GSSG) were both elevated in *nia1,2noa1–2* plants. Moreover, other metabolites with antioxidant activity such as the flavonoids dihydrokaempferol and naringenin (Fig. 2b) as well as sinapate (Table S2) accumulated also in NO-deficient plants. We also found around 3-fold accumulation of the oxylipins 9-hydroxyoctadecadienoic acid (9-HODE) and 13-hydroxyoctadecadienoic acid (13-HODE) (Table S2), which can be synthesized enzymatically by lipoxygenases but also non-enzymatically from ROS^30^, and are considered good markers of oxidative stress^31^. Thee data strongly suggested that NO-deficient *nia1,2noa1–2* mutant plants were subjected to constitutive oxidative stress. Under those conditions, the ascorbate-glutathione cycle is in charge of detoxifying reactive oxygen species. As shown in Fig. 4, the increased levels of ascorbate and oxidized glutathione were accompanied by significant increases of glutathione precursors, such as methionine, S-adenosylhomocysteine, cysteine and glycine, as well as by a reduced content of nitrogen-related amino acids including glutamate, glutamine and aspartate.

### NO negatively regulates constitutive freezing tolerance of Arabidopsis

**Figure 5:**
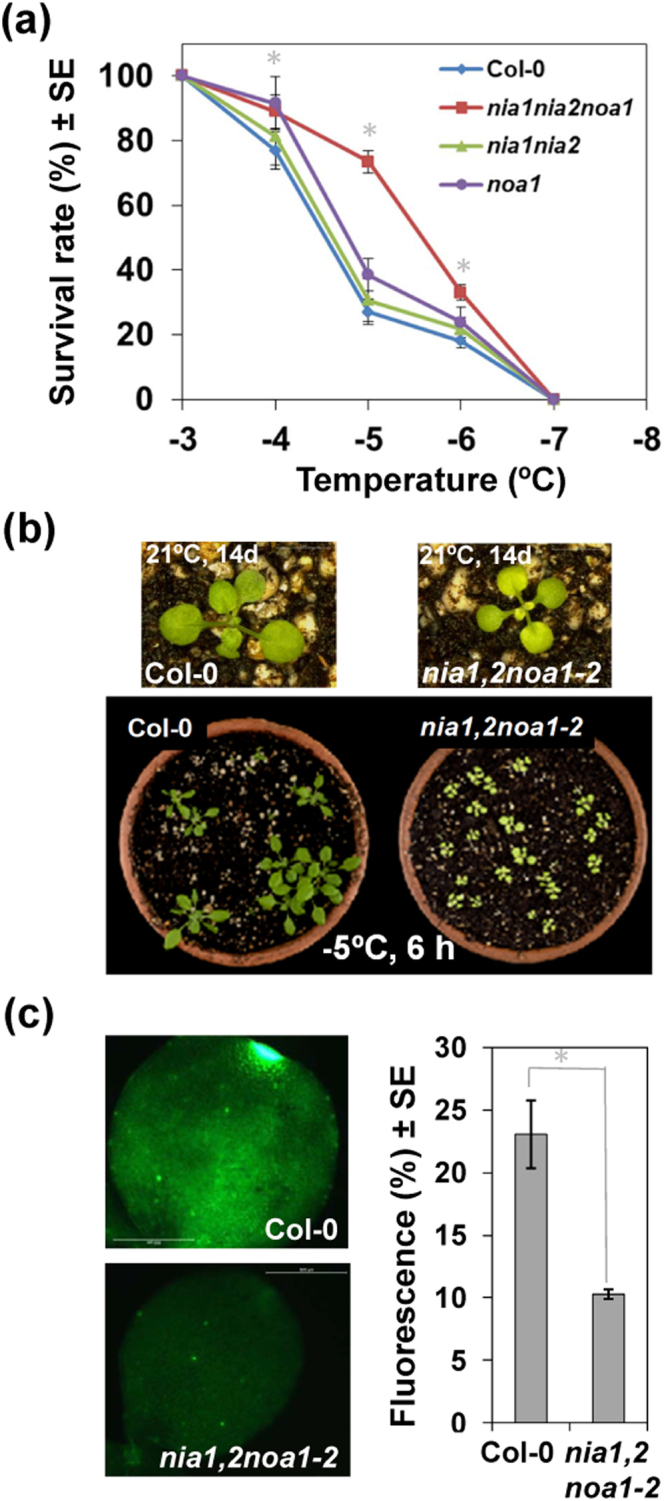
Constitutive freezing tolerance of Col-0 and *nia1,2noa1-2* plants. (**a**) Freezing tolerance of 2-week-old plants exposed for 6 h to the indicated freezing temperatures was estimated as the percentage of plants surviving each specific temperature after 7 d of recovery under control conditions at 21 °C. Data are expressed as means of three independent experiments with around 50 plants each indicated genotype ± standard deviation. Asterisks indicate significant differences between *nia1,2noa1–2* and wild-type plants (p-value < 0.05). (**b**) Upper panels show individual plants of wild type and mutant genotypes before freezing to show the difference in size. The bottom panel shows a representative image of plants from both genotypes after freezing at −5 °C and recovery at standard growing temperature for additional 7 days. (**c**) NO levels in Col-0 and *nia1,2noa1–2* plants. Plants were maintained at standard growing conditions for 14 days. The fluorescence of DAF-FM DA-treated plants was detected by confocal microscopy. Shown images are representative of four to six different analyzed plants per genotype and condition, and the quantification values are the mean ± standard error. *Indicates significant differences between *nia1,2noa1–2* and wild-type plants (p-value < 0.05).

The results described above strongly suggested that NO should have a negative role in the constitutive freezing tolerance of Arabidopsis. To test this possibility, we analyzed the constitutive freezing tolerance of 2-week-old wild-type and *nia1,2noa1–2* plants. Freezing tolerance was determined as the percentage of surviving plants after exposure to different freezing temperatures for 6 h. Figure 5a shows that triple *nia1nia2noa1–2* mutant plants displayed significantly greater freezing tolerance than did wild-type plants, the LT_50_ (temperature that causes 50% lethality) value being −5.6 °C and −4.5 °C, respectively. However, the double *nia1nia2* mutant plants (LT_50_ −4.6 °C) were not significantly different than wild type plants and the single *noa1–2* mutant plants were slightly more tolerant (LT_50_ −4.8 °C) than wild type plants. Despite *nia1,2noa1–2* plants being slightly delayed in their development compared to Col-0 plants, the increased freezing tolerance manifested by the mutant with respect to the wild-type plants was very apparent (Fig. 5b). The endogenous NO levels of wild type and mutant plants were measured by staining with the NO-specific fluorophore staining FAF-FM DA and we found that, as expected, *nia1,2noa1–2* plants contained significantly less NO than Col-0 plants (Fig. 5c). These results demonstrated that NO negatively regulates constitutive freezing tolerance in Arabidopsis, in all likelihood, by controlling the levels of osmoprotectant, hormones and redox metabolites.

## Discussion

Genetic approaches using the Arabidopsis *nia1,2* mutant plants suggested that NO is required for the adequate constitutive freezing tolerance and also for the full development of the cold acclimation process^8,20^. Nevertheless, our data reported here using *nia1,2noa1–2* triple mutant plants, which are impaired not only in nitrate reductase-mediated but also in NOA1-associated production of NO^1^, showed that *nia1,2noa1–2* plants were constitutively more tolerant to freezing than wild-type plants. Remarkably, neither the double *nia1,2* nor the single *noa1–2* mutant plants were significantly more tolerant than wild type plants under our freezing conditions. The discrepancy between our data and those obtained by using *nia1,2* plants could be due to several reasons. First, the experimental conditions employed to grow plants, which have been described to be critical for correct hormone signaling in cold acclimation^32^, were very different. Indeed, previous work with *nia1,2* plants was performed using plants grown in Petri dishes on sucrose supplemented MS media^8,20^, which implies that they were exposed to a high relative humidity. Our freezing tolerance experiments, however, were carried out with plants grown on soil. As an indicator of the differences in the performance of both experimental systems, the increase in NO content was significant during the first hour^20^ or after 24 h^8^ of exposure to 4 °C in wild-type plants. Yet, under our experimental conditions, wild-type plants registered a very slight likely non-significant increase in the endogenous NO content by 1 day at 4 °C but large increases were detected only after 7 days under cold conditions, when acclimation has been reported to be completed^33,34^. As expected, the cold-induced increase in NO was largely abolished in *nia1,2noa1–2* plants. On the other hand, our previous analyses comparing the transcriptomes of *nia1,2noa1–2* and *nia1,2* to that of wild-type plants pointed to a potentiated enhancement of the differentially expressed genes in the triple mutant^22^ (GEO identification number GSE41958), which correlated with the stronger reduction in NO content^21^. Together, these data suggest that the effects of NO-deficiency were additive in the triple mutant. Finally, another indication of *nia1,2* and *nia1,2noa1–2* mutants being different in terms of cold response comes from their different accumulation of proline, whose content has been shown to positively correlate with freezing tolerance in Arabidopsis^35,36^. Regarding this, whereas Zhao *et al*.^8^ observed an increase in proline content in wild-type but not in *nia1,2* plants exposed to 4 °C, we did not find significant differences in proline content between *nia1,2noa1–2* and wild-type plants. Nevertheless, although increases in proline content have been reported during cold acclimation in different Arabidopsis accessions, there was no correlation with enhanced freezing tolerance^37^. Actually, our results revealed lower levels of proline metabolites, such as N-acetylproline, trans-4-hydroxyproline and 5-oxoproline, in *nia1,2noa1–2* than in wild-type plants.

The increased constitutive freezing tolerance of NO-deficient plants described in this work is fully consistent with the changes observed when the transcriptomes and metabolomes of *nia1,2noa1–2* and wild-type plants, grown under control conditions, were compared. The up-regulated transcriptome of n*ia1,2noa1–2* plants contained a large number of transcripts that have been previously reported to be cold-induced. In addition, mutant plants also accumulated high amounts of osmoprotective metabolites such as and sugars, polyamines, and antioxidant metabolites, including anthocyanins and flavonoids, which, in all likelihood, limit the impact of the freezing imposed damage. Many of the cold-inducible transcripts that were up-regulated in *nia1,2noa1–2* plants were also significantly more expressed in *nia1,2* plants (GEO identification number GSE41958), thus suggesting that the metabolic changes could be determinant for the enhanced freezing tolerance displayed by the triple mutant. For instance, *nia1,2noa1–2* plants contained 14 times the content of the polyamine putrescine in wild-type plants. These extremely high levels of putrescine are higher than those detected in the best Arabidopsis lines over-expressing the polyamine biosynthetic gene *ADC2*^38^, which have been reported to display increased freezing tolerance^39^. Thus, our findings suggest that the elevated levels of putrescine in the NO-deficient plants are relevant for their enhanced freezing tolerance. Despite the relevance of polyamines, the constitutive freezing tolerance of the triple mutant plants seems to be also greatly influenced by their endogenous levels of sugars, with 17-fold higher sucrose content than wild-type plants. A significantly higher capacity for sucrose synthesis has been reported in cold-tolerant over cold-sensitive Arabidopsis accessions^40^. Although the increased content of polyamines and sugars might be itself enough to explain the increased freezing tolerance of the NO-deficient plants, the triple mutant plants also contained augmented anthocyanin and flavonoid levels. Because the photosynthesis rate decreases at low temperature, the damage caused by photoinhibition under an excess of irradiance energy likely compromise the viability of the plant. It has been extensively reported that flavonoids and anthocyanins exert key antioxidant protection and light trapping that prevents chlorophyll excitation in chloroplasts^28,41–43^^,^. Furthermore, we found that *nia1,2noa1–2* plants contained significantly higher content of ABA and JA than wild-type plants. The characterization of the freezing sensitive phenotype of the *frs1* mutant, which resulted to be an allele of the *aba3* biosynthetic mutant, demonstrated that ABA mediates the constitutive freezing tolerance of Arabidopsis^44^. On the other hand, it has been reported that the exogenous application of jasmonates enhance the constitutive freezing tolerance of Arabidopsis, whereas blocking the endogenous jasmonate biosynthesis rendered plants hypersensitive to freezing stress^25^. Therefore, the high levels of ABA and JA in *nia1,2noa1–2* plants should also contribute to their enhanced constitutive freezing tolerance. Hence, our data point out that NO functions as a negative regulator of the constitutive freezing tolerance in Arabidopsis by attenuating the production of osmoprotective and antioxidant metabolites and also by altering hormone homeostasis.

Although the mechanisms by which NO regulate gene expression, protein homeostasis and metabolism are still mostly unknown, we can predict that a significant contribution may come from NO-triggered post-translational modifications (PTMs) such S-nitrosylation of cysteines and nitration of tyrosines^17^. Many of the nitrated proteins identified previously in an *in vivo* proteomic screening^45^ were involved in primary metabolism of C, N and S. Among those proteins we found Glyceraldehyde phosphate dehydrogenases, Malate dehydrogenases, Formate dehydrogenases, Enolase, Fructose-1,6-bisphosphatase and Carbonic anhydrases 1 and 2, all of them involved in several pathways related to carbohydrate synthesis and metabolism leading to sugars. The lack of nitration of key Y residues in NO-deficient plants may be relevant to explain the differential accumulation of sugars and phenylpropanoid-derived metabolites found in *nia1,2noa1–2* plants (Table S2). Regarding N metabolism, Glutamine synthetases and Aspartate aminotransferase were also identified as nitrated proteins^45^. Whether changes in these enzymes function upon NO-triggered PTMs is relevant for constitutive freezing tolerance of plants will require further work. Finally, several S metabolism-related enzymes, such as Methionine synthase, Adenosyl homocysteinase 1, S-adenosyl methionine synthetase 2 and Cysteine synthases (OAS-TL A and C), were also identified as nitrated^45^. These enzymes are involved in the sulfate assimilation pathway that feeds the biosynthesis of glutathione. The altered activities of those enzymes upon nitration might be relevant for the function of the redox buffering function exerted by glutathione. Moreover, L-Ascorbate peroxidase 1 was also identified as nitrated^45^. The ascorbate-glutathione cycle together with catalases, superoxide dismutases and peroxidases are relevant systems in detoxifying reactive oxygen in stressed plants^46^, and it has been reported that several of the enzymes involved in that process undergo NO-related PTMs^47^. This mechanism based on NO triggered PTMs would represent a potential control point of oxidative stress, which participates in plant responses to a wide array of environmental stresses.

**Figure 6:**
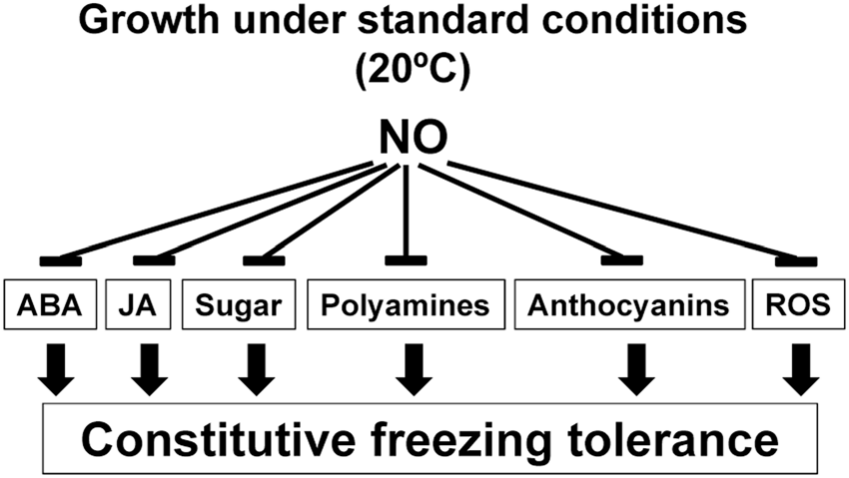
Model of NO involvement in the regulation of constitutive freezing tolerance. Blunt ended and black solid arrows represent negative and positive regulation on freezing tolerance, respectively. ABA, JA and ROS mean Abscisic acid, Jasmonates and Reactive Oxygen Species, respectively.

The data presented in this work suggest that NO would scavenge reactive oxygen species and also would attenuate metabolic changes leading to increases in the levels of polyamines, sugars, anthocyanins, flavonoids, ABA and JA that are essential for the adequate constitutive freezing tolerance (Fig. 6), thus explaining why NO-deficient plants display an increased constitutively freezing tolerance.

## Methods

### Plant materials and growth conditions

The *Arabidopsis thaliana* Col-0 ecotype was the wild-type genetic background used in this work. The triple *nia1,2noa1–2* mutant seeds were obtained by crossing *nia1nia2* (N2356) and *noa1–2* (SAIL_507_E11), obtained from NASC seed bank, as previously reported^21^. Genotyping by PCR and Cleaved Amplified Polymorphic Sequences (CAPS) with specific primers (Table S3) were used to select triple homozygous mutant plants^21^. Seeds were grown in soil mixture or MS media as previously described^48^ and experiments were performed with 2-week old plants.

### Freezing tolerance assays

Seeds from the different genotypes were sown in soil-containing pots and allowed to develop for 7 days. Then, several plants for each pot were removed in order to leave a similar number (25–30) of plants, homogenously distributed in all pots. Before being subjected to freezing temperatures, plants were exposed for 1 h to 4 °C in the freezing chamber. Then, temperature was progressively decreased (−1 °C/30 min) until reaching the indicated freezing temperatures. After exposing plants to the appropriate freezing temperature for 6 h, temperature was gradually increased to 4 °C (+1 °C/30 min). One hour later, plants were transferred to 20 °C under long-day light regime for recovering and subsequent survival evaluation 7 days later.

### RNA isolation and quantitative transcript analysis

Total RNA was isolated from 10 to 12 days old seedlings, separated, and analyzed by RT-qPCR techniques as previously described^48^ with specific primers (Table S3). The identity and full name annotation of every gene analyzed in this work is as follows: ALCOHOL DEHYDROGENASE 1 (ADH1, AT1G77120); SALT TOLERANCE ZINC FINGER (STZ/ZAT10, AT1G27730); NINE-CIS-EPOXYCAROTENOID DIOXYGENASE 3 (NCED3, AT3G14440); HIGHLY ABA-INDUCED PP2C GENE 1 (HAI1/SAG113, AT5G59220); SNF1-RELATED PROTEIN KINASE 2.9 (SnRK2.9, AT2G23030); LATE EMBRYOGENESIS ABUNDANT 7 (LEA7,AT1G52690); LATE EMBRYOGENESIS ABUNDANT 4–5 (LEA4–5, AT5G06760); LOW-TEMPERATURE-INDUCED 65/RESPONSIVE TO DESICCATION 29B (LTI65/RD29B, AT5G52300); PRODUCTION OF ANTHOCYANIN PIGMENT 1/MYB DOMAIN PROTEIN 75ATMYB75/SUC-INDUCED ANTHOCYANIN ACCUMULATION 1 (PAP1/MYB75/SIAA1, AT1G56650); DIHYDROFLAVONOL 4-REDUCTASE (DFR/TT3, AT5G42800); CYTOCHROME P450, FAMILY 707, SUBFAMILY A, POLYPEPTIDE 3 (CYP707A3, AT5G45340); NITRATE REDUCTASE 1 (NR1/NIA1, AT1G77760); NITRATWE REDUCTASE 2 (NR2/NIA2, AT1G37130); NITRIC OXIDE ASSOCIATED PROTEIN 1 (NOA1/RIF1/SVR10, AT3G47450).

### NO detection by fluorescence and confocal microscopy

The endogenous levels of NO in shoots were determined by staining with 10 μM DAF-FM DA fluorescein as described^49^ with some modifications. Fluorescence was detected by confocal microscopy with a CLSM LEICA TCS SP5, using unchanged parameters for every measurement. The DAF-FM DA fluorescence intensities were analyzed using Adobe Photoshop by quantifying green pixels in 3 to 6 replicate images of every genotype and condition from three independent experiments. The mean value ± standard error is shown.

### Metabolomic analyses

The sample preparation process was carried out using the automated MicroLab STAR® system from Hamilton Company. Recovery standards were added prior to the first step in the extraction process for quality control (QC) purposes. Sample preparation was conducted by series of organic and aqueous extractions to remove the protein fraction while allowing maximum recovery of small molecules. The resulting extract was divided into two fractions; one for analysis by Liquid Chromatography (LC) and one for analysis by Gas Chromatography (GC). Samples were placed briefly on a TurboVap® (Zymark) to remove the organic solvent. Each sample was then frozen, dried under vacuum and prepared for either LC/MS or GC/MS. More details on the metabolomics methodology are described in the Methods data sheet in Table S2.

### Quantification of anthocyanins

Anthocyanins were spectrophotometrically determined in methanolic extracts by reading their absorbance at 530 nm as described^50^.

### Statistical analyses

For metabolomic analyses, following log transformation and imputation with minimum observed values for each compound, Welch’s two-sample t-test was used to identify biochemicals that differed significantly between experimental groups. An estimate of the false discovery rate (q-value) was calculated to take into account the multiple comparisons. Statistical analyses are performed with the program “R” http://cran.r-project.org/. Statistically significant differences in hormone quantification and transcript analyses were computed based on Student’s t-tests.

### Phytohormone quantification

Four independent biological replicate samples of around 150–200 mg fresh weight of either non-acclimated or cold-acclimated Col-0 and *nia1,2noa1–2* seedlings were suspended in 80% methanol-1% acetic acid containing internal standards and mixed by shaking during one hour at 4 °C. The extract was kept a −20 °C overnight, centrifuged, the supernatant dried in a vacuum evaporator, and the dry residue was dissolved in 1% acetic acid and passed through an Oasis HLB (reverse phase) column as described^51^. The dried eluate was dissolved in 5% acetonitrile-1% acetic acid, and the hormones were separated using an autosampler and reverse phase UHPLC chromatography (2.6 µm Accucore RP-MS column, 50 mm length x 2.1 mm i.d.; ThermoFisher Scientific) with a 5 to 50% acetonitrile gradient containing 0.05% acetic acid, at 400 µL/min over 14 min.

The phytohormones were analyzed with a Q-Exactive mass spectrometer (Orbitrap detector; ThermoFisher Scientific) by targeted Selected Ion Monitoring (SIM). The concentrations of hormones in the extracts were determined using embedded calibration curves and the Xcalibur 2.2 SP1 build 48 and TraceFinder programs. The internal standard for quantification of ABA was the deuterium-labelled hormone. For JA, dihydrojasmonate (dhJA) was used as internal standard.

## Electronic supplementary material

Supplemental Information

## References

[1] Janská A, Marsík P, Zelenková S, Ovesná J (2010). Cold stress and acclimation - what is important for metabolic adjustment?. Plant Biol (Stuttg).

[2] Eremina M, Rozhon W, Poppenberger B (2016). Hormonal control of cold stress responses in plants. Cell Mol Life Sci.

[3] Winkel-Shirley B (2002). Biosynthesis of flavonoids and effects of stress. Curr Opin Plant Biol.

[4] Cuevas JC (2008). Putrescine is involved in Arabidopsis freezing tolerance and cold acclimation by regulating abscisic acid levels in response to low temperature. Plant Physiol.

[5] Chen M, Thelen JJ (2016). Acyl-lipid desaturase 1 primes cold acclimation response in Arabidopsis. Physiol Plant.

[6] Takahashi D, Kawamura Y, Uemura M (2016). Cold acclimation is accompanied by complex responses of glycosylphosphatidylinositol (GPI)-anchored proteins in Arabidopsis. J Exp Bot.

[7] van Buer J, Cvetkovic J, Baier M (2016). Cold regulation of plastid ascorbate peroxidases serves as a priming hub controlling ROS signaling in Arabidopsis thaliana. BMC Plant Biol.

[8] Zhao MG, Chen L, Zhang LL, Zhang WH (2009). Nitric reductase dependent nitric oxide production is involved in cold acclimation and freezing tolerance in Arabidopsis. Plant Physiol.

[9] Puyaubert J, Baudouin E (2014). New clues for a cold case: nitric oxide response to low temperature. Plant Cell & Environ.

[10] Siddiqui MH, Al-Whaibi MH, Basalah MO (2011). Role of nitric oxide in tolerance of plants to abiotic stress. Protoplasma.

[11] Arasimowicz-Jelonek M, Floryszak-Wieczorek J (2014). Nitric oxide: an effective weapon of the plant or the pathogen?. Mol. Plant Pathol..

[12] Gupta KJ, Fernie AR, Kaiser WM, van Dongen JT (2011). On the origins of nitric oxide. Trends Plant Sci..

[13] Mur LA (2013). Nitric oxide in plants: an assessment of the current state of knowledge. AoB Plants.

[14] Thomas DD (2015). Breathing new life into nitric oxide signaling: A brief overview of the interplay between oxygen and nitric oxide. Redox Biol..

[15] Correa-Aragunde N, Foresi N, Lamattina L (2015). Nitric oxide is a ubiquitous signal for maintaining redox balance in plant cells: regulation of ascorbate peroxidase as a case study. J. Exp. Bot..

[16] Groβ F, Durner J, Gaupels F (2013). Nitric oxide, antioxidants and prooxidants in plant defence responses. Front. Plant Sci..

[17] Astier J, Lindermayr C (2012). Nitric oxide-dependent posttranslational modification in plants: an update. Int. J. Mol. Sci..

[18] Hess DT, Stamler JS (2012). Regulation by S-nitrosylation of protein post-translational modification. J. Biol. Chem..

[19] Guerra DD, Callis J (2012). Ubiquitin on the move: the ubiquitin modification system plays diverse roles in the regulation of endoplasmic reticulum- and plasma membrane-localized proteins. Plant Physiol..

[20] Cantrel C (2011). Nitric oxide participates in cold-responsive phosphosphingolipid formation and gene expression in *Arabidopsis thaliana*. New Phytol..

[21] Lozano-Juste J, León J (2010). Enhanced abscisic acid-mediated responses innia1,2noa1-2 triple mutant impaired in NIA/NR- and AtNOA1-dependent nitric oxide biosynthesis in Arabidopsis. Plant Physiol..

[22] Gibbs DJ (2014). Nitric oxide sensing in plants is mediated by proteolytic control of group VII ERF transcription factors. Mol. Cell.

[23] Lee BH, Henderson DA, Zhu JK (2005). The Arabidopsis cold-responsive transcriptome and its regulation by ICE1. Plant Cell.

[24] Kilian J (2007). The AtGenExpress global stress expression data set: protocols, evaluation and model data analysis of UV-B light, drought and cold stress responses. Plant J..

[25] Hu Y, Jiang L, Wang F, Yu D (2013). Jasmonate regulates the inducer of cbf expression-C-repeat binding factor/DRE binding factor1 cascade and freezing tolerance in Arabidopsis. Plant Cell.

[26] Lee HG, Seo PJ (2015). The MYB96-HHP module integrates cold and abscisic acid signaling to activate the CBF-COR pathway in Arabidopsis. Plant J..

[27] Kasukabe Y (2004). Overexpression of spermidine synthase enhances tolerance to multiple environmental stresses and up-regulates the expression of various stress-regulated genes in transgenic *Arabidopsis thaliana*. Plant & Cell Physiol.

[28] Korn M, Peterek S, Mock HP, Heyer AG, Hincha DK (2008). Heterosis in the freezing tolerance, and sugar and flavonoid contents of crosses between *Arabidopsis thaliana* accessions of widely varying freezing tolerance. Plant Cell & Environ..

[29] Guy C, Kaplan F, Kopka J, Selbig J, Hincha DK (2008). Metabolomics of temperature stress. Physiol. Plant..

[30] Berger S (2001). Enzymatic and non enzymatic lipid peroxidation in leaf development. Biochem. Biophys. Acta.

[31] Yoshida Y, Umeno A, Shichiri M (2013). Lipid peroxidation biomarkers for evaluating oxidative stress and assessing antioxidant capacity *in vivo*. J Clin. Biochem. Nutr..

[32] Catalá R (2014). The Arabidopsis 14-3-3 protein RARE COLD INDUCIBLE 1A links low-temperature response and ethylene biosynthesis to regulate freezing tolerance and cold acclimation. Plant Cell.

[33] Tähtiharju S, Palva T (2001). Antisense inhibition of protein phosphatase 2C accelerates cold acclimation in *Arabidopsis thaliana*. Plant J..

[34] Kawamura Y, Uemura M (2003). Mass spectrometric approach for identifying putative plasma membrane proteins of Arabidopsis leaves associated with cold acclimation. Plant J..

[35] Xin Z, Browse J (1998). Eskimo1 mutants of Arabidopsis are constitutively freezing-tolerant. Proc. Natl. Acad. Sci. USA.

[36] Nanjo T (1999). Antisense suppression of proline degradation improves tolerance to freezing and salinity in *Arabidopsis thaliana*. FEBS Lett..

[37] Zuther E, Schulz E, Childs LH, Hincha DK (2012). Clinal variation in the non-acclimated and cold-acclimated freezing tolerance of *Arabidopsis thaliana* accessions. Plant Cell & Environ..

[38] Alcázar R, García-Martínez JL, Cuevas JC, Tiburcio AF, Altabella T (2005). Overexpression of ADC2 in Arabidopsis induces dwarfism and late-flowering through GA deficiency. Plant J..

[39] Alet AI (2011). Putrescine accumulation in Arabidopsis thaliana transgenic lines enhances tolerance to dehydration and freezing stress. Plant Signal. & Behav..

[40] Nägele T, Stutz S, Hörmiller II, Heyer AG (2012). Identification of a metabolic bottleneck for cold acclimation in *Arabidopsis thaliana*. Plant J..

[41] Krol M (1995). Low-temperature stress and photoperiod affect an increased tolerance to photoinhibition in Pinus banksiana seedlings. Canadian Journal of Botany.

[42] Harvaux M, Kloppstech K (2001). The protective functions of carotenoid and flavonoid pigments against excess visible radiation at chilling temperature investigated in Arabidopsis *npq* and *tt* mutants. Planta.

[43] Schulz E, Tohge T, Zuther E, Fernie AR, Hincha DK (2016). Flavonoids are determinants of freezing tolerance and cold acclimation in Arabidopsis thaliana. Sci. Rep..

[44] Llorente F, Oliveros JC, Martínez-Zapater JM, Salinas J (2000). A freezing-sensitive mutant of Arabidopsis, *frs*1, is a new aba3 allele. Planta.

[45] Lozano-Juste, J., Colom-Moreno, R. & León, J. *In vivo* protein tyrosine nitration in *Arabidopsis thaliana*. *J. Exp. Bot*. **62**, 3501–3517. 10.1093/jxb/err042 PMC3130175 21378116

[46] Gill SS, Tuteja N (2010). Reactive oxygen species and antioxidant machinery in abiotic stress tolerance in crop plants. Plant Physiol. Biochem..

[47] Begara-Morales JC (2016). Antioxidant Systems are Regulated by Nitric Oxide-Mediated Post-translational Modifications (NO-PTMs). Front. Plant Sci..

[48] Castillo MC, León J (2008). Expression of the beta-oxidation gene 3-ketoacyl-CoA thiolase 2 (KAT2) is required for the timely onset of natural and dark-induced leaf senescence in Arabidopsis. J. Exp. Bot..

[49] Guo FQ, Okamoto M, Crawford NM (2003). Identification of a plant nitric oxide synthase gene involved in hormonal signaling. Science.

[50] Solfanelli C, Poggi A, Loreti E, Alpi A, Perata P (2006). Sucrose-specific induction of the anthocyanin biosynthetic pathway in Arabidopsis. Plant Physiol..

[51] Seo M, Jikumaru Y, Kamiya Y (2011). Profiling of Hormones and Related Metabolites in Seed Dormancy and Germination Studies. Meth. Mol. Biol..

