# Supplementary material for: Nitric Oxide Controls Constitutive Freezing Tolerance in Arabidopsis by Attenuating the Levels of Osmoprotectants, Stress-Related Hormones and Anthocyanins
Source: Sci Rep. 2018 Jun 18;8:9268. doi: 10.1038/s41598-018-27668-8 (PMC6006431; doi:10.1038/s41598-018-27668-8)
Supplement: Supplementary file 1 — Supplemental Information [file 41598_2018_27668_MOESM1_ESM.pdf]

Supplementary Information

Title: Nitric Oxide Controls Constitutive Freezing Tolerance in Arabidopsis by Attenuating the Levels of Osmoprotectants, Stress-Related Hormones and Anthocyanins

Authors: Álvaro Costa-Broseta[1], Carlos Perea-Resa[2§], Mari-Cruz Castillo[1], M. Fernanda Ruíz[2], Julio Salinas[2], José León[1*]

**Table S1. Genes that were up-regulated in non-acclimated *nia1,2noa1-2* plants and were reported to be related to cold-triggered responses.**				
Fold Change	FDR (LiMMA)	ProbeID	GI ID	Annotation
11,99	0,00006474	262128_at	At1g52690	LEA7__Late embryogenesis abundant protein (LEA) family protein
10,01	0,00000076	263486_at	At2g22200	AtERF056, DREB subfamily A-6 of ERF/AP2 transcription factor family
9,62	0,00001112	247047_at	At5g66650	Protein of unknown function (DUF607)
8,3	0,00001112	258347_at	At3g17520	Late embryogenesis abundant protein (LEA) family protein
7,72	0,00000449	255479_at	At4g02380	AtLEA5_SAG21__senescence-associated gene 21
7,61	0,00000108	254634_at	At4g18650	Protein DOG1-like 4 transcription factor-related
7,41	0,00002258	258498_at	At3g02480	Late embryogenesis abundant protein (LEA) family protein
6,48	0,00001112	253203_at	At4g34710	ADC2_ATADC2_SPE2__arginine decarboxylase 2
6,16	0,00002258	258349_at	At3g17609	HYH__HY5-homolog
5,1	0,00007264	264953_at	At1g77120	ADH_ADH1_ATADH_ATADH1__alcohol dehydrogenase 1
4,53	0,00596634	252131_at	At3g50930	BCS1__cytochrome BC1 synthesis
4,43	0,0006744	246099_at	At5g20230	ATBCB_BCB_BCB_SAG14__blue-copper-binding protein
4,24	0,00023704	253268_s_at	At4g34131	UGT73B3__UDP-glucosyl transferase 73B3
4,17	0,00010364	247280_at	At5g64260	EXL2__EXORDIUM like 2
4	0,00023961	252367_at	At3g48360	ATBT2_BT2__BTB and TAZ domain protein 2
3,94	0,0013199	262113_at	At1g02820	Late embryogenesis abundant 3 (LEA3) family protein
3,85	0,00007264	256818_at	At3g21420	LBO1__2-oxoglutarate (2OG) and Fe(II)-dependent oxygenase superfamily protein
3,83	0,00004115	260410_at	At1g69870	NRT1.7__nitrate transporter 1.7
3,83	0,00004038	259681_at	At1g77760	GNR1_NIA1_NR1__nitrate reductase 1
3,74	0,00041234	253104_at	At4g36010	Pathogenesis-related thaumatin superfamily protein
3,65	0,00001112	256891_at	At3g19030	unknown protein
3,57	0,00053704	266392_at	At2g41280	ATM10_M10__late embryogenesis abundant protein (M10) / LEA protein M10
3,56	0,00056934	259705_at	At1g77450	anac032_NAC032__NAC domain containing protein 32
3,48	0,00034439	260856_at	At1g21910	DREB26__Integrase-type DNA-binding superfamily protein
3,45	0,00017589	259365_at	At1g13300	HRS1__myb-like transcription factor family protein
3,45	0,00050337	255524_at	At4g02330	AtPME41_ATPMEPCRB_PME41__Plant invertase/pectin methylesterase inhibitor superfamily
3,33	0,00008324	254663_at	At4g18290	KAT2__potassium channel in Arabidopsis thaliana 2
3,3	0,01604548	249527_at	At5g38710	Methylenetetrahydrofolate reductase family protein
3,28	0,00099033	252123_at	At3g51240	F3'H_F3H_TT6__flavanone 3-hydroxylase
3,25	0,01709944	261648_at	At1g27730	STZ_ZAT10__salt tolerance zinc finger
3,08	0,00136951	250207_at	At5g13930	ATCHS_CHS_TT4__Chalcone and stilbene synthase family protein
3,06	0,00045948	263096_at	At2g16060	AHB1_ARATH GLB1_ATGLB1_GLB1_HB1_NSHB1__hemoglobin 1
3,06	0,00004871	262231_at	At1g68740	PHO1;H1__EXS (ERD1/XPR1/SYG1) family protein
3	0,00308858	254707_at	At4g18010	5PTASE2_AT5PTASE2_IP5PII__myo-inositol polyphosphate 5-phosphatase 2
2,99	0,00014127	262644_at	At1g62710	BETA-VPE_BETAVPE__beta vacuolar processing enzyme
2,96	0,00206871	248607_at	At5g49480	ATCP1_CP1__Ca2+-binding protein 1

2,86	0,00057889	247723_at	At5g59220	HAI1_SAG113__highly ABA-induced PP2C gene 1
2,84	0,01284149	266743_at	At2g02990	ATRNS1_RNS1__ribonuclease 1
2,81	0,00003968	255011_at	At4g10040	CYTC-2__cytochrome c-2
2,8	0,00035228	261192_at	At1g32870	ANAC013_ANAC13_NAC13__NAC domain protein 13
2,78	0,00129584	266313_at	At2g26980	CIPK3_SnRK3.17__CBL-interacting protein kinase 3
2,75	0,00450314	245628_at	At1g56650	ATMYB75_MYB75_PAP1_SIAA1__production of anthocyanin pigment 1
2,72	0,00055768	250648_at	At5g06760	AtLEA4-5_LEA4-5__Late Embryogenesis Abundant 4-5
2,72	0,00326299	247925_at	At5g57560	TCH4_XTH22__Xyloglucan endotransglucosylase/hydrolase family protein
2,67	0,01728287	255543_at	At4g01870	tolB protein-related
2,65	0,00054792	246125_at	At5g19875	unknown protein
2,64	0,00751802	252908_at	At4g39670	Glycolipid transfer protein (GLTP) family protein
2,63	0,00024078	266832_at	At2g30040	MAPKKK14__mitogen-activated protein kinase kinase kinase 14
2,6	0,00006604	262262_at	At1g70780	unknown protein
2,6	0,00133771	246310_at	At3g51895	AST12_SULTR3;1__sulfate transporter 3;1
2,56	0,00881697	256576_at	At3g28210	PMZ_SAP12__zinc finger (AN1-like) family protein
2,54	0,00928913	266544_at	At2g35300	AtLEA4-2_LEA18_LEA4-2__Late embryogenesis abundant protein, group 1 protein
2,53	0,00038051	264217_at	At1g60190	AtPUB19_PUB19__ARM repeat superfamily protein
2,51	0,0000475	267254_at	At2g23030	SNRK2-9_SNRK2.9__SNF1-related protein kinase 2.9
2,51	0,0003961	253559_at	At4g31140	O-Glycosyl hydrolases family 17 protein
2,5	0,01480833	264000_at	At2g22500	ATPUMP5_DIC1_UCP5__uncoupling protein 5
2,45	0,00072723	265649_at	At2g27510	ATFD3_FD3__ferredoxin 3
2,45	0,01481085	256442_at	At3g10930	unknown protein
2,44	0,02386155	261892_at	At1g80840	ATWRKY40_WRKY40__WRKY DNA-binding protein 40
2,42	0,00161733	265119_at	At1g62570	FMO GS-OX4__flavin-monooxygenase glucosinolate S-oxygenase 4
2,36	0,00409642	263823_s_at	At2g40340	AtERF48_DREB2C__Integrase-type DNA-binding superfamily protein
2,35	0,00376141	267246_at	At2g30250	ATWRKY25_WRKY25__WRKY DNA-binding protein 25
2,33	0,00002778	260357_at	At1g69260	AFP1__ABI5 binding protein
2,33	0,00009348	263473_at	At2g31750	UGT74D1__UDP-glucosyl transferase 74D1
2,32	0,00227904	251603_at	At3g57760	Protein kinase superfamily protein
2,31	0,00206124	255795_at	At2g33380	AtCLO3_CLO-3_CLO3_RD20__Caleosin-related family protein
2,31	0,00067342	248352_at	At5g52300	LTI65_RD29B__CAP160 protein
2,24	0,00071909	245523_at	At4g15910	ATDI21_DI21__drought-induced 21
2,21	0,00482667	248870_at	At5g46710	PLATZ transcription factor family protein
2,2	0,00663873	256017_at	At1g19180	JAZ1_TIFY10A__jasmonate-zim-domain protein 1
2,2	0,00706728	262050_at	At1g80130	Tetratricopeptide repeat (TPR)-like superfamily protein
2,2	0,00008759	253245_at	At4g34590	ATB2_AtbZIP11_BZIP11_GBF6__G-box binding factor 6
2,2	0,00313122	245711_at	At5g04340	C2H2_CZF2_ZAT6__zinc finger of Arabidopsis thaliana 6
2,2	0,00129607	248381_at	At5g51830	pfkB-like carbohydrate kinase family protein
2,18	0,00102094	265670_s_at	At2g32190	unknown protein
2,17	0,00022176	264436_at	At1g10370	ATGSTU17_ERD9_GST30_GST30B_GSTU17__Glutathione

				S-transferase family protein
2,17	0,00072928	253097_at	At4g37320	CYP81D5__cytochrome P450, family 81, subfamily D, polypeptide 5
2,12	0,00343103	266545_at	At2g35290	unknown protein
2,1	0,0101275	264758_at	At1g61340	F-box family protein
2,07	0,00372371	256296_at	At1g69480	EXS (ERD1/XPR1/SYG1) family protein
2,07	0,00030772	246495_at	At5g16200	50S ribosomal protein-related
2,05	0,00626975	254432_at	At4g20830	FAD-binding Berberine family protein
2,05	0,00057688	253841_at	At4g27830	BGLU10__beta glucosidase 10
2,04	0,00123699	251827_at	At3g55120	A11_CFI_TT5__Chalcone-flavanone isomerase family protein
2,04	0,00971052	267080_at	At2g41190	Transmembrane amino acid transporter family protein
2,04	0,00575895	258805_at	At3g04010	O-Glycosyl hydrolases family 17 protein
2,04	0,00551881	253485_at	At4g31800	ATWRKY18_WRKY18__WRKY DNA-binding protein 18
2,02	0,00872669	250777_at	At5g05440	PYL5_RCAR8__Polyketide cyclase/dehydrase and lipid transport superfamily protein

Table S2. Data, statistics, methods and box plots of the compared metabolomic analyses of Col-0 and *nia1,2noa1-2* plants.

Heat map of statistically significant biochemicals profiled in this study.

Shaded cells indicate p≤0.05 (red indicates that the mean values are significantly higher for that comparison; green values significantly lower). Blue-bolded text indicates 0.05<p<0.10.

PATHWAY SORT	SUPER PATHWAY	SUB PATHWAY	BIOCHEMICAL NAME	PLATFORM	COMP ID	Kegg	HMDB	Fold of Change: MUT / WT	Statistical Values, Welch's Two sample t-test, MUT / WT: P-VALUE	Statistical Values, Welch's Two sample t-test, MUT / WT: Q-VALUE	Mean Values: MUT	Mean Values: WT	% Filled Values: MUT	% Filled Values: WT	SUPER PATHWAY	SUB PATHWAY	CAS	RI	MASS
7			4-guanidinobutanoate	LC/MS pos	15681	C01035	HMDB03464	1,83	< 0.001	0,0000	1,320127	0,719600	100	100	Amino acid	Serine family (phosphoglycerate derived)	463-003;463-00-3;	1085	146,1
9			beta-hydroxypyruvate	GC/MS	15686	C00168	HMDB01352	2,01	< 0.001	0,0000	1,213150	0,603054	100	83	Amino acid	Serine family (phosphoglycerate derived)	3369-79-7;	1425,1	221
10			betaine	LC/MS pos	3141		HMDB00043	1,26	0,0017	0,0010	1,143688	0,907050	100	100	Amino acid	Serine family (phosphoglycerate derived)	107-43-7;	721	118,2
13		Serine family (phosphoglycerate derived)	cysteine	GC/MS	31453	C00097	HMDB00574	1,60	0,0012	0,0008	1,402775	0,875099	100	100	Amino acid	Serine family (phosphoglycerate derived)	52-90-4;56-89-3;	1560,1	218
17			glycine	GC/MS	11777	C00037	HMDB00123	1,80	< 0.001	0,0002	1,442788	0,802397	100	100	Amino acid	Serine family (phosphoglycerate derived)	56-40-6;	1166	101,9
26			N-acetylserine	GC/MS	37076		HMDB02931	3,61	< 0.001	0,0000	1,219933	0,337803	100	83	Amino acid	Serine family (phosphoglycerate derived)	97-14-3;	1526	218
28			O-acetylserine	GC/MS	15947	C00979	HMDB03011	3,87	< 0.001	0,0000	1,786816	0,462298	100	100	Amino acid	Serine family (phosphoglycerate derived)	66638-22-0;	1423	174
29			serine	GC/MS	1648	C00065	HMDB03406	2,40	< 0.001	0,0000	1,553095	0,648349	100	100	Amino acid	Serine family (phosphoglycerate derived)	56-45-1;	1389,1	204
40			4-hydroxycinnamate	LC/MS neg	36770	C00811	HMDB02035	0,59	0,0160	0,0069	0,761213	1,289534	100	100	Amino acid	Aromatic amino acid metabolism (PEP derived)	501-98-4;	1474	163
65			N-acetyltryptophan	LC/MS neg	33959	C03137		1,37	0,0036	0,0019	1,120230	0,817709	100	100	Amino acid	Aromatic amino acid metabolism (PEP derived)	1218-34-4;	2650	245,2
66			N-acetyltyrosine	LC/MS pos	32390		HMDB00866	0,70	0,0308	0,0117	0,823418	1,169464	100	100	Amino acid	Aromatic amino acid metabolism (PEP derived)	537-55-3 ;	2538	224,1
73		Aromatic amino acid metabolism (PEP derived)	phenylalanine	LC/MS pos	64	C00079	HMDB00159	1,17	0,1631	0,0469	1,076537	0,920159	100	100	Amino acid	Aromatic amino acid metabolism (PEP derived)	63-91-2;	2056	166,1
76			picolinate	GC/MS	1512	C10164	HMDB02243	0,96	0,7692	0,1568	1,021340	1,068260	83	100	Amino acid	Aromatic amino acid metabolism (PEP derived)	98-98-6;	1361,3	179,9
82			tryptophan	LC/MS pos	54	C00078	HMDB00929	1,31	0,0206	0,0084	1,160289	0,885292	100	100	Amino acid	Aromatic amino acid metabolism (PEP derived)	73-22-3;	2445	205,1
83			tyrosine	LC/MS pos	1299	C00082	HMDB00158	1,56	0,0013	0,0009	1,206917	0,774998	100	100	Amino acid	Aromatic amino acid metabolism (PEP derived)	60-18-4;	1516	182,1
89			alanine	GC/MS	1126	C00041	HMDB00161	0,91	0,1186	0,0363	0,958401	1,052456	100	100	Amino acid	Aspartate family (OAA derived)	56-41-7;	1147,6	115,9
91			asparagine	GC/MS	11398	C00152	HMDB00168	1,83	< 0.001	0,0001	1,446832	0,789741	100	100	Amino acid	Aspartate family (OAA derived)	70-47-3;	1651,2	231
92			aspartate	GC/MS	15996	C00049	HMDB00191	0,53	< 0.001	0,0000	0,693319	1,315592	100	100	Amino acid	Aspartate family (OAA derived)	56-84-8;	1529,7	232
93			beta-alanine	GC/MS	55	C00099	HMDB00056	1,90	< 0.001	0,0001	1,384022	0,727828	100	100	Amino acid	Aspartate family (OAA derived)	56-41-7;107-95-9;	1451,8	174
100			homoserine	GC/MS	23642	C00263,C02926	HMDB00719	1,05	0,6941	0,1462	1,118322	1,069916	100	100	Amino acid	Aspartate family (OAA derived)	672-15-1;	1471	218,1
103		Aspartate family (OAA derived)	lysine	GC/MS	22281	C00047	HMDB00182	0,82	0,1823	0,0508	0,895162	1,093570	100	100	Amino acid	Aspartate family (OAA derived)	56-87-1;	1679	156,1
104			methionine	LC/MS pos	1302	C00073	HMDB00696	1,35	0,0171	0,0073	1,139184	0,841047	100	100	Amino acid	Aspartate family (OAA derived)	63-68-3;	1252	150,1
104,5			methionine sulfoxide	LC/MS pos	18374	C02989	HMDB02005	1,05	0,7552	0,1547	1,046585	0,998038	100	100	Amino acid	Aspartate family (OAA derived)	3226-65-1;	729	166,1
111			N-acetylmethionine	LC/MS neg	1589	C02712	HMDB11745	1,68	0,0038	0,0020	1,209522	0,722086	100	83	Amino acid	Aspartate family (OAA derived)	65-82-7;	1805	190,1
120			pipecolate	GC/MS	1444	C00408	HMDB00070	0,69	0,0061	0,0030	0,756476	1,089221	100	100	Amino acid	Aspartate family (OAA derived)	4043-87-2;	1396,1	156
123	Amino acid		S-adenosylhomocysteine (SAH)	LC/MS pos	15948	C00021	HMDB00939	2,67	< 0.001	0,0000	1,606658	0,601460	100	100	Amino acid	Aspartate family (OAA derived)	979-92-0;	1480	385,1
126			threonine	LC/MS pos	1284	C00188	HMDB00167	1,55	< 0.001	0,0000	1,221281	0,789746	100	100	Amino acid	Aspartate family (OAA derived)	72-19-5;	713	120,1
129			2-aminobutyrate	GC/MS	1577	C02261	HMDB00650	0,97	0,8169	0,1614	0,946306	0,972271	100	100	Amino acid	Glutamate family (alpha-ketoglutarate derived)	1492-24-6;	1215,7	130

130		2-pyrrolidinone	GC/MS	31675		HMDB02039	0,79	0,0959	0,0306	0,851142	1,073212	100	100	Amino acid	Glutamate family (alpha-ketoglutarate derived)	616-45-5 ;	1190,9	142
132		4-acetamidobutanoate	LC/MS pos	1558	C02946	HMDB03681	1,11	0,5551	0,1247	1,102204	0,992157	83	100	Amino acid	Glutamate family (alpha-ketoglutarate derived)	3025-96-5;	1724	146,1
137		arginine	LC/MS pos	1638	C00062	HMDB00517	0,60	< 0.001	0,0006	0,800263	1,342596	100	100	Amino acid	Glutamate family (alpha-ketoglutarate derived)	1119-34-2;	650	175,2
141		citrulline	LC/MS pos	2132	C00327	HMDB00904	3,54	< 0.001	0,0000	1,635164	0,461449	100	100	Amino acid	Glutamate family (alpha-ketoglutarate derived)	372-75-8;	715	176,1
142		dimethylarginine (SDMA + ADMA)	LC/MS pos	36808	C03626	HMDB01539,HMDB03334	1,54	< 0.001	0,0003	1,233472	0,798432	100	100	Amino acid	Glutamate family (alpha-ketoglutarate derived)		812	203,2
144		gamma-aminobutyrate (GABA)	GC/MS	1416	C00334	HMDB00112	0,94	0,6006	0,1309	0,953709	1,011262	100	100	Amino acid	Glutamate family (alpha-ketoglutarate derived)	56-12-2;	1539,7	304,1
145		glutamate	GC/MS	57	C00025	HMDB03339	0,76	0,0033	0,0018	0,857759	1,134681	100	100	Amino acid	Glutamate family (alpha-ketoglutarate derived)	56-86-0;	1611,9	246
147		glutamine	LC/MS pos	53	C00064	HMDB00641	0,81	< 0.001	0,0000	0,913320	1,127656	100	100	Amino acid	Glutamate family (alpha-ketoglutarate derived)	56-85-9;	684	147,2
149	Glutamate family (alpha ketoglutarate derived)	histidine	GC/MS	37424	C00135	HMDB00177	6,18	< 0.001	0,0001	2,706123	0,437704	100	100	Amino acid	Glutamate family (alpha-ketoglutarate derived)	5934-29-2;	1837,3	254,1
154		N-acetylglutamate	LC/MS neg	15720	C00624	HMDB01138	0,74	0,0278	0,0108	0,831159	1,120176	100	100	Amino acid	Glutamate family (alpha-ketoglutarate derived)	5817-08-3;	630	188,1
155		N-acetylglutamine	LC/MS pos	33943	C02716	HMDB06029	3,48	< 0.001	0,0000	1,630905	0,468754	100	100	Amino acid	Glutamate family (alpha-ketoglutarate derived)	2490-97-3;	1204	189,1
157		N-acetylornithine	LC/MS pos	15630	C00437	HMDB03357	7,08	0,0015	0,0009	2,771063	0,391444	100	100	Amino acid	Glutamate family (alpha-ketoglutarate derived)	6205-08-9;	875	175,2
158		N-acetylproline	LC/MS pos	34387			0,20	< 0.001	0,0000	0,351146	1,733432	100	100	Amino acid	Glutamate family (alpha-ketoglutarate derived)	1074-79-9;	2184	158,1
159		N-acetylputrescine	LC/MS pos	37496	C02714	HMDB02064	2,23	< 0.001	0,0001	1,465423	0,657017	100	100	Amino acid	Glutamate family (alpha-ketoglutarate derived)	18233-70-0;	895	131,1
161		ornithine	GC/MS	1493	C00077	HMDB03374	0,77	0,1945	0,0525	0,866577	1,119058	100	100	Amino acid	Glutamate family (alpha-ketoglutarate derived)	3184-13-2;	1763,8	141,9
162		proline	LC/MS pos	1898	C00148	HMDB00162	0,96	0,4481	0,1072	0,970647	1,009807	100	100	Amino acid	Glutamate family (alpha-ketoglutarate derived)	147-85-3;	796	116,1
167		trans-4-hydroxyproline	GC/MS	1366	C01157	HMDB00725	0,72	< 0.001	0,0004	0,821372	1,148385	100	100	Amino acid	Glutamate family (alpha-ketoglutarate derived)	51-35-4;	1537	140
170,2		4-hydroxy-2-oxoglutaric acid	GC/MS	40062	C01127	HMDB02070	1,00	0,9208	0,1771	1,011329	1,011238	100	100	Amino acid	Glutamate family (alpha-ketoglutarate derived)	1187-99-1;	1658	333,1
170,6		cis-urocanate	LC/MS pos	40410			0,50	0,0583	0,0198	0,685648	1,381962	67	83	Amino acid	Glutamate family (alpha-ketoglutarate derived)	7699-35-6;	1398,6	139,1
171	Branched Chain Amino Acids (OAA derived)	isoleucine	LC/MS pos	1125	C00407	HMDB00172	0,96	0,6558	0,1409	1,034149	1,073870	100	100	Amino acid	Branched Chain Amino Acids (OAA derived)	73-32-5;	1614	132,1
172,5		2,3-dihydroxyisovalerate	GC/MS	38276	C04039		2,64	< 0.001	0,0000	0,983512	0,372689	100	33	Amino acid	Branched Chain Amino Acids (pyruvate derived)	1756-18-9;	1422	131
187	Branched Chain Amino Acids (pyruvate derived)	leucine	LC/MS pos	60	C00123	HMDB00687	0,99	0,8857	0,1710	1,024479	1,036620	100	100	Amino acid	Branched Chain Amino Acids (pyruvate derived)	61-90-5;	1674	132,2
196		valine	LC/MS pos	1649	C00183	HMDB00883	1,20	0,1412	0,0417	1,129917	0,944271	100	100	Amino acid	Branched Chain Amino Acids (pyruvate derived)	72-18-4;	1040	118,1
198		agmatine	GC/MS	15496	C00179	HMDB01432	29,81	< 0.001	0,0000	2,367465	0,079414	100	100	Amino acid	Amines and polyamines	2482-00-0;	1526	174
201	Amines and polyamines	putrescine	GC/MS	1408	C00134	HMDB01414	14,23	< 0.001	0,0000	1,994268	0,140136	100	100	Amino acid	Amines and polyamines	110-60-1;	1705,8	174
202		spermidine	LC/MS pos	485	C00315	HMDB01257	1,20	0,0850	0,0275	1,087023	0,904545	100	100	Amino acid	Amines and polyamines	124-20-9;	533	146,2
204		5-oxoproline	LC/MS pos	1494	C01879	HMDB00267	0,68	0,0144	0,0064	0,775916	1,137696	100	100	Amino acid	Glutathione metabolism	98-79-3;	1446	130,1
207	Glutathione metabolism	glutathione, oxidized (GSSG)	LC/MS pos	27727	C00127	HMDB03337	1,72	0,0026	0,0015	1,475482	0,857511	100	100	Amino acid	Glutathione metabolism	103239-24-3;	1535	613,1
208		glutathione, reduced (GSH)	LC/MS pos	2127	C00051	HMDB00125	1,12	0,6766	0,1439	1,047927	0,938114	100	100	Amino acid	Glutathione metabolism	70-18-8;	1274	308,1
212		1,3-dihydroxyacetone	GC/MS	35963	C00184	HMDB01882	1,42	0,0072	0,0035	1,177430	0,828970	100	100	Carbohydrate	Glycolysis	62147-49-3;	1263	103
214		1,6-anhydroglucose	GC/MS	21049		HMDB00640	2,05	0,0012	0,0008	1,082367	0,527513	100	17	Carbohydrate	Glycolysis	498-07-7;	1679,5	204,1
222		fructose-6-phosphate	GC/MS	12021	C05345	HMDB00124	1,73	< 0.001	0,0006	1,442362	0,836029	100	100	Carbohydrate	Glycolysis	103213-47-4;	1994,9	315,1
223	Glycolysis	glucose	GC/MS	20488	C00031	HMDB00122	1,92	< 0.001	0,0000	1,391516	0,726400	100	100	Carbohydrate	Glycolysis	50-99-7;	1866,8	217,1
224		glucose 1-phosphate	GC/MS	33755	C00103	HMDB01586	4,14	< 0.001	0,0000	1,926927	0,465400	100	100	Carbohydrate	Glycolysis	56401-20-8;	1853	217,1
225		glucose-6-phosphate (G6P)	GC/MS	31260	C00668	HMDB01401	1,97	< 0.001	0,0004	1,507981	0,765808	100	100	Carbohydrate	Glycolysis	103192-55-8;	2042,7	387,2
227		glycerate	GC/MS	1572	C00258	HMDB00139	0,82	0,0207	0,0084	0,894026	1,092746	100	100	Carbohydrate	Glycolysis	600-19-1;	1360,7	189
233		pyruvate	GC/MS	599	C00022	HMDB00243	1,06	0,5729	0,1261	1,041933	0,986253	100	83	Carbohydrate	Glycolysis	127-17-3;	1130,6	217
237		alpha-ketoglutarate	GC/MS	33453	C00026	HMDB00208	0,93	0,6856	0,1451	1,012586	1,093445	100	100	Carbohydrate	TCA cycle	305-72-6;328-50-7;22202-68-2;	1779	419,1
239		citrate	GC/MS	1564	C00158	HMDB00094	0,72	0,0181	0,0077	0,891469	1,235837	100	100	Carbohydrate	TCA cycle	77-92-9;	1763,4	273,1
240		fumarate	GC/MS	1643	C00122	HMDB00134	0,26	< 0.001	0,0000	0,449748	1,710826	100	100	Carbohydrate	TCA cycle	100-17-8;	1382,1	245
242	TCA cycle	malate	GC/MS	1303	C00149	HMDB00156	0,49	< 0.001	0,0000	0,657738	1,332090	100	100	Carbohydrate	TCA cycle	6915-15-7;	1502	233
243		maleate (cis-Butenedioate)	GC/MS	20676	C01384	HMDB00176	0,25	< 0.001	0,0001	0,462112	1,856868	100	100	Carbohydrate	TCA cycle	110-16-7;	1338	245
244		mesaconate (methylfumarate)	GC/MS	18493	C01732	HMDB00749	1,13	0,3099	0,0776	1,026306	0,908239	33	50	Carbohydrate	TCA cycle	498-24-8;	1429	184

246			succinate	GC/MS	1437	C00042	HMDB00254	2,78	< 0.001	0,0000	1,726322	0,621014	100	100	Carbohydrate	TCA cycle	110-15-6;	1348	247
258		Calvin cycle and pentose phosphate	ribose 5-phosphate	GC/MS	561	C00117	HMDB00618	1,24	0,1080	0,0340	1,184610	0,955558	100	100	Carbohydrate	Calvin cycle and pentose phosphate	18265-46-8;108321-05-7;	1928,4	315,1
278			arabonate	GC/MS	37516		HMDB00539	0,72	0,0121	0,0056	0,823072	1,135543	100	100	Carbohydrate	Amino sugar and nucleotide sugar	7643-75-7;	1736	292,1
280			erythritol	GC/MS	20699	C00503	HMDB02994	1,14	0,1187	0,0363	1,064228	0,934517	100	100	Carbohydrate	Amino sugar and nucleotide sugar	149-32-6;	1517,5	217
281			erythronate*	GC/MS	33477		HMDB00613	0,57	0,0093	0,0045	0,664077	1,160518	67	83	Carbohydrate	Amino sugar and nucleotide sugar	13752-84-6;	1546,9	292,1
282			fucose	GC/MS	15821	C00382	HMDB00174	0,66	0,0035	0,0019	0,875880	1,326174	100	100	Carbohydrate	Amino sugar and nucleotide sugar	2438-80-4;	1682,2	204
283			gluconate	GC/MS	587	C00257	HMDB00625	1,77	0,0022	0,0013	1,213177	0,686087	100	100	Carbohydrate	Amino sugar and nucleotide sugar	527-07-1;	1879,4	333
288		Amino sugar and nucleotide sugar	Isobar: ribulose 5-phosphate, xylulose 5-phosphate	GC/MS	37288			1,17	0,1941	0,0525	0,984878	0,844311	83	50	Carbohydrate	Amino sugar and nucleotide sugar		1935	357,2
300			ribitol	GC/MS	15772	C00474	HMDB00508	0,81	0,2609	0,0668	0,863797	1,061328	100	67	Carbohydrate	Amino sugar and nucleotide sugar	488-81-3;	1692,4	217
302	Carbohydrate		ribose	GC/MS	12080	C00121	HMDB00283	0,92	0,2054	0,0545	0,927497	1,003406	100	100	Carbohydrate	Amino sugar and nucleotide sugar	50-69-1;	1639,2	204
303			ribulose	GC/MS	35855	C00309	HMDB00621,HMDB03371	1,14	0,4848	0,1136	0,840746	0,735943	50	50	Carbohydrate	Amino sugar and nucleotide sugar	488-84-6;	1662	306,1
304			threitol	GC/MS	35854	C16884	HMDB04136	1,32	0,0435	0,0161	1,259577	0,953033	100	100	Carbohydrate	Amino sugar and nucleotide sugar	2418-52-2;	1513	217,1
311			xylonate	GC/MS	35638	C00502,C05411		0,73	< 0.001	0,0007	0,853602	1,170509	100	100	Carbohydrate	Amino sugar and nucleotide sugar	73686-31-7;	1722	292
312			xylose	GC/MS	15835	C00181	HMDB00098	1,49	0,0049	0,0025	1,397658	0,940063	100	100	Carbohydrate	Amino sugar and nucleotide sugar	609-06-3;	1723,9	204
316			inositol 1-phosphate (I1P)	GC/MS	1481		HMDB00213	1,37	0,0096	0,0046	1,199858	0,873870	100	100	Carbohydrate	Inositol metabolism	106032-59-1;	2057,8	318,1
317		Inositol metabolism	inositol 2-phosphate (I2P)	GC/MS	27725	C01177	HMDB01313,HMDB02985,HMDB00213	1,15	0,5063	0,1176	1,090818	0,949002	100	100	Carbohydrate	Inositol metabolism		2072,9	318,1
319			myo-inositol	GC/MS	19934	C00137	HMDB00211	1,89	< 0.001	0,0001	1,502101	0,794178	100	100	Carbohydrate	Inositol metabolism	87-89-8;	1924,9	217
329			3-deoxyoctulosonate	GC/MS	15942			1,02	0,9908	0,1889	0,969085	0,952525	67	83	Carbohydrate	Sucrose, glucose, fructose metabolism	103404-70-2;	1942	373,2
341			fructose	GC/MS	577	C00095	HMDB00660	1,69	< 0.001	0,0000	1,340786	0,794868	100	100	Carbohydrate	Sucrose, glucose, fructose metabolism	57-48-7;	1758	217
342			galactinol	GC/MS	34706	C01235	HMDB05826	1,86	0,0040	0,0021	1,404294	0,753303	100	83	Carbohydrate	Sucrose, glucose, fructose metabolism	16908-86-4;	2253	204
363			maltose	GC/MS	15804	C00208	HMDB00163	10,54	< 0.001	0,0000	2,682968	0,254605	100	100	Carbohydrate	Sucrose, glucose, fructose metabolism	6363-53-7;	2176,2	204,1
366		Sucrose, glucose, fructose metabolism	maltotriose	GC/MS	27723	C01835	HMDB01262	2,22	0,0021	0,0013	1,676808	0,755790	100	100	Carbohydrate	Sucrose, glucose, fructose metabolism	1109-28-0;	2368,3	361,2
372			mannose-6-phosphate	GC/MS	1469	C00275	HMDB01078	1,76	0,0012	0,0008	1,460114	0,827473	100	100	Carbohydrate	Sucrose, glucose, fructose metabolism	70442-25-0;104872-94-8;	2004,6	387,1
378			raffinose	LC/MS neg	586	C00492	HMDB03213	1,70	< 0.001	0,0002	1,392459	0,818280	100	100	Carbohydrate	Sucrose, glucose, fructose metabolism	17629-30-0;	997	503,2
385			sucrose	LC/MS neg	1519	C00089	HMDB00258	17,66	< 0.001	0,0000	2,167713	0,122763	100	100	Carbohydrate	Sucrose, glucose, fructose metabolism	57-50-1;	875	341,2
388			trehalose	GC/MS	15573	C01083	HMDB00975	3,77	0,0456	0,0165	3,467078	0,920220	67	100	Carbohydrate	Sucrose, glucose, fructose metabolism	6138-23-4;	2174,9	361,2
391,3			Isobar: 1-kestose, levan	LC/MS neg	40806			3,38	< 0.001	0,0000	1,066474	0,315510	100	50	Carbohydrate	Sucrose, glucose, fructose metabolism		1062	503,2
394		C5 branched dibasic acid metabolism	citramalate	GC/MS	22158	C02612,C00815	HMDB00426	0,90	0,5260	0,1213	0,760330	0,846623	50	67	Carbohydrate	C5 branched dibasic acid metabolism	6236-10-8;	1490,7	247
404			2-hydroxymyristate	LC/MS neg	32413	C13790	HMDB02261	0,89	0,5649	0,1250	0,916473	1,028995	100	100	Lipids	Free fatty acid	2507-55-3 ;	5345	243,3
405			2-hydroxypalmitate	LC/MS neg	35675			0,88	0,3633	0,0899	0,923528	1,046754	100	100	Lipids	Free fatty acid	764-67-0;	5508	271,3
406			2-hydroxystearate	LC/MS neg	17945	C03045		0,56	< 0.001	0,0002	0,766443	1,380543	100	100	Lipids	Free fatty acid	629-22-1;	5705	299,4
437			azelate (nonanedioate)	LC/MS neg	18362	C08261	HMDB00784	1,18	0,1699	0,0483	1,141886	0,970580	100	100	Lipids	Free fatty acid	123-99-9;	1322	187,2
441			caproate (6:0)	LC/MS neg	32489	C01585	HMDB00535	1,56	0,0130	0,0059	1,318963	0,848142	100	100	Lipids	Free fatty acid	142-62-1;	2766	115,2
466,1		Free fatty acid	2-ethylhexanoate (isobar with 2-propylpentanoate)	LC/MS neg	35490			0,79	0,0046	0,0024	0,933054	1,176513	100	100	Lipids	Free fatty acid		3909	143,1
472			linoleate (18:2n6)	LC/MS neg	1105	C01595	HMDB00673	1,07	0,8173	0,1614	1,143755	1,064021	100	100	Lipids	Free fatty acid	60-33-3;	5533	279,3
473			linolenate [alpha or gamma; (18:3n3 or 6)]	LC/MS neg	34035	C06427	HMDB01388	0,82	0,2052	0,0545	1,081728	1,322116	100	100	Lipids	Free fatty acid		5450	277,3
474			malonate (propanedioate)	LC/MS neg	15872	C00383	HMDB00691	1,15	< 0.001	0,0006	1,062183	0,921313	100	100	Lipids	Free fatty acid	141-82-2;26522-22-85-0;	606	103,2
492			palmitoleate (16:1n7)	LC/MS neg	33447	C08362	HMDB03229	0,98	0,5600	0,1250	0,996675	1,020105	100	100	Lipids	Free fatty acid	373-49-9;	5477	253,3
510		Oxylipins	13-HODE + 9-HODE	LC/MS neg	37752			2,46	< 0.001	0,0000	1,384845	0,562820	100	100	Lipids	Oxylipins		5247	295,2
510,5	Lipids		13-HOTE (6Z,9Z,11E or 9Z,11E,15Z)	LC/MS pos	39688			1,51	0,0657	0,0218	1,253240	0,829157	100	100	Lipids	Oxylipins		5374	277,3

523,4		Glycerolipids	1,2-propanediol	GC/MS	38002	C00717,C02912,C00583,C01506,C02917	HMDB01881	1,01	0,8173	0,1614	1,383928	1,370142	100	100	Lipids	Glycerolipids	57-55-6;	1041	117
532			1-palmitoylglycerol (1-monopalmitin)	GC/MS	21127			1,17	0,2843	0,0724	1,064791	0,912842	100	100	Lipids	Glycerolipids	542-44-9;	2119,5	371,3
557		Phospholipids	1-palmitoylglycerophosphocholine	LC/MS pos	33955			0,94	0,7989	0,1599	0,941610	1,002924	100	100	Lipids	Phospholipids	17364-16-8;	5671	496,4
572			glycerol 3-phosphate (G3P)	GC/MS	15365	C00093	HMDB00126	**0,88**	0,0568	0,0195	0,925191	1,054324	100	100	Lipids	Phospholipids	29849-82-9;	1719,7	357,1
574			glycerophosphorylcholine (GPC)	LC/MS pos	15990	C00670	HMDB00086	1,03	0,5311	0,1218	1,025120	0,997513	100	100	Lipids	Phospholipids	28319-77-9;	694	258,1
575			phosphoethanolamine	GC/MS	12102	C00346	HMDB00224	1,28	0,2226	0,0583	1,134484	0,887739	100	100	Lipids	Phospholipids	1071-23-4;	1577,3	299,1
579		Choline metabolism	choline phosphate	LC/MS pos	34396			1,94	< 0.001	0,0000	1,489454	0,767694	100	100	Lipids	Choline metabolism	72556-74-2;	695	184,1
580			ethanolamine	GC/MS	1497	C00189	HMDB00149	1,33	0,0271	0,0107	1,156968	0,869888	100	100	Lipids	Choline metabolism	141-43-5;	1304	174,1
601		Sterols	campesterol	GC/MS	33997	C01789	HMDB02869	0,86	0,0377	0,0142	0,930432	1,078081	100	100	Lipids	Sterols	474-62-4;	2353	129
618			squalene	GC/MS	1518	C00751	HMDB00256	1,04	0,8654	0,1684	0,943987	0,905518	67	100	Lipids	Sterols	111-02-4;	2205	69
643		CoA metabolism	pantothenate	LC/MS pos	1508	C00864	HMDB00210	0,68	< 0.001	0,0002	0,810298	1,187954	100	100	Cofactors, Prosthetic Groups, Electron Carriers	CoA metabolism	137-08-6;	2218	220,1
654			nicotinamide adenine dinucleotide (NAD+)	LC/MS pos	5278	C00003	HMDB00902	1,02	0,9501	0,1819	0,991131	0,972411	100	100	Cofactors, Prosthetic Groups, Electron Carriers	Nicotinate and nicotinamide metabolism	53-84-9;	1370	664
658			nicotinamide ribonucleotide (NMN)	LC/MS pos	22152	C00455	HMDB00229	1,47	< 0.001	0,0000	1,237488	0,841264	100	100	Cofactors, Prosthetic Groups, Electron Carriers	Nicotinate and nicotinamide metabolism	1094-61-7;	886	335,1
659		Nicotinate and nicotinamide metabolism	nicotinamide riboside*	LC/MS pos	33013			1,10	0,1782	0,0500	1,076931	0,974718	100	100	Cofactors, Prosthetic Groups, Electron Carriers	Nicotinate and nicotinamide metabolism	1341-23-7;	955	255,1
660			nicotinate	LC/MS pos	1504	C00253	HMDB01488	2,59	0,0031	0,0017	1,884651	0,727396	100	100	Cofactors, Prosthetic Groups, Electron Carriers	Nicotinate and nicotinamide metabolism	59-67-6;	1241	124,1
663			nicotinate ribonucleoside*	LC/MS pos	33471			0,64	< 0.001	0,0002	0,760465	1,186418	100	100	Cofactors, Prosthetic Groups, Electron Carriers	Nicotinate and nicotinamide metabolism	2625-49-2;	1105	256
677		Oxidative phosphorylation	methylphosphate	GC/MS	37070			0,91	0,2183	0,0576	0,946478	1,040068	100	100	Cofactors, Prosthetic Groups, Electron Carriers	Oxidative phosphorylation	7023-27-0;	1221	240,9
678			phosphate	GC/MS	11438	C00009	HMDB01429	**0,88**	0,0506	0,0180	0,920964	1,043163	100	100	Cofactors, Prosthetic Groups, Electron Carriers	Oxidative phosphorylation	7664-38-2 ;	1307,7	298,9
685		Riboflavin and FAD metabolism	riboflavin (Vitamin B2)	LC/MS pos	1827	C00255	HMDB00244	**0,87**	0,0968	0,0307	0,983983	1,128384	100	100	Cofactors, Prosthetic Groups, Electron Carriers	Riboflavin and FAD metabolism	83-88-5;	3111	377,2
689	Cofactors, Prosthetic Groups, Electron Carriers	Quinone metabolism	phytonadione (Vitamin K1)	GC/MS	12250	C02059	HMDB03555	**0,88**	0,0731	0,0239	0,880335	0,997263	33	83	Cofactors, Prosthetic Groups, Electron Carriers	Quinone metabolism	84-80-0;	2407	596,5
692			ascorbate (Vitamin C)	GC/MS	1640	C00072	HMDB00044	1,75	< 0.001	0,0003	1,362269	0,779938	100	100	Cofactors, Prosthetic Groups, Electron Carriers	Ascorbate metabolism	134-03-2;	1850,1	332,1

693		Ascorbate metabolism	dehydroascorbate	GC/MS	1659	C05422	HMDB01264	1,16		0,4723	0,1114		1,161155	1,003702		100	100		Cofactors, Prosthetic Groups, Electron Carriers	Ascorbate metabolism	490-83-5;	1800	245,1
696			glucarate (saccharate)	GC/MS	1476	C00818	HMDB00663	1,37		0,0267	0,0106		1,148350	0,838008		100	83		Cofactors, Prosthetic Groups, Electron Carriers	Ascorbate metabolism	5793-88-4;	1885,8	333,1
700			threonate	GC/MS	27738	C01620	HMDB00943	0,96		0,7111	0,1484		0,984652	1,020522		100	100		Cofactors, Prosthetic Groups, Electron Carriers	Ascorbate metabolism	70753-61-6;	1560,7	292,1
707		Tocopherol metabolism	alpha-tocopherol	GC/MS	1561	C02477	HMDB01893	**2,22**		0,0640	0,0214		1,381879	0,621818		67	50		Cofactors, Prosthetic Groups, Electron Carriers	Tocopherol metabolism	59-02-9;10191-41-0;	2305,4	502,5
716		Vitamin B metabolism (B6 or B12)	pyridoxal	LC/MS pos	1651	C00250	HMDB01545	**1,22**		0,0551	0,0191		1,110228	0,911474		100	100		Cofactors, Prosthetic Groups, Electron Carriers	Vitamin B metabolism (B6 or B12)	65-22-5;	1210	168,1
720			pyridoxate	LC/MS neg	31555	C00847	HMDB00017	0,65		< 0.001	0,0001		0,802280	1,229241		100	100		Cofactors, Prosthetic Groups, Electron Carriers	Vitamin B metabolism (B6 or B12)	82-82-6 ;	2210	182,1
727		Chlorophyll and heme metabolism	pheophorbide A	LC/MS pos	35879	C18021		3,00		0,0443	0,0161		2,158170	0,720364		100	100		Cofactors, Prosthetic Groups, Electron Carriers	Chlorophyll and heme metabolism	15664-29-6;	6053	593,4
729,5			phytol	GC/MS	40140	C01389	HMDB02019	1,10		0,1626	0,0469		1,070211	0,970793		100	100		Cofactors, Prosthetic Groups, Electron Carriers	Chlorophyll and heme metabolism	150-86-7;	1964,7	143,1
737	Nucleotide	Purine metabolism	2'-deoxyadenosine	LC/MS pos	1553	C00559	HMDB00101	1,57		< 0.001	0,0006		1,362312	0,870353		100	100		Nucleotide	Purine metabolism	16373-93-6;	1632	252,1
747			adenine	GC/MS	554	C00147	HMDB00034	0,98		0,7191	0,1494		0,979290	0,995808		100	100		Nucleotide	Purine metabolism	73-24-5;	1804,2	264
748			adenosine	LC/MS pos	555	C00212	HMDB00050	8,36		< 0.001	0,0000		2,095329	0,250561		100	100		Nucleotide	Purine metabolism	58-61-7;	1650	268,1
749			adenosine 2'-monophosphate (2'-AMP)	LC/MS neg	36815	C00946	HMDB11617	0,15		0,1316	0,0397		0,830253	5,527646		100	100		Nucleotide	Purine metabolism	130-49-4;	1497	346,1
752			adenosine 3'-monophosphate (3'-AMP)	LC/MS pos	35142	C01367	HMDB03540	1,23		0,3100	0,0776		1,031393	0,840552		100	100		Nucleotide	Purine metabolism	84-21-9;	1451	348,1
754			adenosine 5'-monophosphate (AMP)	LC/MS pos	32342	C00020	HMDB00045	4,38		< 0.001	0,0001		2,576838	0,588330		100	100		Nucleotide	Purine metabolism	149022-20-8;	1210	348,1
756			adenosine-2',3'-cyclic monophosphate	LC/MS pos	37467	C02353		1,10		0,4732	0,1114		0,980307	0,892313		100	100		Nucleotide	Purine metabolism	37063-35-7;	1484	330,2
756,5			N6-carbamoylthreonyladenosine	LC/MS pos	35157			1,20		0,2343	0,0611		1,051697	0,872792		100	100		Nucleotide	Purine metabolism	24719-82-2;	2656	413
759			allantoin	GC/MS	22808	C02350	HMDB00462	0,44		< 0.001	0,0000		0,640984	1,458941		100	100		Nucleotide	Purine metabolism	97-59-6;	1541,3	314,1
762			guanine	LC/MS pos	32352	C00242	HMDB00132	0,94		0,5400	0,1226		0,976665	1,033691		100	100		Nucleotide	Purine metabolism	73-40-5;	1022	152,1
763			guanosine	LC/MS pos	1573	C00387	HMDB00133	2,58		< 0.001	0,0000		1,364483	0,528274		100	100		Nucleotide	Purine metabolism	118-00-3;	1676	284
768			guanosine-2',3'-cyclic monophosphate	LC/MS neg	37139	C06194	HMDB11629	1,15		0,3930	0,0961		1,005240	0,874867		100	100		Nucleotide	Purine metabolism		1454	343,9
771			inosine	LC/MS neg	1123			1,17		0,1886	0,0522		0,945119	0,805509		100	50		Nucleotide	Purine metabolism	58-63-9;	1630	267,2
773			N1-methyladenosine	LC/MS pos	15650	C02494	HMDB03331	2,33		0,0012	0,0008		1,902219	0,817613		100	100		Nucleotide	Purine metabolism	15763-06-1;	1356	282,1
780			urate	GC/MS	1604	C00366	HMDB00289	0,69		0,0063	0,0031		0,782895	1,129808		100	100		Nucleotide	Purine metabolism	69-93-2;120K5305;	1928	441,2
781			xanthine	GC/MS	3147	C00385	HMDB00292	0,60		0,4288	0,1032		0,994130	1,652761		67	83		Nucleotide	Purine metabolism	69-89-6;	1889,9	353
784,1			guanosine 3'-monophosphate (3'-GMP)	LC/MS pos	39786			1,22		0,2942	0,0745		1,075376	0,880209		83	83		Nucleotide	Purine metabolism		1516,8	364
802		Pyrimidine metabolism	cytidine	LC/MS pos	514	C00475	HMDB00089	0,70		< 0.001	0,0006		0,765470	1,095818		100	100		Nucleotide	Pyrimidine metabolism	65-46-3;	1065	244
806			cytidine-3'-monophosphate (3'-CMP)	LC/MS pos	2959	C05822		0,76		0,2029	0,0545		0,916031	1,198574		33	83		Nucleotide	Pyrimidine metabolism	84-52-6;	1181	323,9
808			cytosine-2',3'-cyclic monophosphate	LC/MS neg	37465	C02354	HMDB11691	0,96		0,6488	0,1407		0,989577	1,035745		100	100		Nucleotide	Pyrimidine metabolism	15718-51-1;	957	304,1
815			pseudouridine	LC/MS neg	33442	C02067	HMDB00767	0,90		0,5403	0,1226		0,898776	0,994797		100	83		Nucleotide	Pyrimidine metabolism	1445-07-4;	1104	243,1
822			uracil	GC/MS	605	C00106	HMDB00300	**0,54**		0,0585	0,0198		0,835847	1,543827		83	67		Nucleotide	Pyrimidine metabolism	66-22-8;	1370,4	241

823			uridine	LC/MS neg	606	C00299	HMDB00296	1,53		< 0.001	0,0000		1,249834	0,816987		100	100		Nucleotide	Pyrimidine metabolism	58-96-8;	1467	243,1
827			uridine-2',3'-cyclic monophosphate	LC/MS neg	37137	C02355	HMDB11640	1,70		< 0.001	0,0001		1,402071	0,824766		100	100		Nucleotide	Pyrimidine metabolism		1082	304,9
921		Amine derived	tyramine	LC/MS pos	1603	C00483	HMDB00306	4,38		< 0.001	0,0000		1,778031	0,406389		100	100		Secondary metabolism	Amine derived	60-19-5;	1503	138,1
926		Benzenoids	3,4-dihydroxybenzoate	GC/MS	18292	C00230	HMDB01856	1,09		0,5487	0,1239		0,950420	0,872170		50	50		Secondary metabolism	Benzenoids	99-50-3;	1768	193
940			benzoyl-O-glucose	LC/MS pos	38302			5,26		< 0.001	0,0000		1,754506	0,333832		100	100		Secondary metabolism	Benzenoids		3026	302
952		Fatty acid and sugar derivatives	galactarate (mucic acid)	GC/MS	20881	C00879	HMDB00639	1,58		0,0119	0,0056		1,477286	0,934989		100	100		Secondary metabolism	Fatty acid and sugar derivatives	526-99-8;	1899,3	333,1
970			dihydrokaempferol	LC/MS neg	37116	C00974		6,05		< 0.001	0,0001		0,899987	0,148846		100	17		Secondary metabolism	Flavonoids	480-20-6;	2568	287
987		Flavonoids	kaempferol 3-O-beta-glucoside	LC/MS neg	38145			1,16		0,1140	0,0354		1,038875	0,898199		100	100		Secondary metabolism	Flavonoids	480-10-4;	3085	447,2
993			naringenin	LC/MS neg	21182	C00509	HMDB02670	1,86		0,0046	0,0024		1,173609	0,629594		100	33		Secondary metabolism	Flavonoids	67604-48-2;	3484	271,1
1005,4	Secondary metabolism		kaempferol-3-rhamnoside	LC/MS pos	40041			1,14		0,1596	0,0465		1,097377	0,962751		100	100		Secondary metabolism	Flavonoids	482-39-3;	4160,4	432,8
1006,1			sulforaphane	LC/MS pos	38697		HMDB05792	1,40		0,0132	0,0060		1,172377	0,838351		100	100		Secondary metabolism	Glucosinolate	4478-93-7;	3334,3	178,1
1006,3		Glucosinolate	3-methylsulfinylpropyl isothiocyanate	LC/MS pos	39585			1,34		0,0443	0,0161		1,144110	0,855548		100	100		Secondary metabolism	Glucosinolate	505-44-2;	2833,4	164,1
1006,5			sulforaphane-glutathione	LC/MS pos	40422			1,47		0,0296	0,0113		1,142644	0,778013		100	100		Secondary metabolism	Glucosinolate	289711-21-3;	2359,9	485,2
1020			5-formylsalicylate	LC/MS neg	22246			**1,28**		0,0683	0,0224		1,145655	0,894883		100	100		Secondary metabolism	Phenylpropanoids	616-76-2;	2470	165
1030		Phenylpropanoids	ferulate	GC/MS	35630	C01494	HMDB00954	1,19		0,3826	0,0941		1,127732	0,945111		83	33		Secondary metabolism	Phenylpropanoids	537-98-4;	1929	338,1
1038			sinapate	LC/MS neg	21150	C00482		12,22		< 0.001	0,0000		2,161746	0,176951		100	83		Secondary metabolism	Phenylpropanoids	530-59-6;	1986	223,1
1041			syringaldehyde	LC/MS neg	37449			3,04		< 0.001	0,0000		1,642062	0,539453		100	100		Secondary metabolism	Phenylpropanoids	134-96-3;	2500	181,1
1096	Xenobiotics	Chemicals	5-hydroxymethylfurfural	LC/MS pos	37429	C11101		1,33		0,0283	0,0109		1,189592	0,891746		100	100		Xenobiotics	Chemicals	67-47-0;	1992	127,1
1151			trizma acetate	GC/MS	20710	C07182		0,56		0,1927	0,0525		0,782507	1,404504		100	83		Xenobiotics	Chemicals	6850-28-8;	1525	306,2

from Hamilton Company. Recovery standards were added prior to the first step in the extraction process for quality Control (QC) purposes. Sample preparation was conducted by series of organic and aqueous extractions to remove the protein fraction while allowing maximum recovery of small molecules. The resulting extract was divided into two fractions; one for analysis by Liquid Chromatography (LC) and one for analysis by Gas Chromatography (GC). Samples were placed briefly on a TurboVap® (Zymark) to remove the organic solvent. Each sample was then frozen, dried under vacuum and prepared for either LC/MS or GC/MS.

The LC/MS portion of the platform was based on a Waters ACQUITY UPLC and a Thermo-Finnigan LTQ mass spectrometer, which consisted of an electrospray ionization (ESI) source and linear ion-trap (LIT) mass analyzer. The sample extract was split into two aliquots, dried, then reconstituted in acidic or basic LC-compatible solvents, each of which contained 11 or more injection standards at fixed concentrations. One aliquot was analyzed using acidic positive ion optimized conditions and the other using basic negative ion optimized conditions in two independent injections using separate dedicated columns. Extracts reconstituted in acidic conditions were gradient eluted using water and methanol both containing 0.1% Formic acid, while the basic extracts, which also used water/methanol, contained 6.5 mM ammonium bicarbonate. The MS analysis alternated between MS and data-dependent MS2 scans using dynamic exclusion. The Thermo-Finnigan LTQ-FT mass spectrometer had a linear ion-trap (LIT) front end and a Fourier transform ion cyclotron resonance (FT-ICR) mass spectrometer back end. For ions with counts greater than 2 million, an accurate mass measurement could be performed. Accurate mass measurements could be made on the parent ion as well as fragments. The typical mass error was less than 5 ppm. Ions with less than two million counts require fragmentation spectra (MS/MS) typically generated in data dependent manner or targeted MS/MS in the case of lower level signals .

The samples destined for GC/MS analysis were re-dried under vacuum desiccation for a minimum

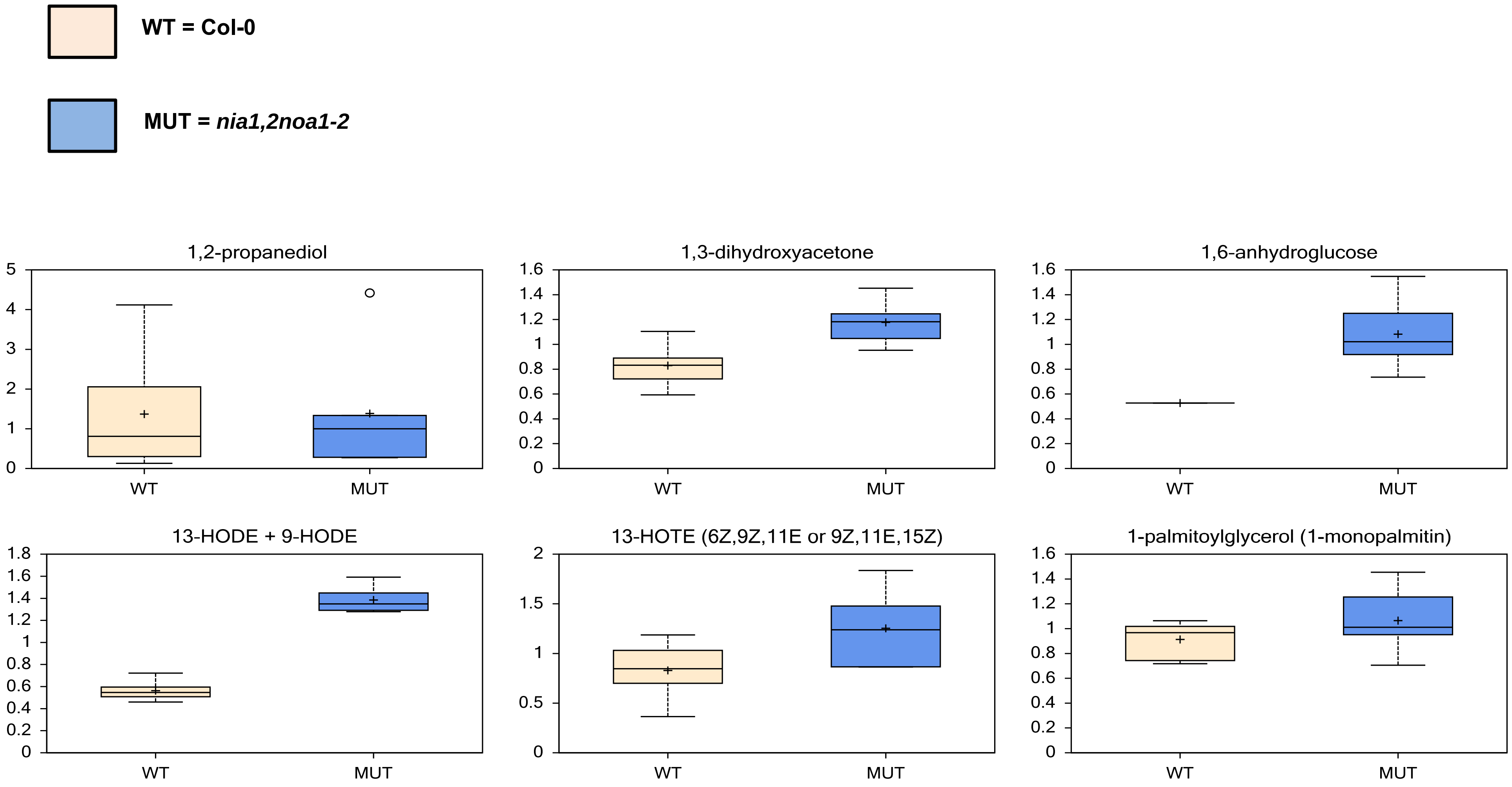
WT = Col-0
MUT = nia1,2noa1-2
1,2-propanediol
1,3-dihydroxyacetone
1,6-anhydroglucose
13-HODE + 9-HODE
13-HOTE (6Z,9Z,11E or 9Z,11E,15Z)
1-palmitoylglycerol (1-monopalmitin)
WT
MUT

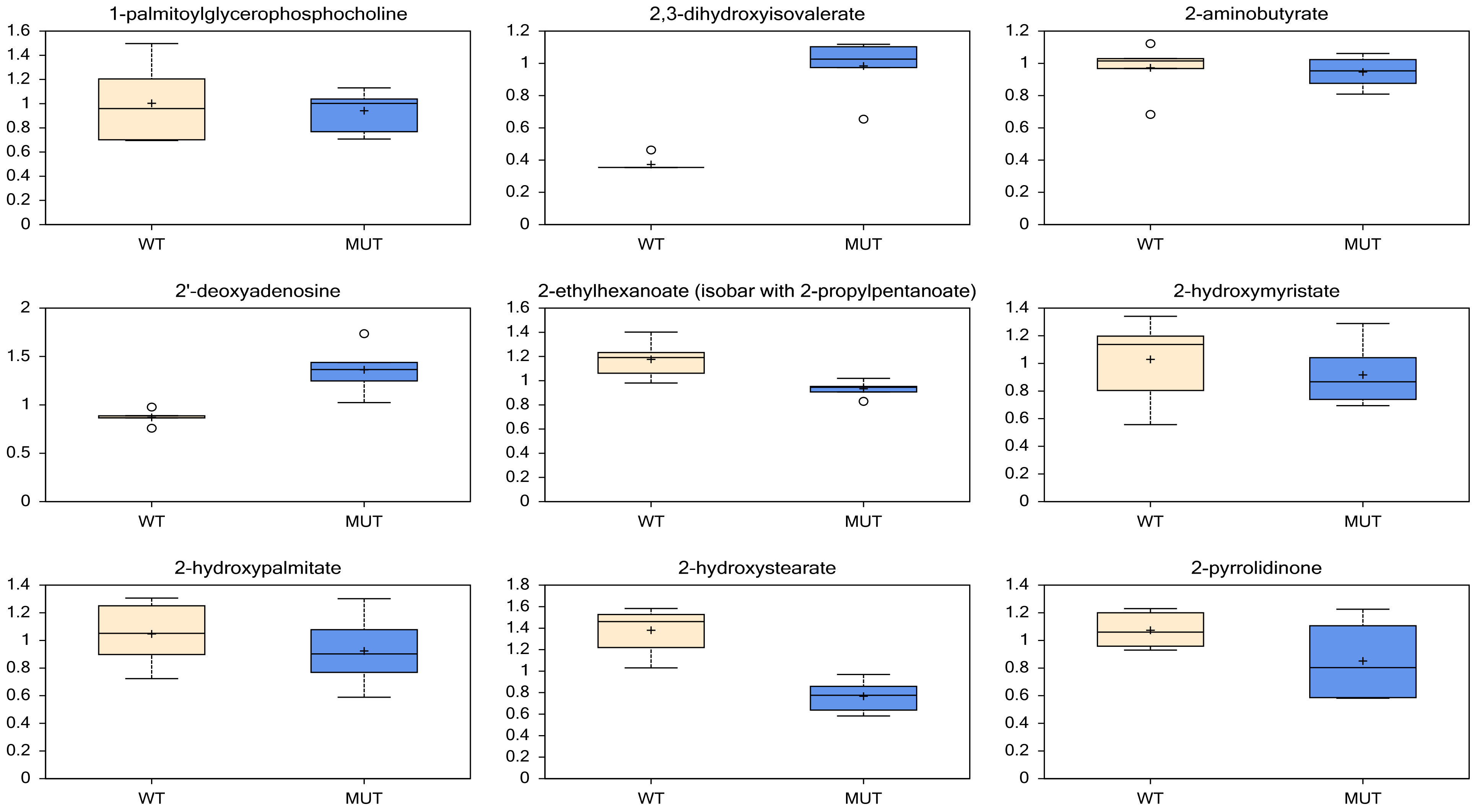
1-palmitoylglycerophosphocholine
2,3-dihydroxyisovalerate
2-aminobutyrate
2'-deoxyadenosine
2-ethylhexanoate (isobar with 2-propylpentanoate)
2-hydroxymyristate
2-hydroxypalmitate
2-hydroxystearate
2-pyrrolidinone
WT
MUT

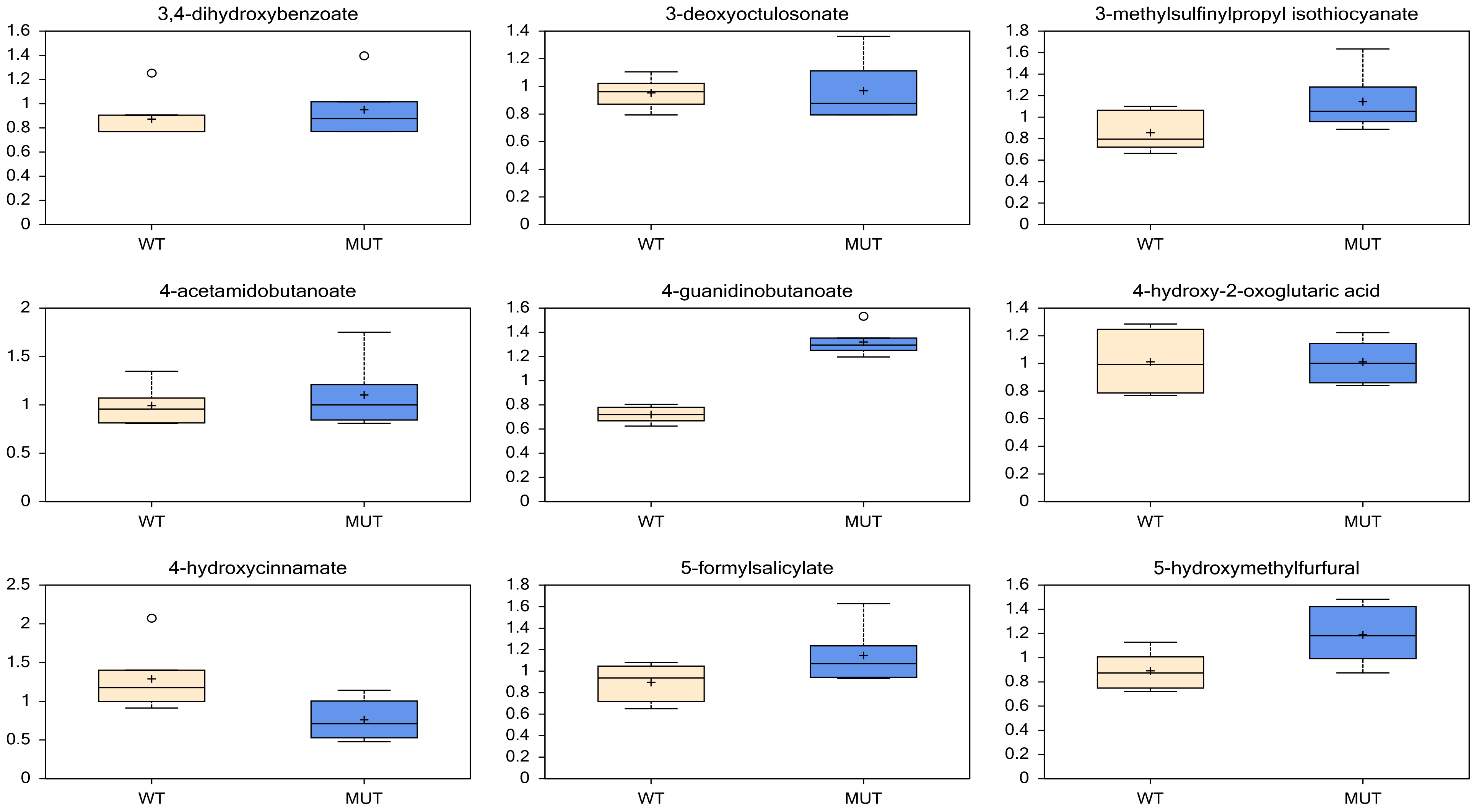
3,4-dihydroxybenzoate
3-deoxyoctulosonate
3-methylsulfinylpropyl isothiocyanate
4-acetamidobutanoate
4-guanidinobutanoate
4-hydroxy-2-oxoglutaric acid
4-hydroxycinnamate
5-formylsalicylate
5-hydroxymethylfurfural
WT
MUT

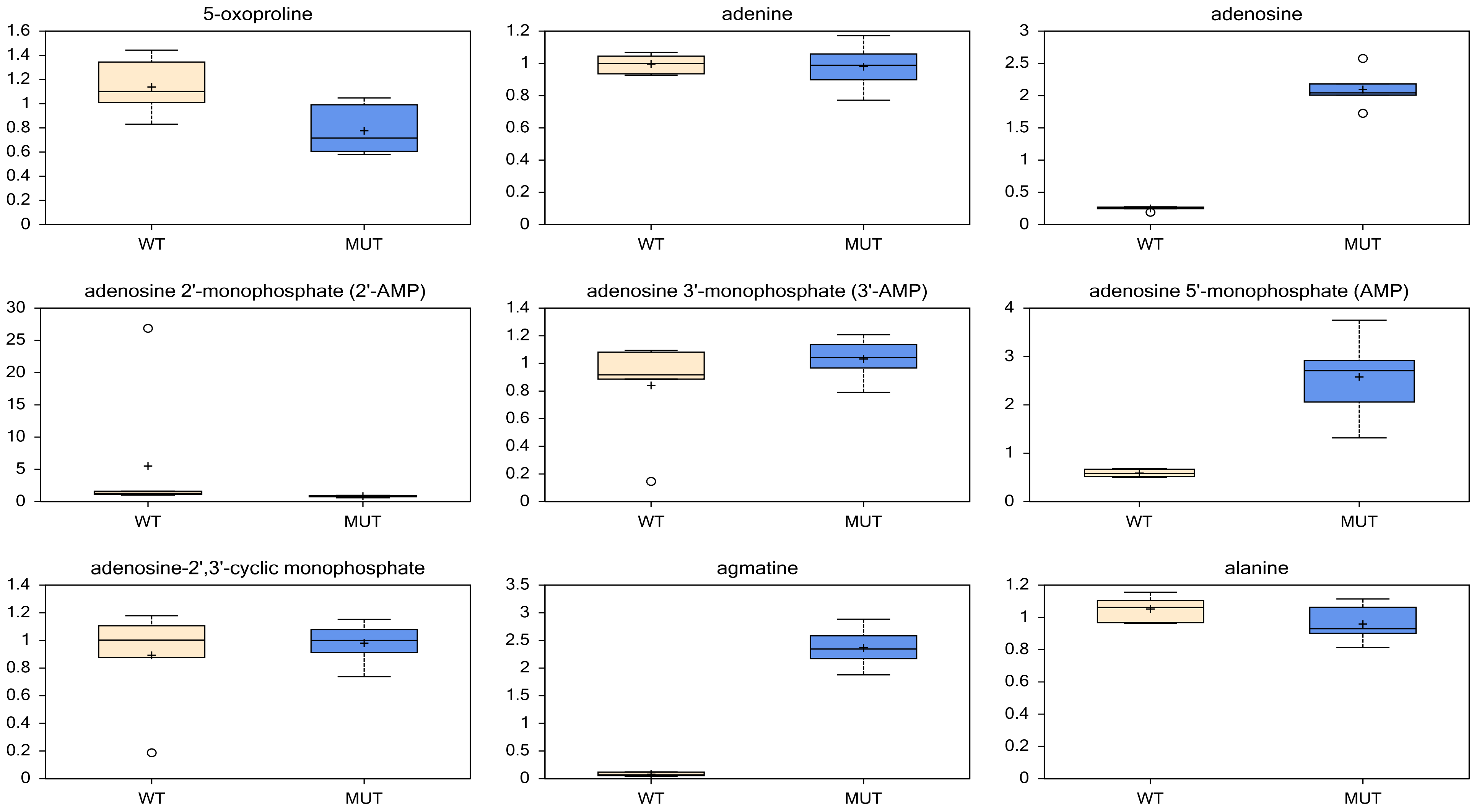
5-oxoproline
adenine
adenosine
adenosine 2'-monophosphate (2'-AMP)
adenosine 3'-monophosphate (3'-AMP)
adenosine 5'-monophosphate (AMP)
adenosine-2',3'-cyclic monophosphate
agmatine
alanine
WT
MUT

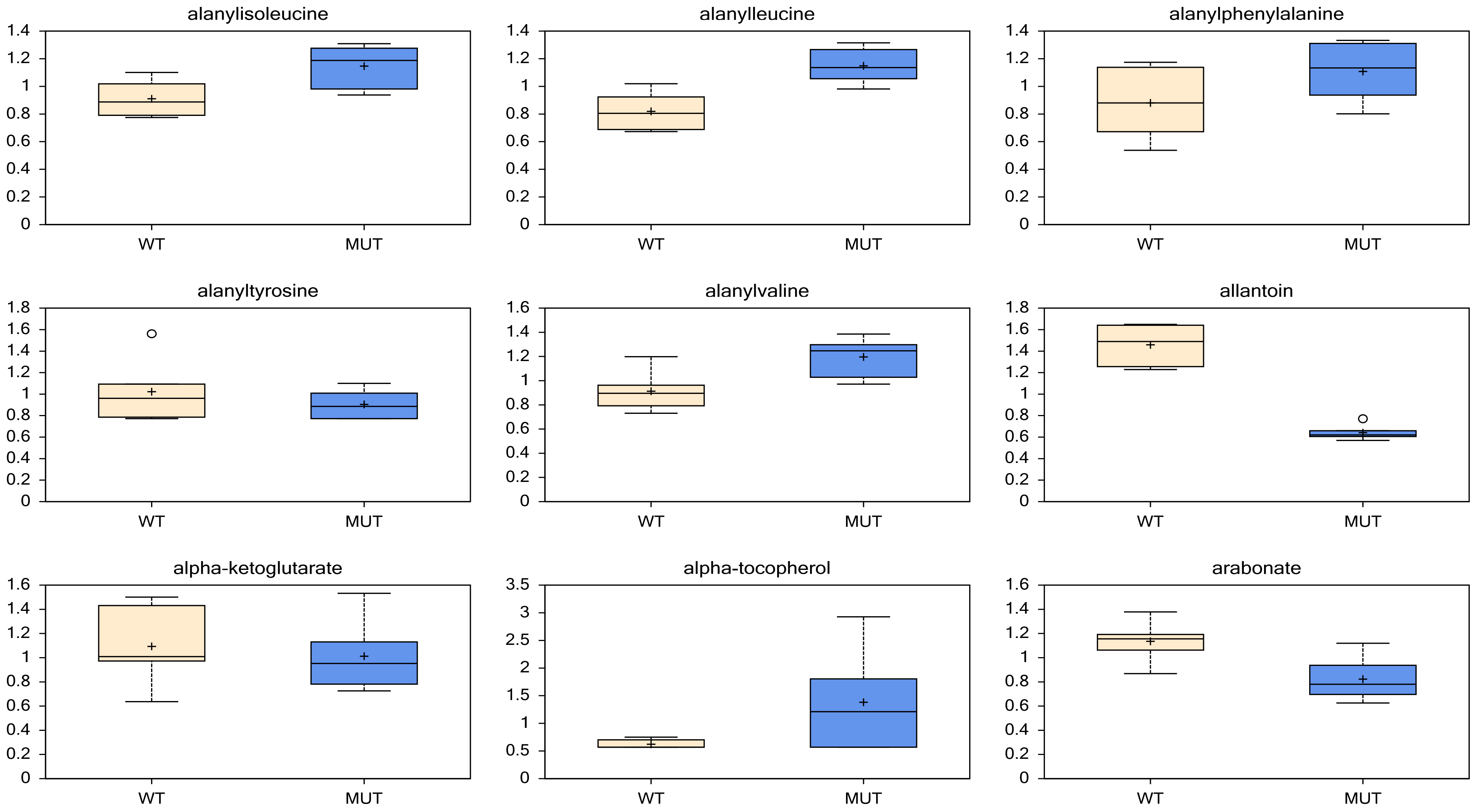

alanylisoleucine
alanylleucine
alanylphenylalanine
alanyltyrosine
alanylvaline
allantoin
alpha-ketoglutarate
alpha-tocopherol
arabonate
WT
MUT

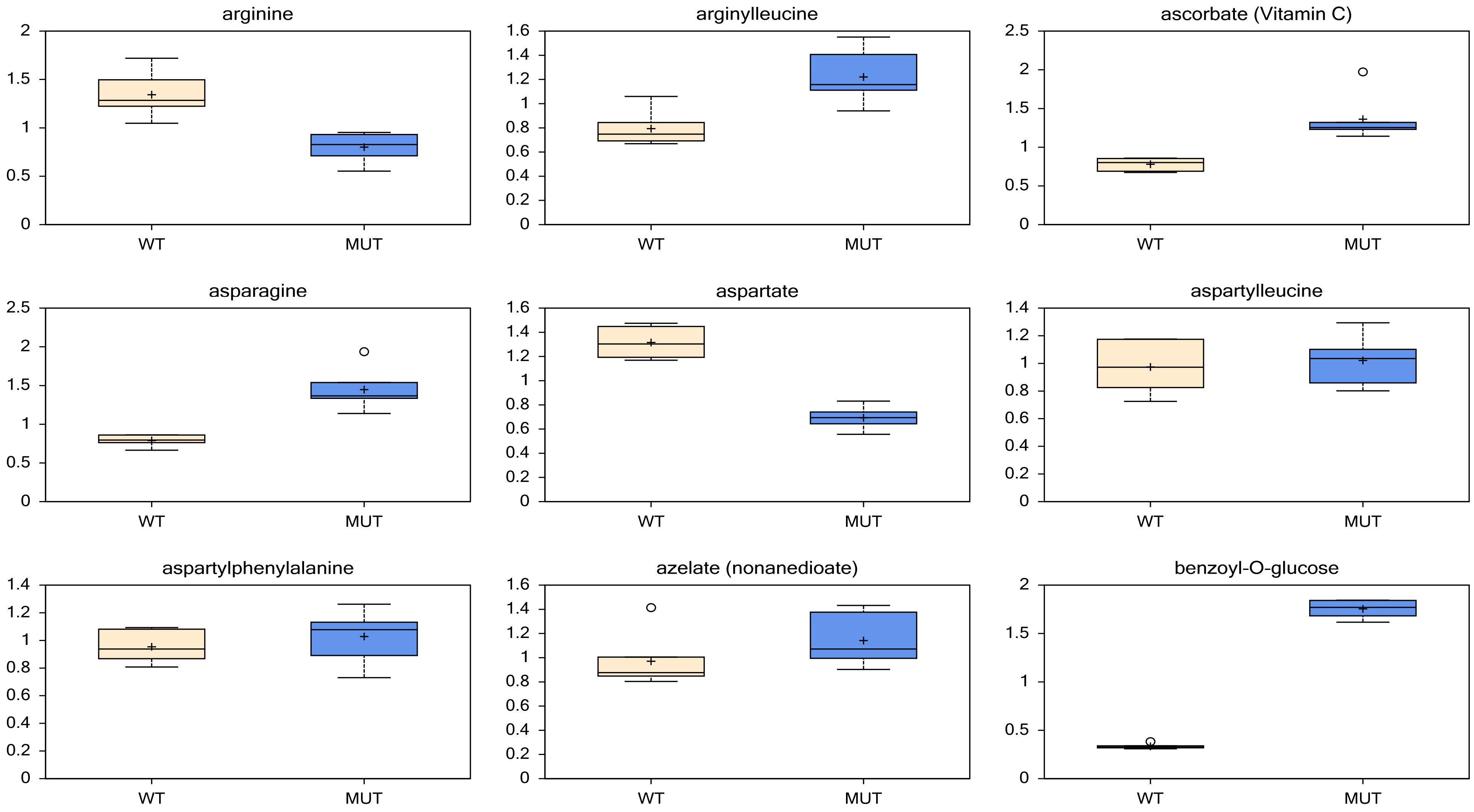
arginine
arginylleucine
ascorbate (Vitamin C)
asparagine
aspartate
aspartylleucine
aspartylphenylalanine
azelate (nonanedioate)
benzoyl-O-glucose
WT
MUT

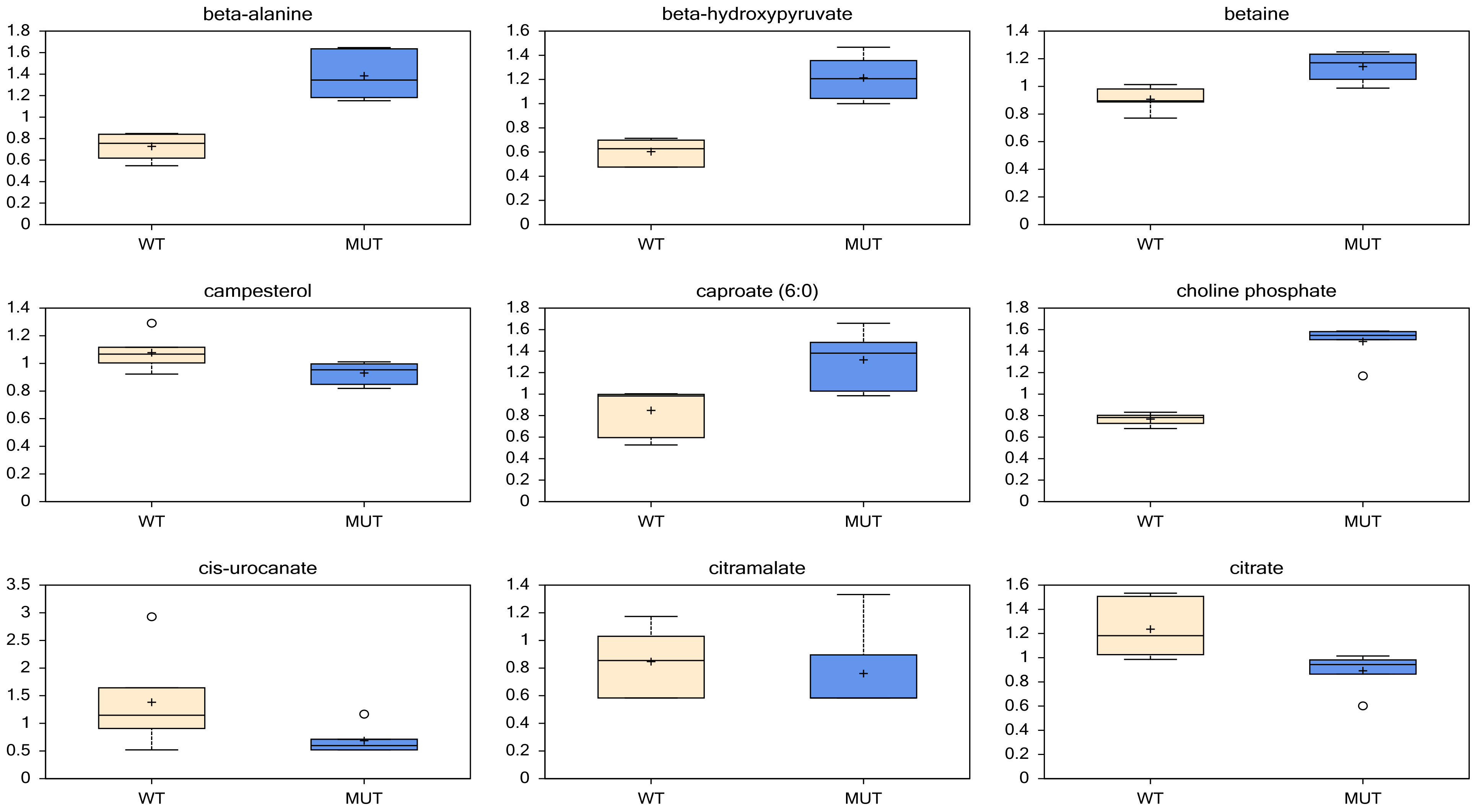
beta-alanine
beta-hydroxypyruvate
betaine
campesterol
caproate (6:0)
choline phosphate
cis-urocanate
citramalate
citrate
WT
MUT

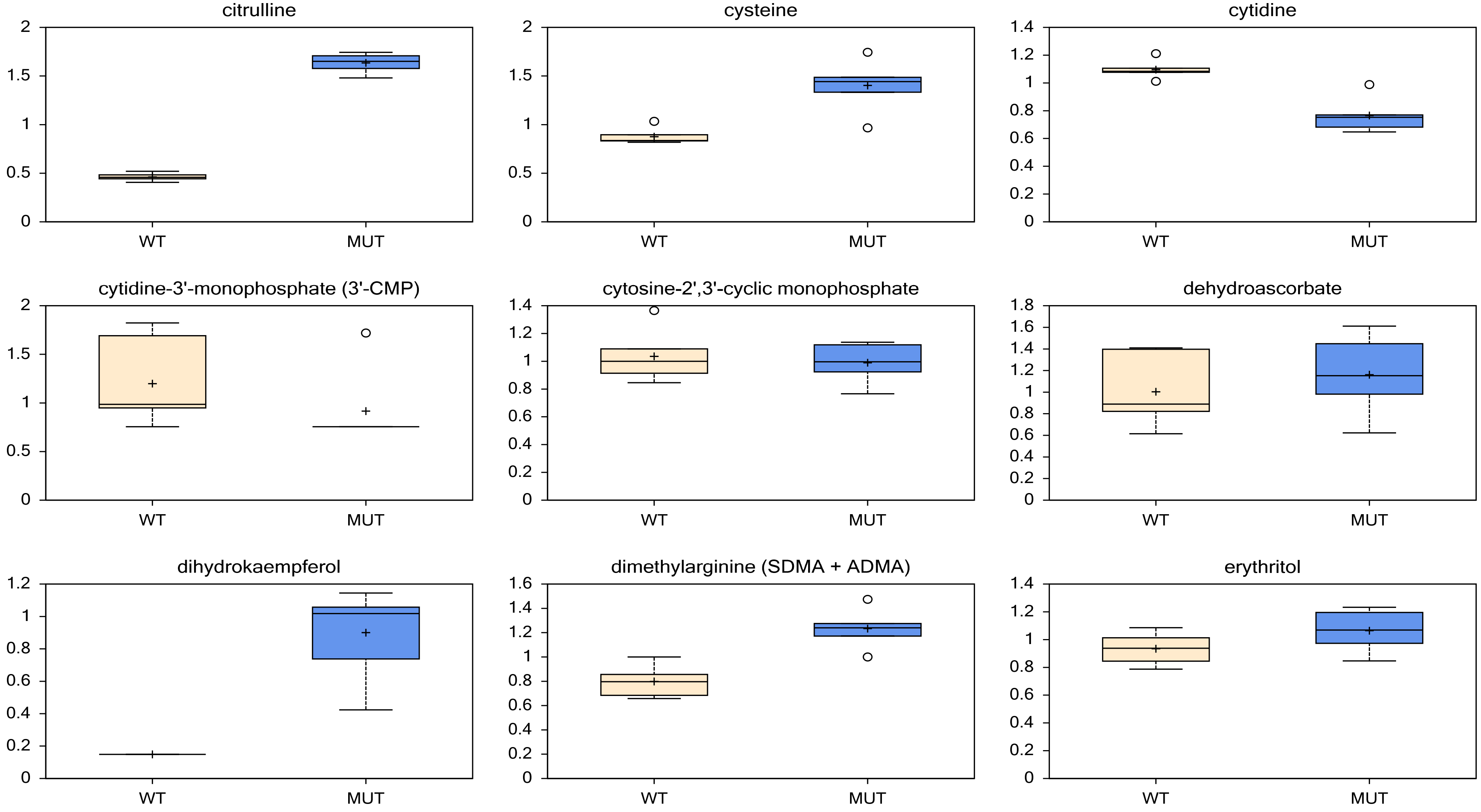
citrulline
cysteine
cytidine
cytidine-3'-monophosphate (3'-CMP)
cytosine-2',3'-cyclic monophosphate
dehydroascorbate
dihydrokaempferol
dimethylarginine (SDMA + ADMA)
erythritol
WT
MUT

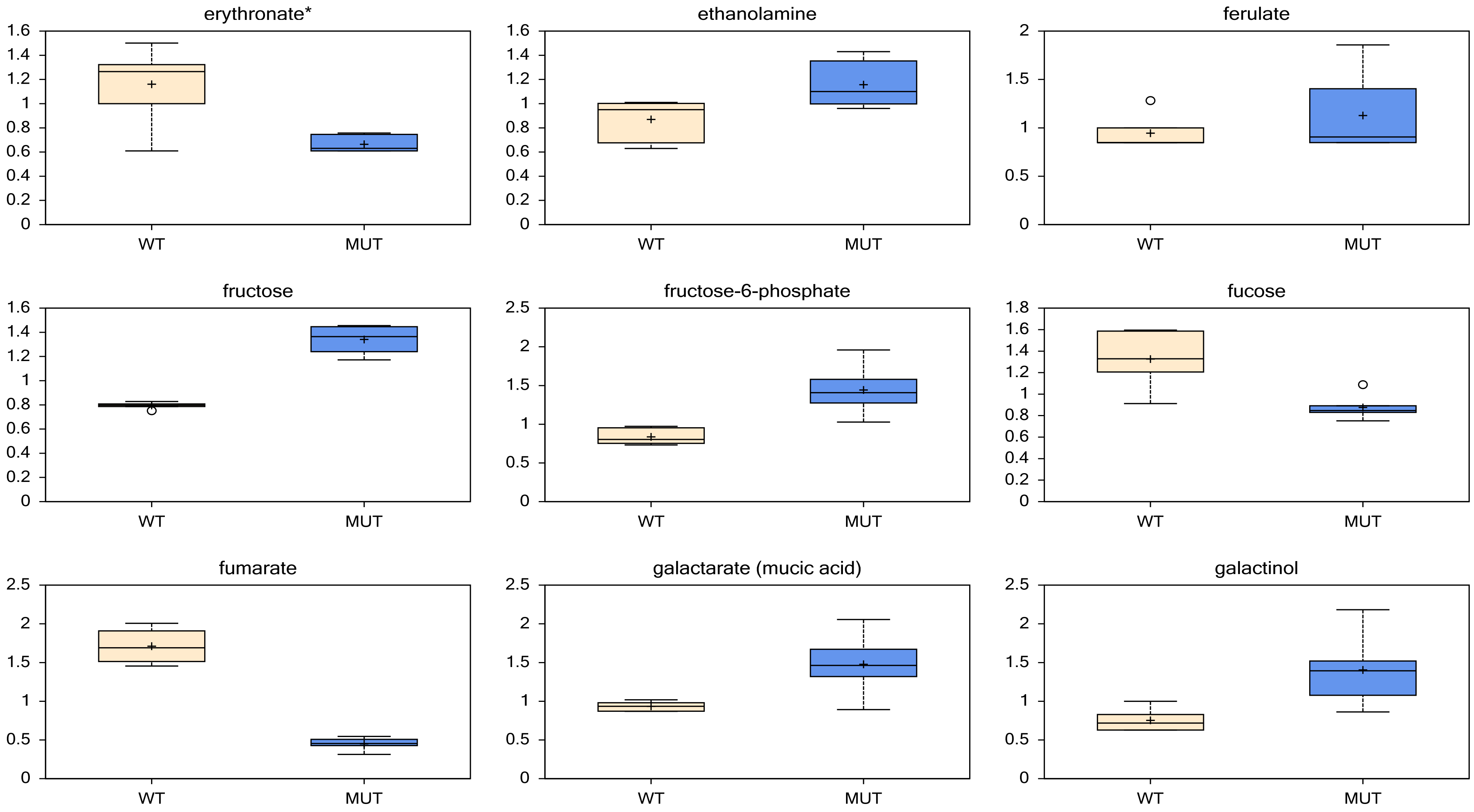
erythronate*
ethanolamine
ferulate
fructose
fructose-6-phosphate
fucose
fumarate
galactarate (mucic acid)
galactinol
WT
MUT

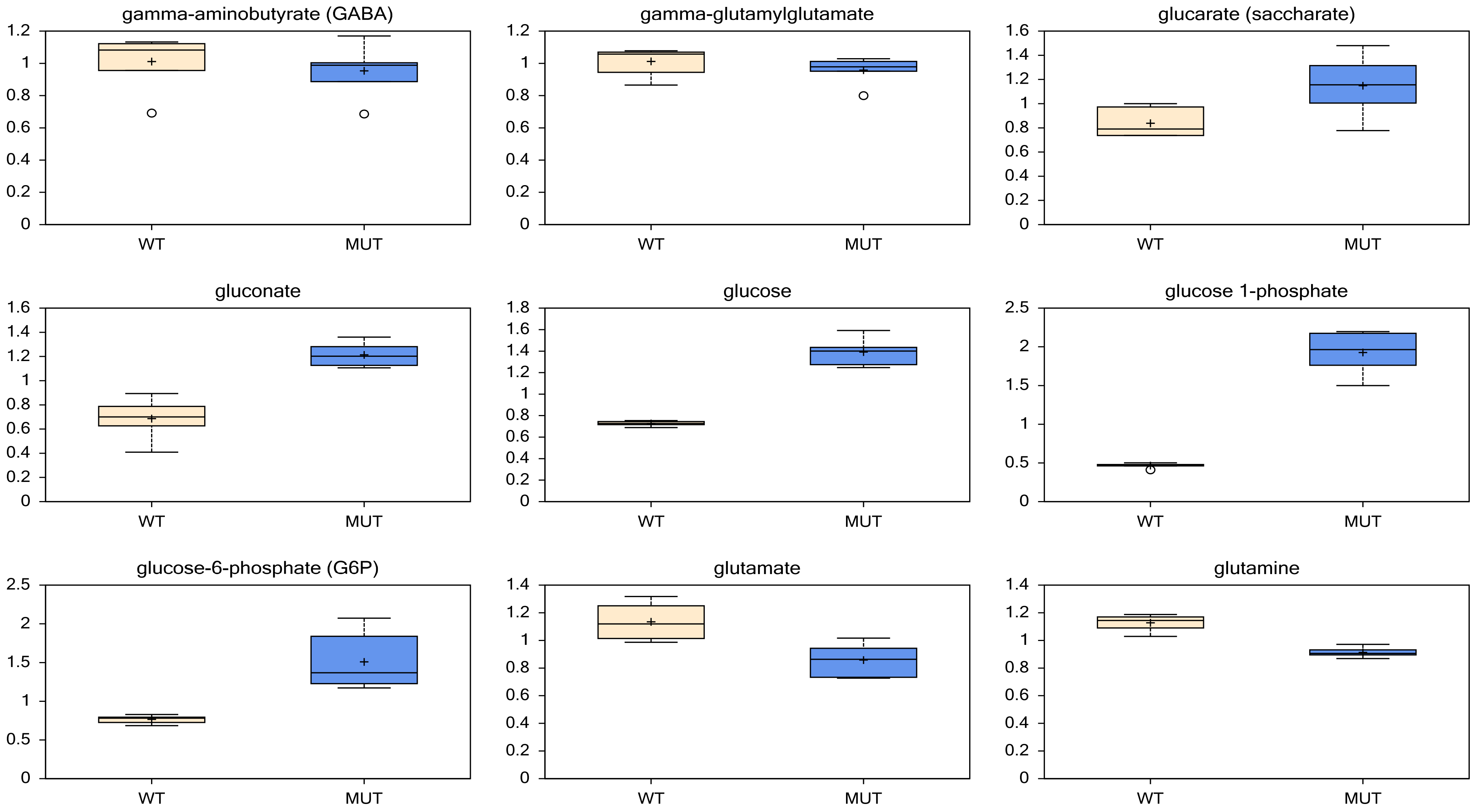
gamma-aminobutyrate (GABA)
gamma-glutamylglutamate
glucarate (saccharate)
gluconate
glucose
glucose 1-phosphate
glucose-6-phosphate (G6P)
glutamate
glutamine
WT
MUT

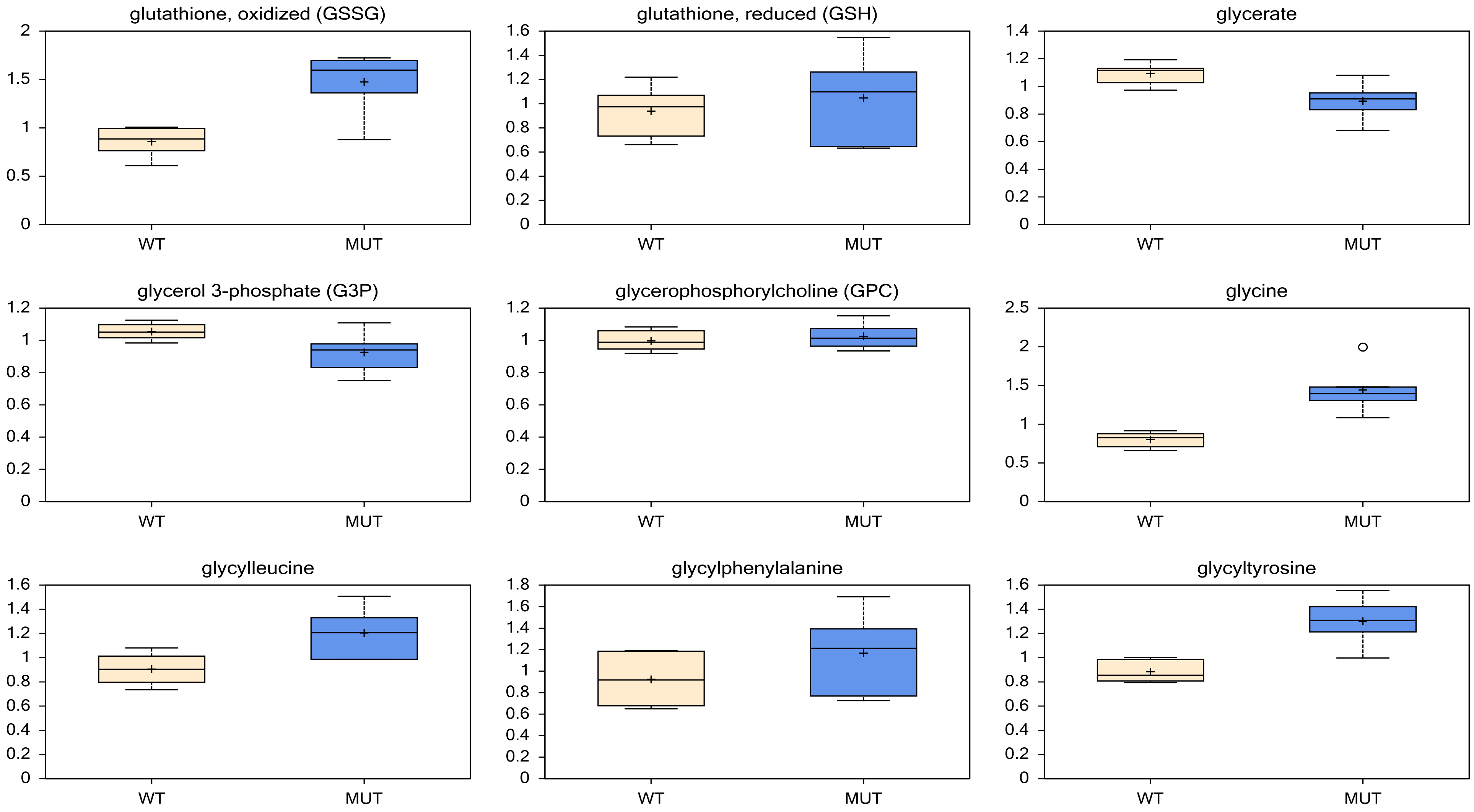
glutathione, oxidized (GSSG)
glutathione, reduced (GSH)
glycerate
glycerol 3-phosphate (G3P)
glycerophosphorylcholine (GPC)
glycine
glycylleucine
glycylphenylalanine
glycyltyrosine
WT
MUT

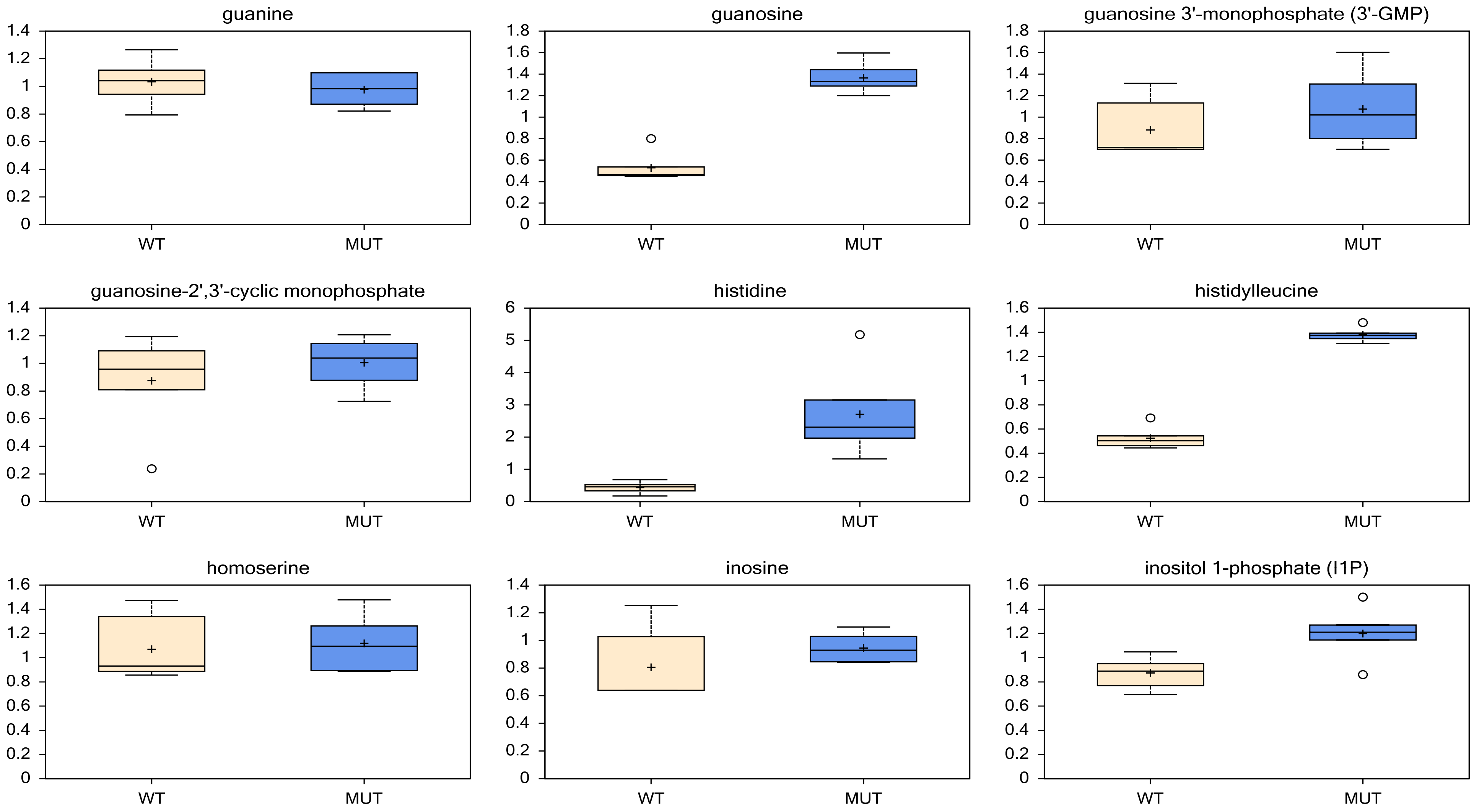
guanine
guanosine
guanosine 3'-monophosphate (3'-GMP)
guanosine-2',3'-cyclic monophosphate
histidine
histidylleucine
homoserine
inosine
inositol 1-phosphate (I1P)
WT
MUT

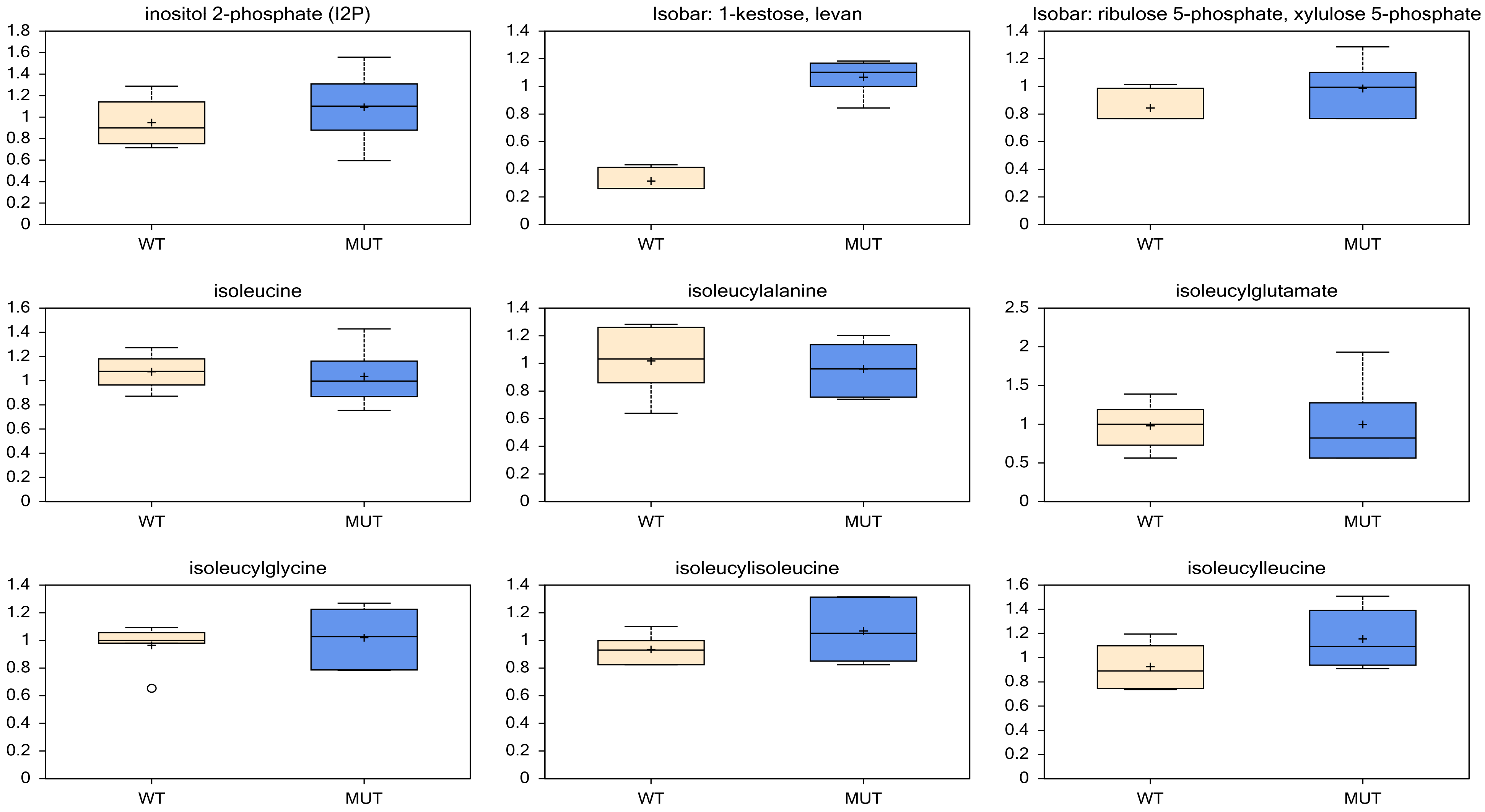

inositol 2-phosphate (I2P)
Isobar: 1-kestose, levan
Isobar: ribulose 5-phosphate, xylulose 5-phosphate
isoleucine
isoleucylalanine
isoleucylglutamate
isoleucylglycine
isoleucylisoleucine
isoleucylleucine
WT
MUT

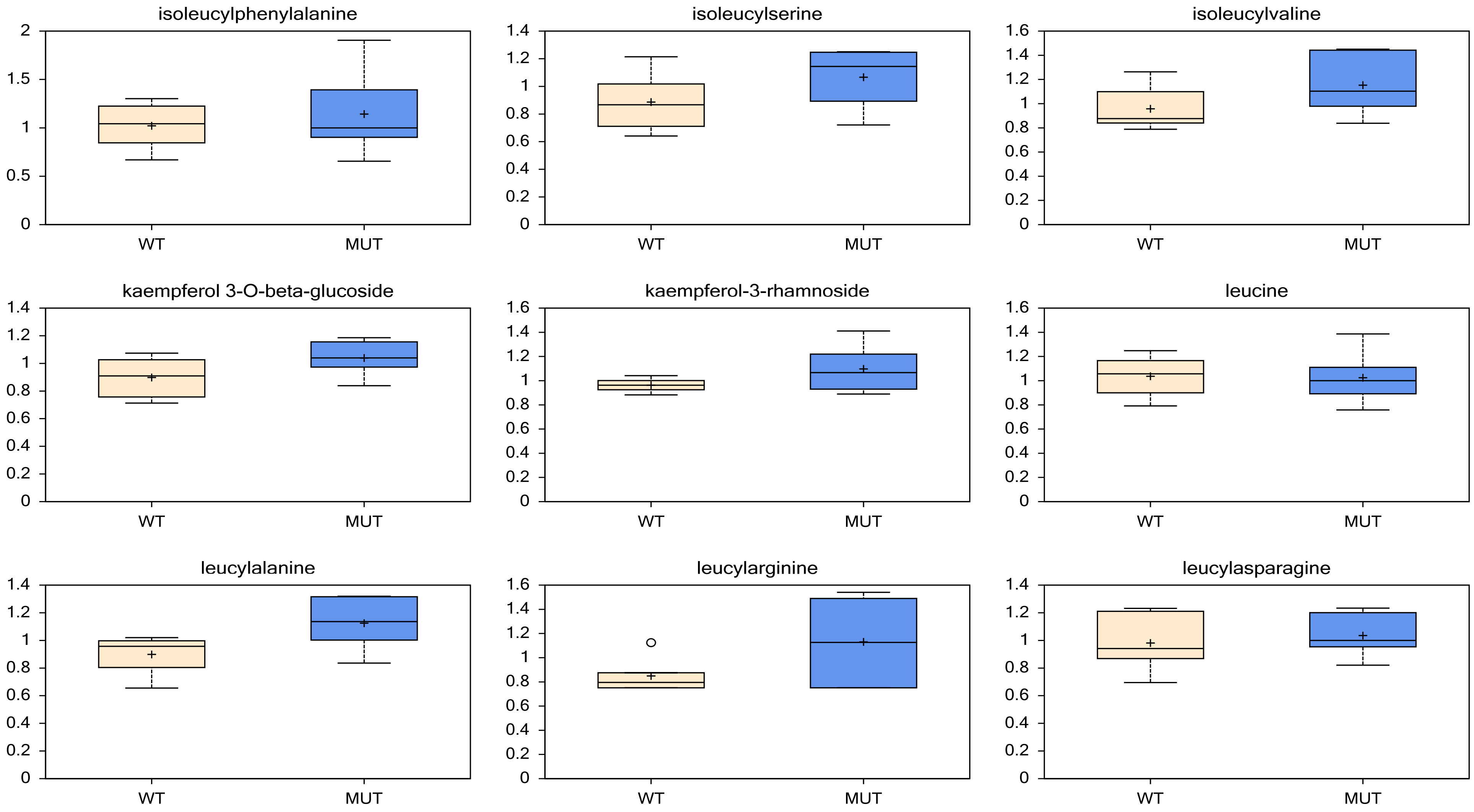

isoleucylphenylalanine
isoleucylserine
isoleucylvaline
kaempferol 3-O-beta-glucoside
kaempferol-3-rhamnoside
leucine
leucylalanine
leucylarginine
leucylasparagine
WT
MUT

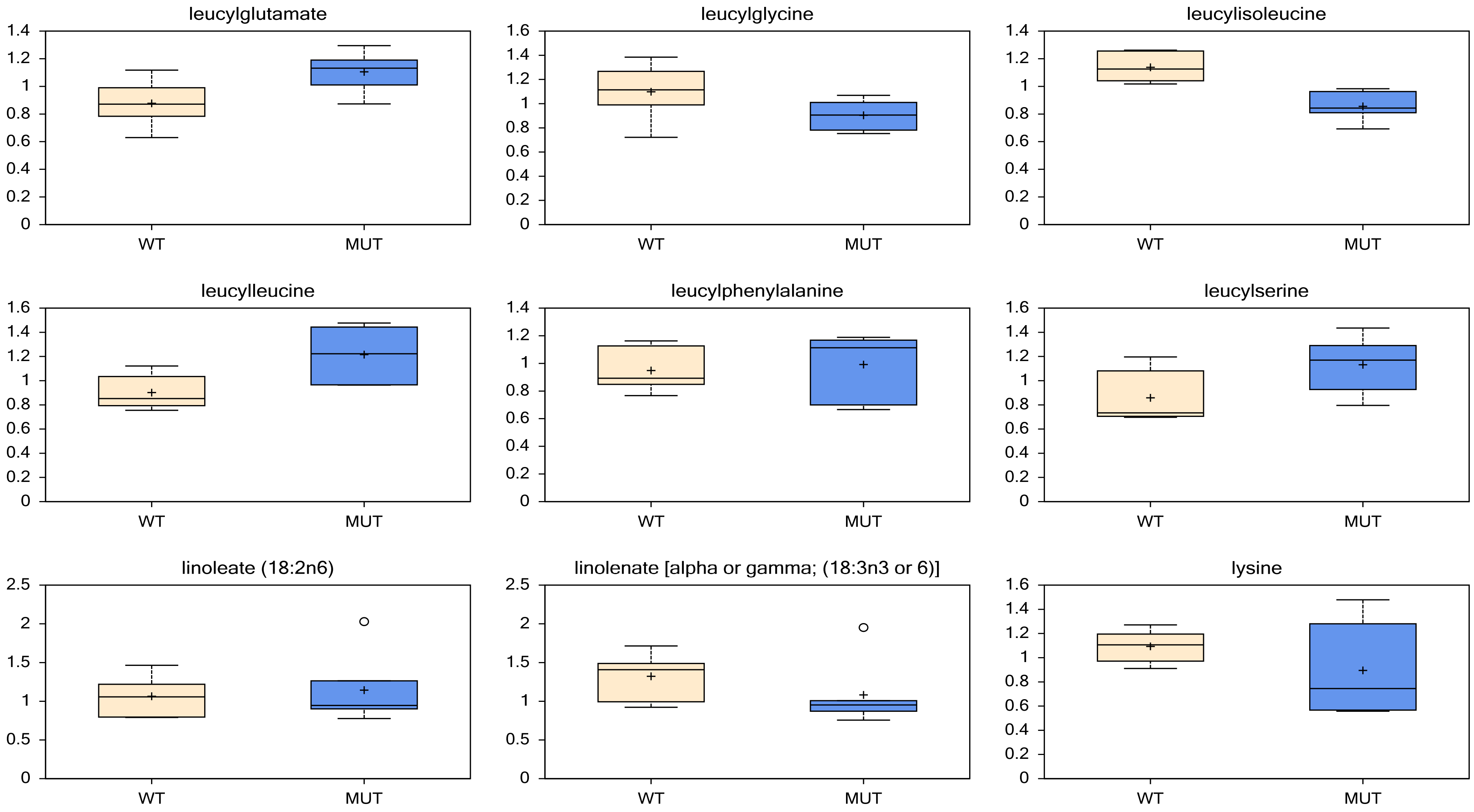

leucylglutamate
1.4
1.2
1
0.8
0.6
0.4
0.2
0
WT
MUT
leucylglycine
1.6
1.4
1.2
1
0.8
0.6
0.4
0.2
0
WT
MUT
leucylisoleucine
1.4
1.2
1
0.8
0.6
0.4
0.2
0
WT
MUT
leucylleucine
1.6
1.4
1.2
1
0.8
0.6
0.4
0.2
0
WT
MUT
leucylphenylalanine
1.4
1.2
1
0.8
0.6
0.4
0.2
0
WT
MUT
leucylserine
1.6
1.4
1.2
1
0.8
0.6
0.4
0.2
0
WT
MUT
linoleate (18:2n6)
2.5
2
1.5
1
0.5
0
WT
MUT
linolenate [alpha or gamma; (18:3n3 or 6)]
2.5
2
1.5
1
0.5
0
WT
MUT
lysine
1.6
1.4
1.2
1
0.8
0.6
0.4
0.2
0
WT
MUT

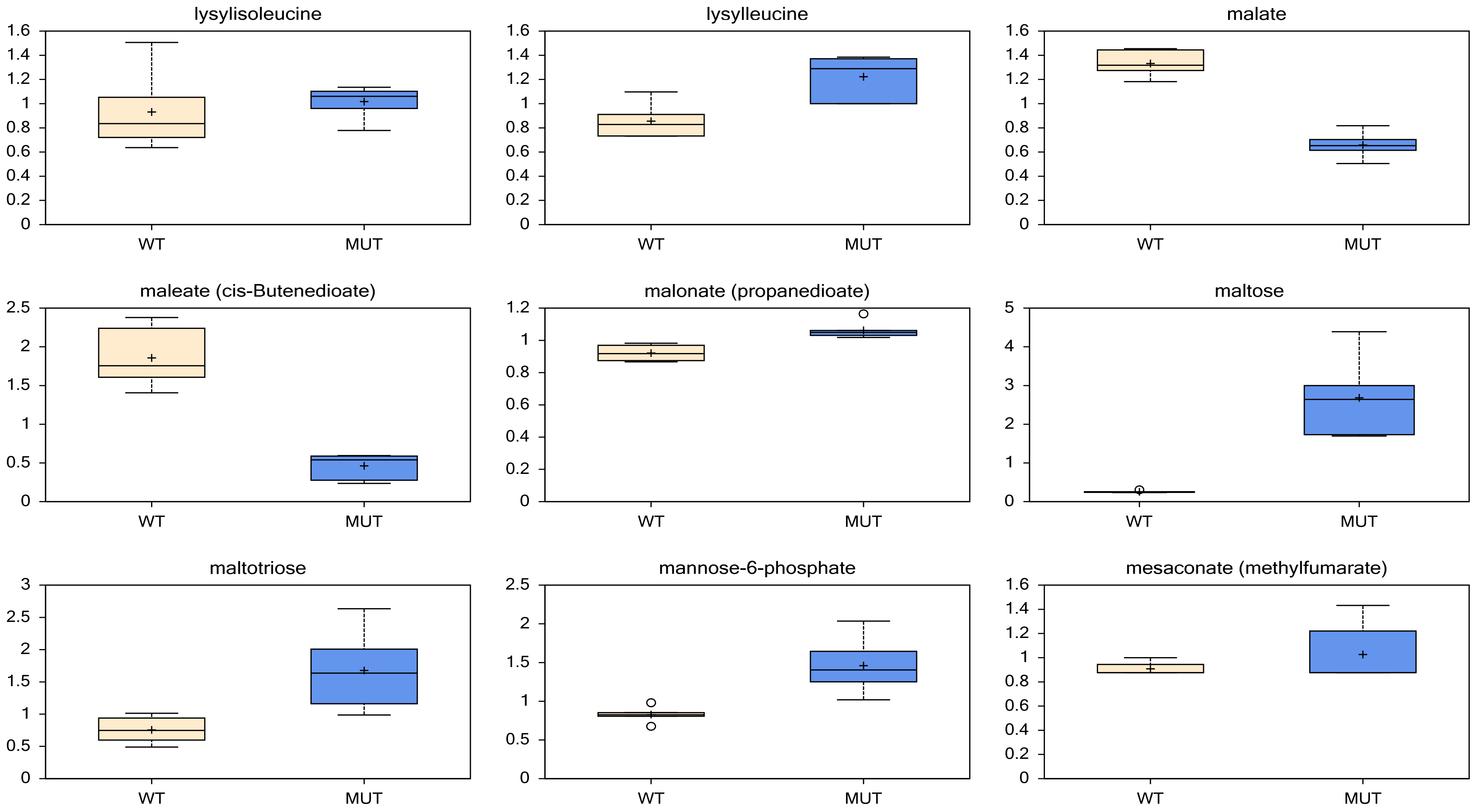
lysylisoleucine
lysylleucine
malate
maleate (cis-Butenedioate)
malonate (propanedioate)
maltose
maltotriose
mannose-6-phosphate
mesaconate (methylfumarate)
WT
MUT
0
0.2
0.4
0.6
0.8
1
1.2
1.4
1.6
0.5
1.5
2
2.5
3
4
5

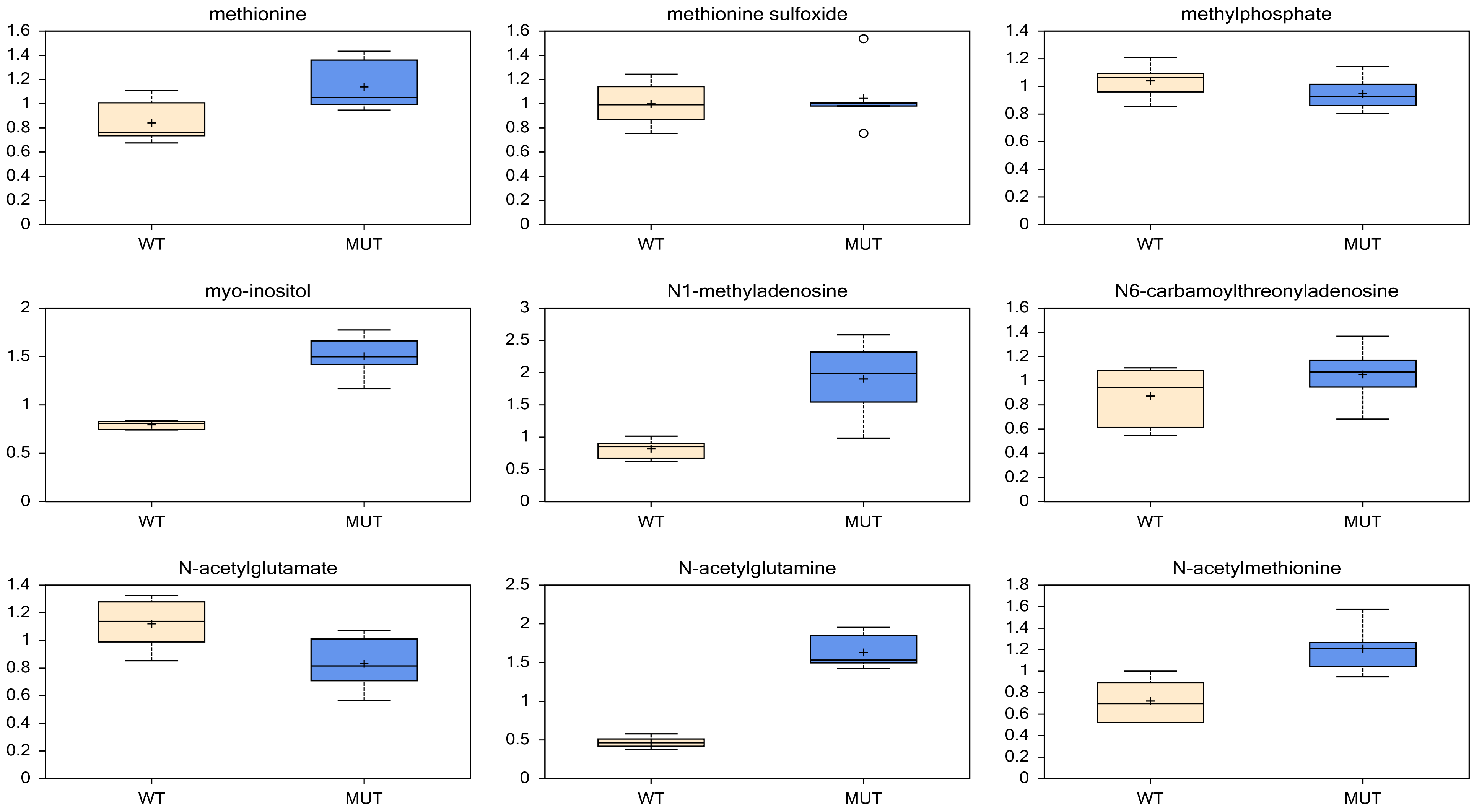
methionine
methionine sulfoxide
methylphosphate
myo-inositol
N1-methyladenosine
N6-carbamoylthreonyladenosine
N-acetylglutamate
N-acetylglutamine
N-acetylmethionine
WT
MUT

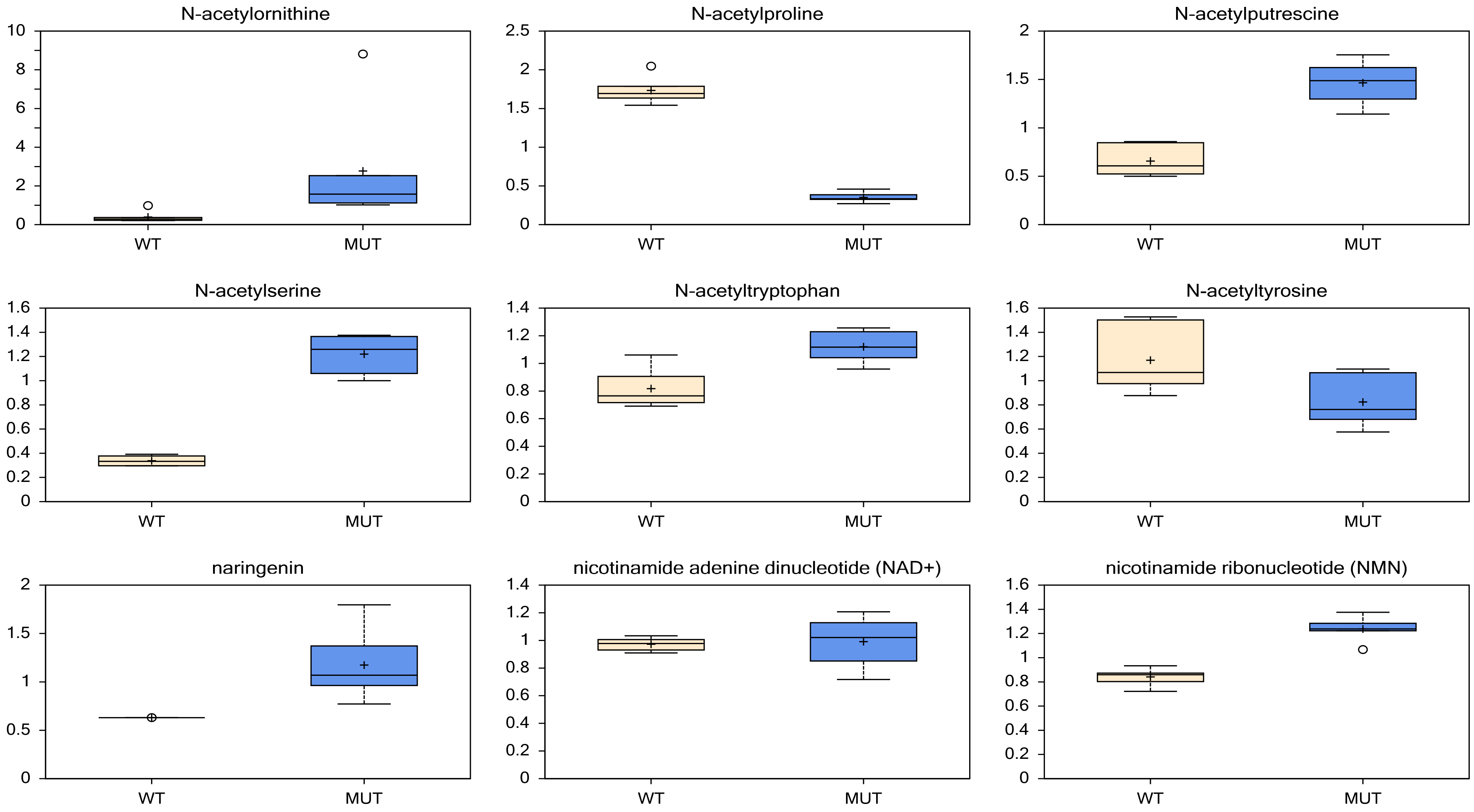
N-acetylornithine
N-acetylproline
N-acetylputrescine
N-acetylserine
N-acetyltryptophan
N-acetyltyrosine
naringenin
nicotinamide adenine dinucleotide (NAD+)
nicotinamide ribonucleotide (NMN)
WT
MUT

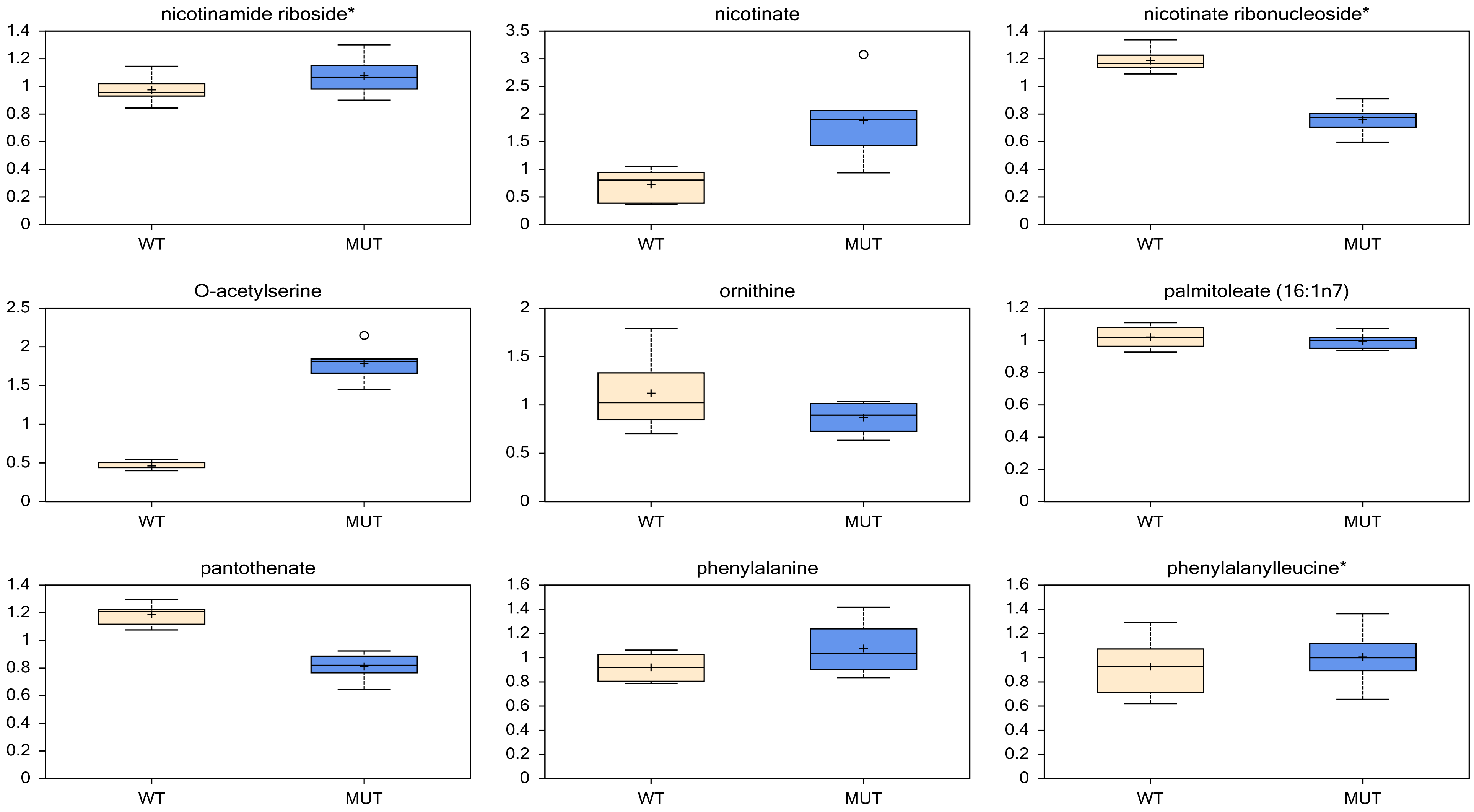
nicotinamide riboside*
nicotinate
nicotinate ribonucleoside*
O-acetylserine
ornithine
palmitoleate (16:1n7)
pantothenate
phenylalanine
phenylalanylleucine*
WT
MUT
0
0.2
0.4
0.5
0.6
0.8
1
1.2
1.4
1.5
1.6
2
2.5
3
3.5

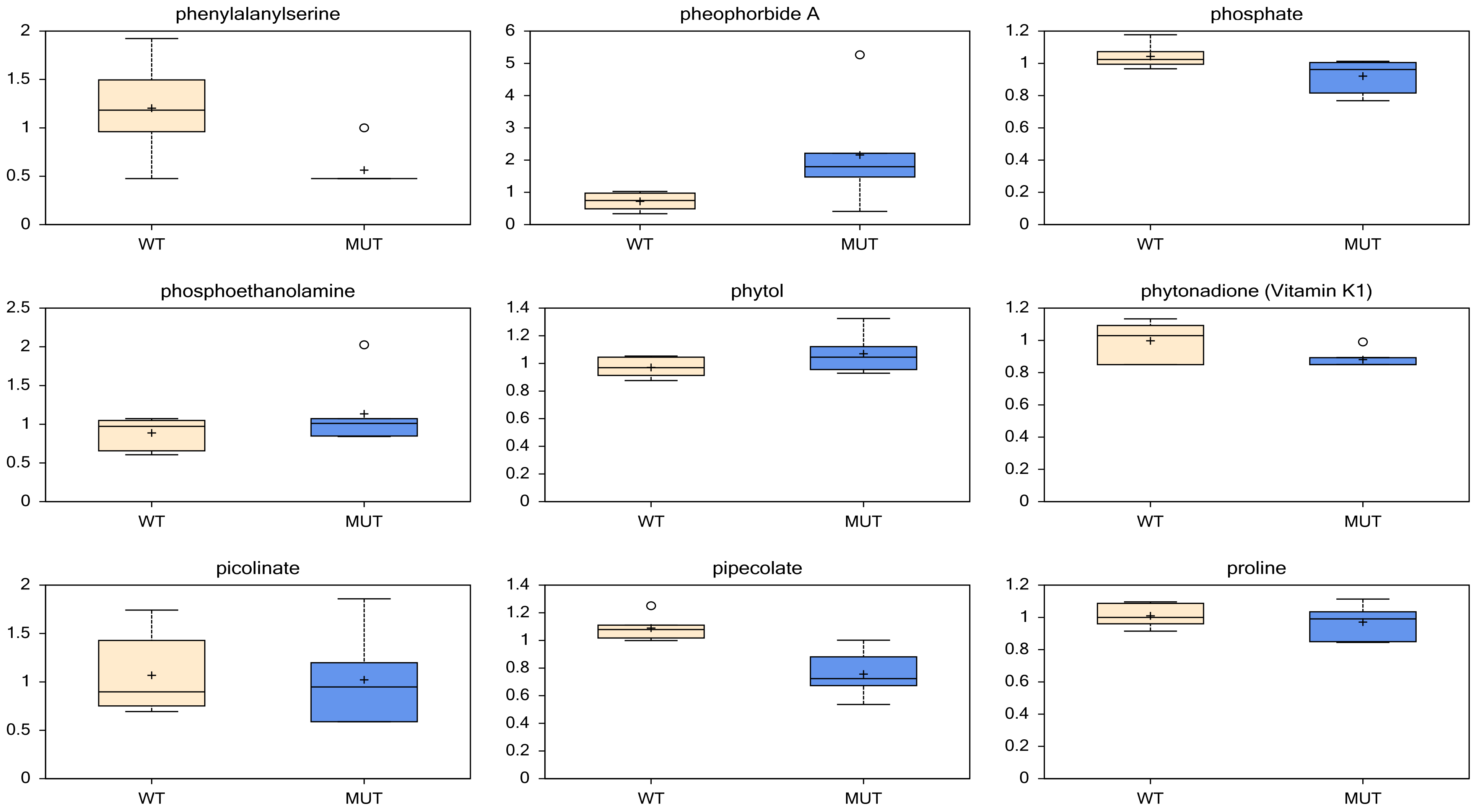
phenylalanylserine
pheophorbide A
phosphate
phosphoethanolamine
phytol
phytonadione (Vitamin K1)
picolinate
pipecolate
proline
WT
MUT

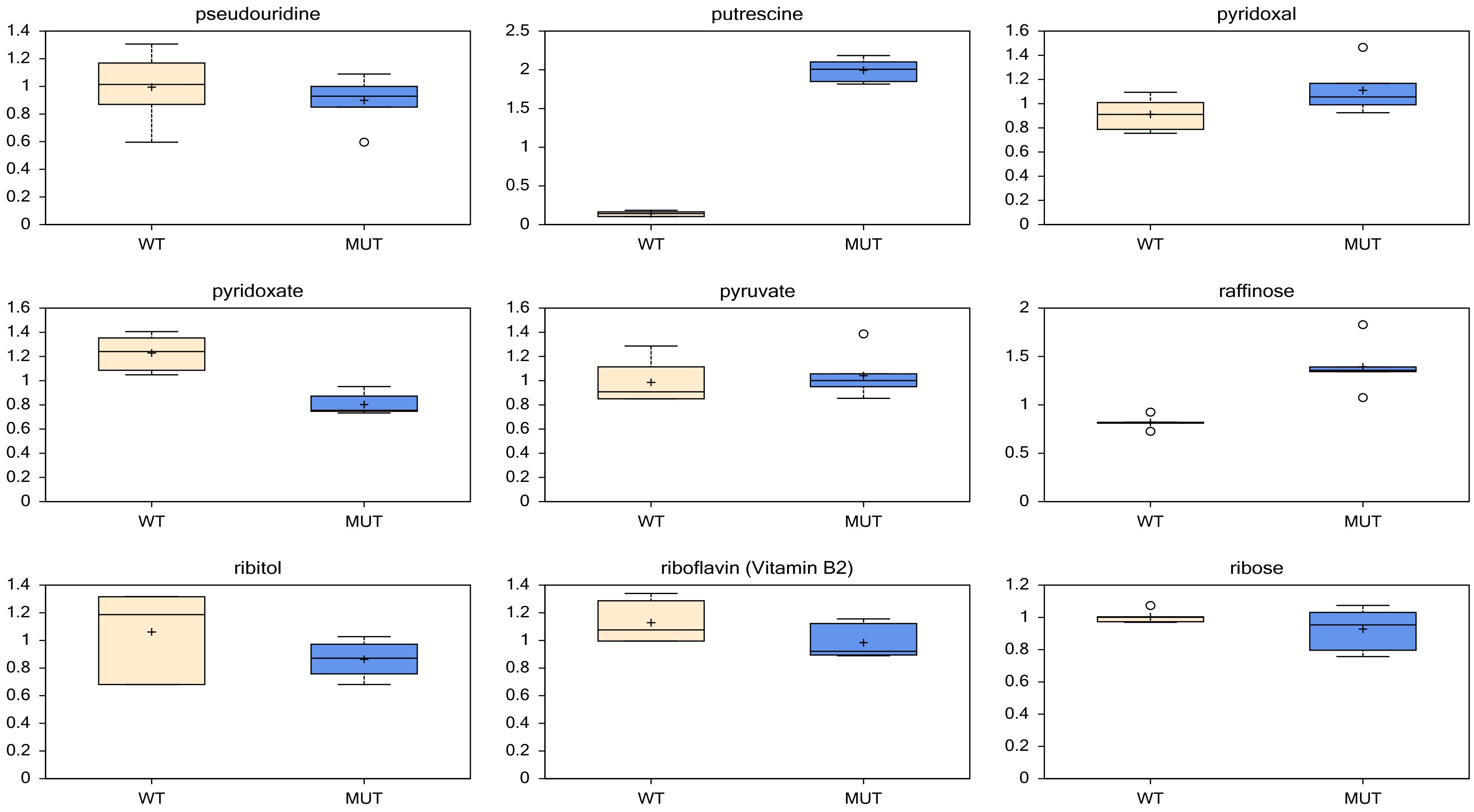
pseudouridine
putrescine
pyridoxal
pyridoxate
pyruvate
raffinose
ribitol
riboflavin (Vitamin B2)
ribose
WT
MUT

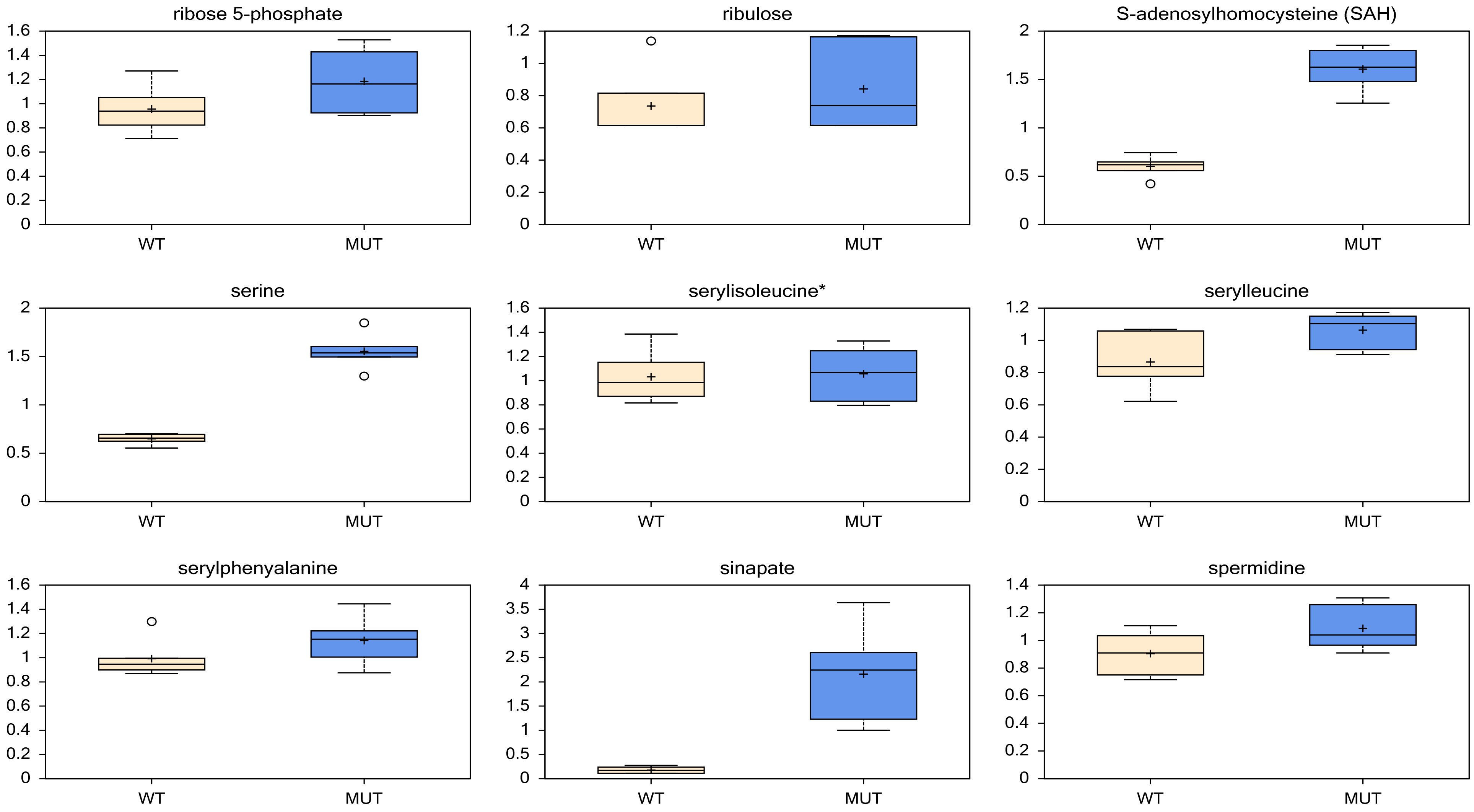
ribose 5-phosphate
ribulose
S-adenosylhomocysteine (SAH)
serine
serylisoleucine*
serylleucine
serylphenyalanine
sinapate
spermidine
WT
MUT

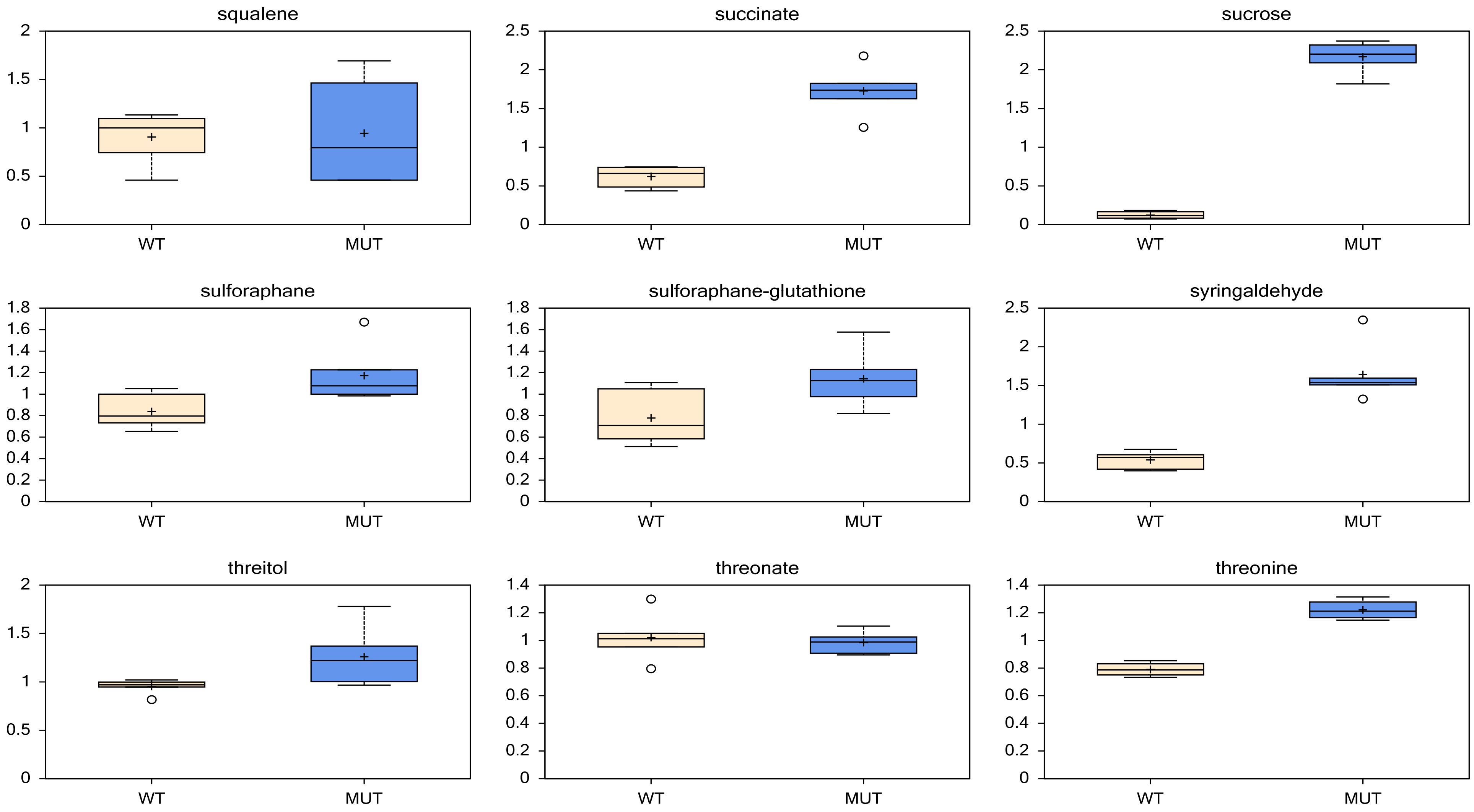

squalene
succinate
sucrose
sulforaphane
sulforaphane-glutathione
syringaldehyde
threitol
threonate
threonine
WT
MUT

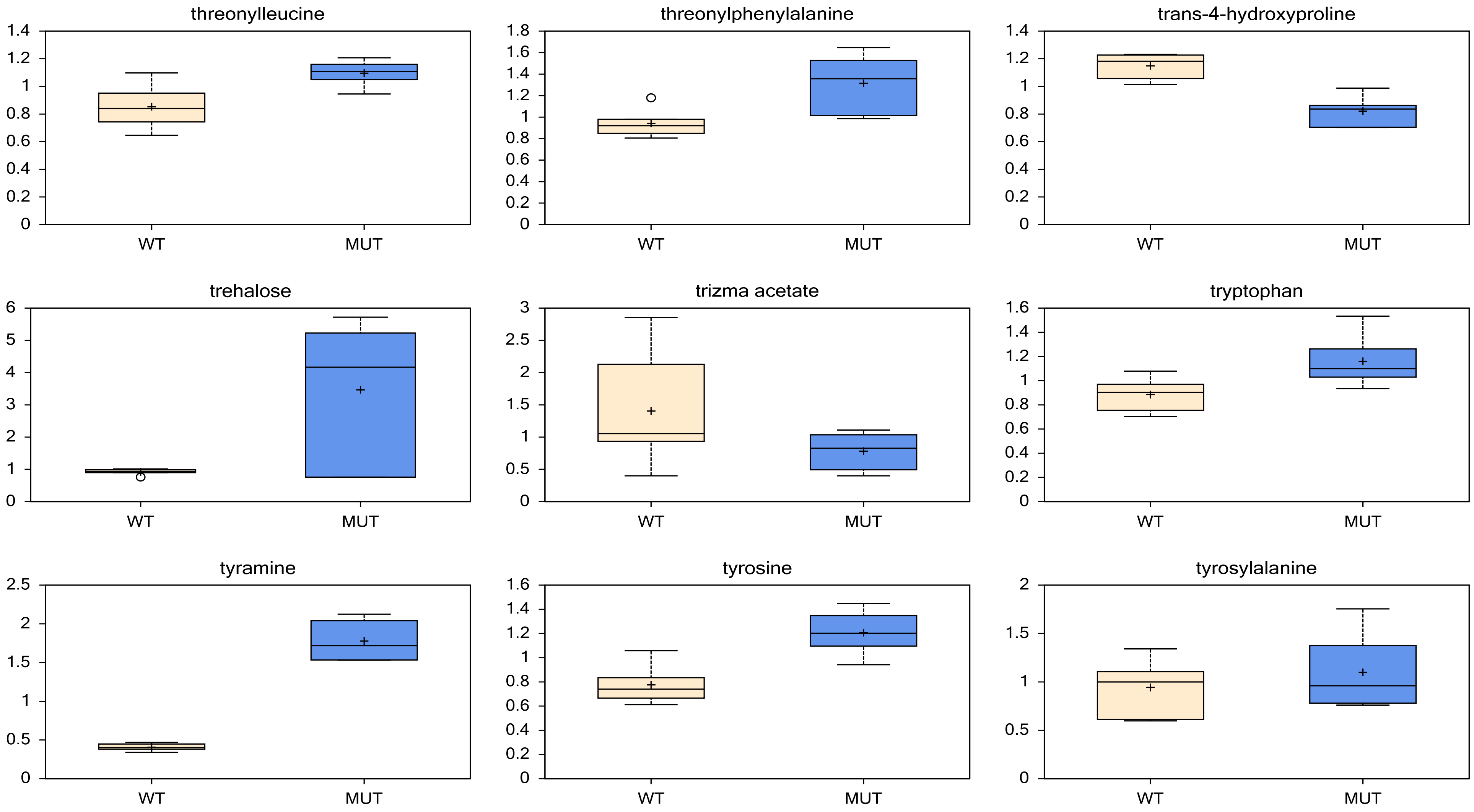
threonylleucine
threonylphenylalanine
trans-4-hydroxyproline
trehalose
trizma acetate
tryptophan
tyramine
tyrosine
tyrosylalanine
WT
MUT

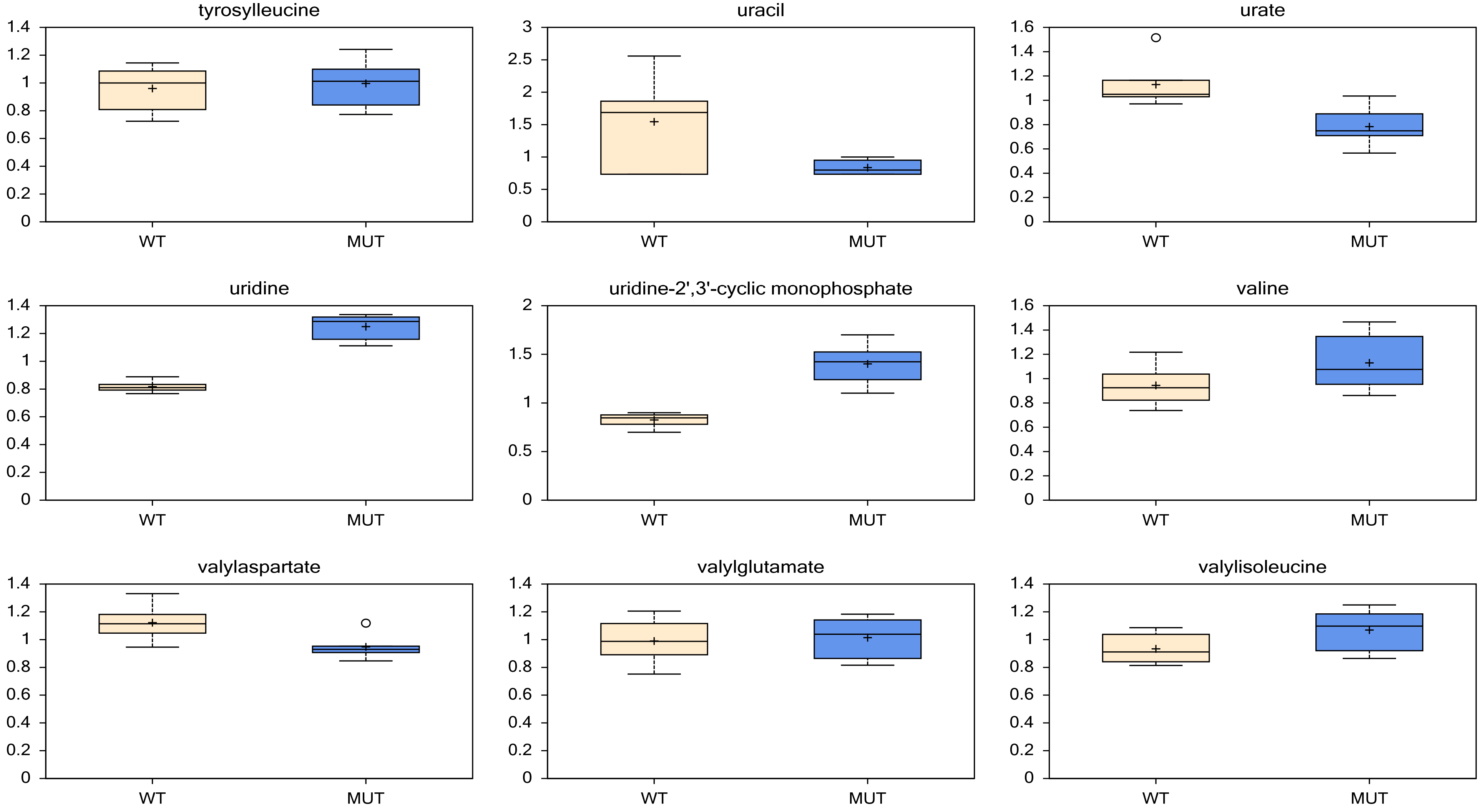

tyrosylleucine
uracil
urate
uridine
uridine-2',3'-cyclic monophosphate
valine
valylaspartate
valylglutamate
valylisoleucine
WT
MUT

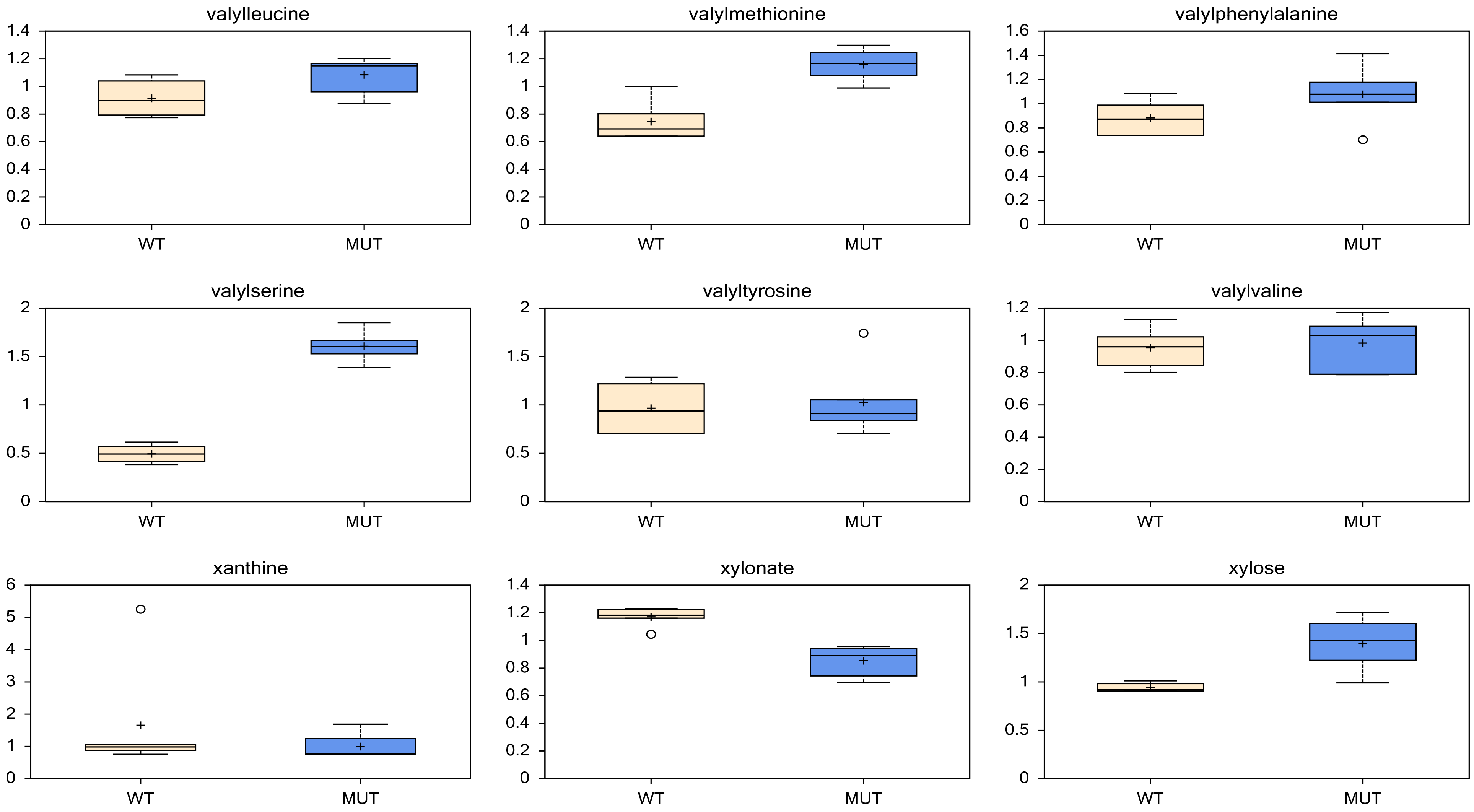
valylleucine
valylmethionine
valylphenylalanine
valylserine
valyltyrosine
valylvaline
xanthine
xylonate
xylose
WT
MUT
0
0.2
0.4
0.6
0.8
1
1.2
1.4
1.6
0.5
1.5
2
3
4
5
6

Table S3. Oligonucleotides used in this work.			
**Name**	**Sequence (5´to 3´)**	**AGI**	**Application**
qP-ACT2-R	TGTCTCGTGGATTCCAGCAG	AT3G18780	qRT-PCR
qP-ACT2-F	TTGTTCCAGCCCTCGTTTGT	AT3G18780	qRT-PCR
LB3	TAGCATCTGAATTTCATAACCAATCTCG ATACAC		Genotyping SAIL T
AtNOS1.1-RP	GCACCTACACCACAGGCAAGC	AT3G47450	Genotyping Mutation
AtNOS1.1-LP	CCAATTGGCAATGTTGGTCG	AT3G47450	Genotyping Mutation
NR2.1-RP	ACCTTCTTCGTCGGCGAGTTC	AT1G37130	Genotyping Mutation
NR2.1-LP	ACGGCGTGGTTCGTTCTTACA	AT1G37130	Genotyping Mutation
CAPS_nr1-R	GGCTATAGATCCCGCATCGAC	AT1G77760	Genotyping Mutation
CAPS_nr1-F	TACGACGACTCCTCAAGCGAC	AT1G77760	Genotyping Mutation
qADH1-R	AGGATGTGGGTGACCTTGTC	AT1G77120	qRT-PCR
qADH1-F	GCACCTGCTGAACATCAAGA	AT1G77120	qRT-PCR
q-NCED3-R	CAGAAGCAATCTGGAGCATCAA	AT3G14440	qRT-PCR
q-NCED3-F	CGGTGGTTTACGACAAGAACAA	AT3G14440	qRT-PCR
qCHI-R	GACGGTGAAGATCACGAATTTACC	AT3G55120	qRT-PCR
qCHI-F	ATGTCTTCATCCAACGCCTGCGCC	AT3G55120	qRT-PCR
qCHS-R	TGCATGTGACGTTTCCGAATTGTCGAC	AT5G13930	qRT-PCR
qCHS-F	AGCTGATGGACCTGCAGGCATCTTGGC	AT5G13930	qRT-PCR
qSnRK2.9-R	CACAAGCTCGTTTGTTTGCTTATT	AT2G23030	qRT-PCR
qSnRK2.9-F	GGAGAAGTATGAGATGGTGAAGGATT	AT2G23030	qRT-PCR
qHAI1-R	CTCGCACCGGCATTTTG	AT5G59220	qRT-PCR
qHAI1-F	ACGCGCATGGACATGGA	AT5G59220	qRT-PCR
qDFR-R	GTGAGTAGCGTCTTGGCGTTTGGC	AT5G42800	qRT-PCR
qDFR-F	CGTGCCACCGTTCGAGATCC	AT5G42800	qRT-PCR
qPAP1-R	GCACCGGTTTAGCCCAGCTCTTAC	AT1G56650	qRT-PCR
qPAP1-F	GAGGGTTCGTCCAAAGGGCTGCG	AT1G56650	qRT-PCR
qCYP707A3-R	TGATGTGCCAAAGGAGGACG	AT5G45340	qRT-PCR
qCYP707A3-F	TCTTTTGGGAATGAAGGGTCTG	AT5G45340	qRT-PCR
qLTI65/RD29b-R	TCAGTTCCCAGAATCTTGAACT	AT5G52300	qRT-PCR
qLTI65/RD29b-F	CTTGGCACCACCGTTGGGACTA	AT5G52300	qRT-PCR
qF3H/TT7-R	GATCTGACGGCAGATCTCTCCTCTTTT	AT3G51240	qRT-PCR
qF3H/TT7-F	ATGGCTCCAGGAACTTTGACTGAGCTA	AT3G51240	qRT-PCR
qNIA1-F	AGGTTTGGAAGGCGAATCG	AT1G77760	qRT-PCR
qNIA1-R	TGGCTGCAACGCAAACTG	AT1G77760	qRT-PCR
qNIA2-F	CCCGTTGCACTACGTTCGTA	AT1G37130	qRT-PCR
qNIA2-R	CGTCCATTCGGCCCATT	AT1G37130	qRT-PCR
qAtNOA-2nd-F	CAATGAAGCAGAGCATGAGATACA	AT3G47450	qRT-PCR
qAtNOA-2nd-F	TGGCAATGTTGGTCGAAGAA	AT3G47450	qRT-PCR
